# Regularisation by multiplicative noise for reaction–diffusion equations

**DOI:** 10.1007/s00440-026-01474-0

**Published:** 2026-03-07

**Authors:** Konstantinos Dareiotis, Teodor Holland, Khoa Lê

**Affiliations:** School of Mathematics, University of Leeds, Leeds, UK

**Keywords:** 60H15, 60H50

## Abstract

We consider the stochastic reaction–diffusion equation in 1+1 dimensions driven by multiplicative space–time white noise, with a distributional drift belonging to a Besov–Hölder space with any regularity index strictly larger than -1. We assume that the diffusion coefficient is a regular function which is bounded away from zero. By using a combination of stochastic sewing techniques and Malliavin calculus, we show that the equation admits a unique solution.

## Introduction

This paper is concerned with regularisation by noise phenomena for the stochastic reaction–diffusion equation on the 1-dimensional torus $\mathbb {T} := \mathbb {R}/\mathbb {Z}$ driven by multiplicative space–time white noise

1.1 $$\begin{aligned} (\partial _t - \Delta )u = b(u) + \sigma (u)\xi , \qquad u|_{t=0}=u_0. \end{aligned}$$

We show existence and uniqueness of solutions and we derive stability estimates, provided that σ is regular, bounded away from zero and that *b* is a distribution with a regularity index strictly larger than -1 in the Besov–Hölder scale.

For results on regularisation by noise phenomena for stochastic differential equations (SDEs), we refer the reader to [8, 17, 27, 34, 36]. Concerning stochastic partial differential equations (SPDEs), the first results on regularisation by noise can be traced back to the works of Gyöngy and Pardoux [21, 25]. Therein, the authors consider SPDEs of the form

1.2 $$\begin{aligned} (\partial _t - \Delta )u = b(u) + \xi , \qquad u|_{t=0}=u_0, \end{aligned}$$

which corresponds to (1.1) with σ=1. It is well known that the deterministic counterpart of (1.2) admits a unique solution provided that *b* is a Lipschitz continuous function. Without Lipschitz regularity, solutions may not exist or may not be identified uniquely. The situation changes in the presence of noise. It is shown in [21] and [25] that (1.2) admits a unique strong solution provided that *b* is merely the sum of a bounded measurable function and an $L_p$-integrable function with some p≥2. Similar results were obtained for SPDEs in an abstract Hilbert-space framework with bounded and measurable drift in [11]. In [7], Butkovsky and Mytnik show when b is bounded and measurable, path-by-path uniqueness also holds for (1.2). For such drift, discrete approximation schemes for the solution of (1.2) have been established with an optimal rate in [6], quantifying earlier results from [23, 24].

Notice that in all the previous results, *b* is quite irregular, nevertheless it is a function. The first well-posedness result which accommodates distributional drift *b* is due to Athreya, Butkovsky, Mytnik, and the third co-author in [2]. In such case, the composition *b*(*u*) is not well-defined a priori and solutions to (1.2) are defined in a regularised sense which is similar to that of Bass and Chen in [5] (see Definition 3.2). They show in [2] that (1.2) admits a unique probabilistically strong solution provided that *b* belongs to the Besov space $\mathscr {B}_{q,\infty }^\alpha$ for some $q \in [1,\infty ]$, with

1.3 $$\begin{aligned} \alpha - 1/q \ge -1, \qquad \alpha >-1. \end{aligned}$$

Such Besov spaces include bounded measurable functions, $L_1$-integrable functions, as well as Radon measures. To obtain such results, [2] establishes Lipschitz regularity for some related singular integrals using the stochastic sewing lemma introduced by the third co-author in [28]. The regularity threshold -1 is in agreement with the finite dimensional analogue [8] where it is shown that any SDE driven by additive fractional Brownian motion with Hurst parameter H∈(0,1) has a unique solution provided that the drift belongs to the Besov–Hölder space $\mathscr {B}_{\infty ,\infty }^\alpha$ with $\alpha > 1 - \frac{1}{2H}$. The two results are related by setting H=1/4, which is the temporal regularity of the random field solution of (1.2) with b=0. Quantitative convergence of discrete approximation schemes under the assumptions of [2] is also considered by Goudenége, Haress and Richard in [20], extending [6].

All of the aforementioned results concern the additive noise case. For the multiplicative case, much less is known. In [1, 3, 22], the authors show that (1.1) has a unique solution when σ is regular and bounded away from 0, the drift is measurable and bounded/locally bounded/integrable respectively. The proofs from these references rely on the Girsanov theorem, $L_p$-estimates for the density of the driftless equation (as obtained in [32]), and a comparison principle. Our result herein is an analogue of [2] for the multiplicative noise case. Namely, we show existence and uniqueness for (1.1) when σ is regular and bounded away from 0, the drift belongs to the Besov–Hölder space $\mathscr {B}_{\infty ,\infty }^\alpha$ with α>-1. In relation to the assumptions in [2]: notice that by Sobolev’s embedding theorem, we have $\mathscr {B}_{q,\infty }^\alpha \subset \mathscr {B}_{\infty , \infty }^{\alpha -1/q}$. Consequently, we also cover the cases $b \in \mathscr {B}_{q,\infty }^\alpha$ with $q \in [1, \infty ]$ and α-1/q>-1. Hence, the only case that we do not cover from [2] is the case α-1/q=-1 with α>-1 (see (1.3) above). In this sense, our assumption is slightly stronger than the one from [2], but at the same time our conclusion is also stronger: not only we show well-posedness but in fact we obtain Lipschitz stability of the solution map with respect to *b*. This lays the groundwork for a follow-up study on the numerical approximation of the equation, where schemes based on smoothed approximations of the drift will be employed. Such estimates are not expected to be true in the borderline case α-1/q=-1. In fact, the result in [2] is not quantitative but relies on an argument of Yamada-Watanabe kind. Concerning purely the well-posedness of the equation, one could combine the critical sewing methods from [2] with the techniques developed in the present article to tackle the case $\alpha -1/q =-1, \alpha >-1$ too. We have chosen not to pursue this route, as it would likely overwhelm the reader. Instead, we have opted to focus on the difficulties arising from the multiplicative noise and to highlight the essential elements of our approach. Similar to [2], our method also relies on the stochastic sewing lemma from [28] which does not rely on Girsanov theorem nor comparison principles. Therefore, the techniques within could also be applied to equations driven by Lévy noise and to systems of equations. Compared with [2], while the probabilistic properties of the noise term in the additive case are explicitly understood, this is no longer the case for our multiplicative equation (1.1). Therefore, employing the sewing methods in the present paper is more involved than [2]. In the sewing arguments in previous works, one approximates a solution using the integral form of the corresponding equation. This works quite well in the additive noise case, [2, 8]. It also works quite well in some multiplicative noise cases if the noise is not too irregular, for example equations driven by fractional Brownian motion with Hurst parameter H>1/2 [14]. However, for H<1/2, this approach leads to suboptimal results. The same is true for the setting of the present paper. With such an approach, one would only be able to obtain well-posedness when *b* has positive Hölder regularity. Instead, in order to cover the whole regime $b \in \mathscr {B}^\alpha _{\infty , \infty }$ with α>-1, we come up with sewing arguments that employ the flow of the driftless equation (see Section 3.2 for a more detailed overview of our method). Consequently, we need (and obtain) some regularisation estimates related to the density of the solution to the driftless equation and its derivatives. These estimates are achieved via Malliavin calculus which demands a relatively high regularity from σ. This approach is not equation-specific but rather works as a general principle. In fact, we are using it in a parallel work ([16]) to improve the results from [14] to optimal, in the case of rough equations driven by fractional Brownian motion with Hurst parameter H∈(1/3,1/2) .

The paper is organised as follows: In Section 2, we introduce some necessary notation. In Section 3, we define the key concepts, state the main result, and give an overview of the proof. Facts on stochastic sewing and Malliavin calculus are summarised in Section 4. In Section 5, we study the regularity of the flow of the driftless equation, provide bounds on the moments of its Malliavin derivatives, and establish relevant nondegeneracy estimates. In Section 6, we quantify how well the flow of the driftless equation approximates the solution of (1.1). In Section 7, we use the tools developed up until then to derive regularisation results for the solution of (1.1) via the stochastic sewing lemma. These estimates are then used in Section 8 to prove a stability result, and in Section 9 to estimate the drift term of (1.1). The main result is proven in Section 10. Finally, the appendix contains some useful auxiliary elementary estimates which are used throughout the article.

## Notation

Let $H := L_2([0,1]\times \mathbb {T})$. Let $\xi :=\{\xi (h): h \in H\}$ be an isonormal Gaussian process^1^ on a complete probability space $(\Omega ,\mathscr {F},\mathbb {P})$, and suppose that $\mathscr {F}$ is generated by ξ. Let $(\mathscr {F}_t)_{t \in [0,1]}$ be the filtration generated by ξ and augmented by the σ-algebra $\mathcal {N}$ generated by all $\mathbb {P}$-null sets, that is

$$\begin{aligned} \mathscr {F}_t := \sigma \big (\big \{\xi (\textbf{1}_{[0,r)\times B}) : r \in [0,t], B \in \mathscr {B}(\mathbb {T})\big \}\big )\vee \mathcal {N} \end{aligned}$$

where for two σ-algebras $\mathscr {X},\mathscr {Y}$ we denote $\mathscr {X} \vee \mathscr {Y} := \sigma (\mathscr {X} \cup \mathscr {Y})$. The predictable σ-algebra on $\Omega \times [0,1]$ is denoted by $\mathscr {P}$. The conditional expectation given $\mathscr {F}_t$ is denoted by $\mathbb {E}^t := \mathbb {E}(\cdot | \mathscr {F}_t)$. We use $L_p$ as a shorthand for $L_p(\Omega )$. For a sub-σ-algebra $\mathscr {G}\subset \mathscr {F}$, the conditional $L_p$-norm is denoted by

$$\Vert \cdot \Vert _{L_p|\mathscr {G}} := (\mathbb {E}(|\cdot |^p\vert \mathscr {G}))^{1/p},$$

and for $p \in [1,\infty )$, $q \in [1,\infty ]$ we denote

2.1 $$\begin{aligned} \Vert \cdot \Vert _{L_{p,q}^\mathscr {G}} := \Vert \Vert \cdot \Vert _{L_p|\mathscr {G}}\Vert _{L_q}. \end{aligned}$$

For a $\mathcal {P}\otimes \mathcal {B}(\mathbb {T})$-measurable map $f : \Omega \times [0,1] \times \mathbb {T} \rightarrow \mathbb {R}$ which satisfies $\mathbb {E} \Vert f\Vert _{L_2([0,1] \times \mathbb {T})}^2<\infty$, we denote its stochastic integral, in the sense of Walsh ([35]), with respect to the white noise ξ by

$$\begin{aligned} \int _0^t \int _{\mathbb {T}} f(s,y) \xi (dy, ds), \qquad t \in [0,1]. \end{aligned}$$

Similarly, if $(V, \langle \cdot ,\cdot \rangle _V)$ is a separable Hilbert space, for a $\mathcal {P}\otimes \mathcal {B}(\mathbb {T})$-measurable map $F: \Omega \times [0,1] \times \mathbb {T} \rightarrow {V}$ which satisfies $\mathbb {E} \Vert F\Vert _{L_2([0,1] \times \mathbb {T}; V)}^2<\infty$, one can define the *V*-valued stochastic integral

$$\begin{aligned} \int _0^t \int _{\mathbb {T}} F(s,y) \xi (dy, ds):= \sum _{k=1}^\infty v_k \int _{\mathbb {T}} \langle F(s,y), v_k \rangle _V \xi (dy, ds), \qquad t \in [0,1], \end{aligned}$$

where $(v_k)_{k=1}^\infty$ is an orthonormal basis of *V*. The above is a *V*-valued martingale with Doob–Meyer process

$$\begin{aligned} \Big \langle \int _0^\cdot \int _{\mathbb {T}} F(s,y) \xi (dy, ds) \Big \rangle _t = \int _0^t \int _{\mathbb {T}} \Vert F(s,y)\Vert _V^2 \, dy ds. \end{aligned}$$

In particular, the Burkholder-Davis-Gundy inequality holds (see, e.g., [30]): for any p≥1, there exists C=C(p), such that for any T∈[0,1]

2.2 $$\begin{aligned} \mathbb {E} \sup _{t \in [0, T]}\Big \Vert \int _0^t \int _{\mathbb {T}} F(s,y) \xi (dy, ds) \Big \Vert _V^p \le C \, \mathbb {E} \Big ( \int _0^T\int _{\mathbb {T}} \Vert F(s,y)\Vert _V^2 \, dy ds\Big )^{p/2}. \end{aligned}$$

From now on, we will refer to (2.2) as the “BDG inequality" for brevity.

Let $A\subseteq \mathbb {R}^d$ and (B,|·|) be a normed space. We denote by $\mathbb {B}(A,B)$ the collection of measurable functions f:A→B such that

$$\Vert f\Vert _{\mathbb {B}(A,B)} := \sup _{x \in A}|f(x)| <\infty .$$

We denote the space of bounded continuous functions f:A→B by *C*(*A*, *B*), and it is also canonically equipped with the $\mathbb {B}$-norm. For $\alpha \in \mathbb {N}$ we denote by $C^\alpha (A,B)$ the space of bounded continuous functions f:A→B such that for all multi-indices $l \in (\mathbb {Z}_{\ge 0})^d$ with $|l| \le \alpha$ the derivative $\partial ^l f$ is continuous, and

$$\begin{aligned}\Vert f\Vert _{C^\alpha (A,B)} := \sum _{|l| \le \alpha }\Vert \partial ^l f\Vert _{\mathbb {B}} <\infty . \end{aligned}$$

By convention the above sum includes the term $\Vert \partial ^{(0,\dots ,0)}f\Vert _{\mathbb {B}}$, where we define $\partial ^{(0,\dots ,0)}f:=f$. For $\alpha \in (0,1)$ and f:A→B, the α-Hölder seminorm of *f* is given by

$${[}f]_{C^\alpha (A,B)} : = \sup _{\begin{array}{c} x,y \in A\\ x\ne y \end{array}} \frac{|f(x) - f(y)|}{|x-y|^\alpha }.$$

For $\alpha \in (1, \infty ) \setminus \mathbb {Z}$ we then denote by $C^\alpha (A,B)$ the space of all functions such that for all multi-indices $l \in (\mathbb {Z}_{\ge 0})^d$ with |l|<α, the derivative $\partial ^l f$ exists, and

$$\begin{aligned} \Vert f\Vert _{C^\alpha (A,B)} := \Vert f\Vert _{C^{\lfloor \alpha \rfloor }(A,B)} + \sum _{\alpha -1\le |l|<\alpha } [\partial ^l f]_{C^{\alpha - |l|}(A,B)} <\infty . \end{aligned}$$

The collection of smooth (i.e. infinitely differentiable) and bounded functions with bounded derivatives will be denoted by

$$C^\infty (A,B) := \bigcap _{n =0}^{\infty } C^n(A,B).$$

For α<0 we say that a Schwartz-distribution *f* is of class $C^\alpha (\mathbb {R}, \mathbb {R})$, if

$$\begin{aligned} \Vert f\Vert _{C^\alpha (\mathbb {R},\mathbb {R})} : = \sup _{\varepsilon \in (0,1]} \varepsilon ^{-\alpha /2}\Vert P_\varepsilon ^{\mathbb {R}} f \Vert _{\mathbb {B}(\mathbb {R}, \mathbb {R})} <\infty , \end{aligned}$$

where for $(t,x) \in [0,1]\times \mathbb {R}$, $P_t^{\mathbb {R}} f(x) := \int _{\mathbb {R}}p_t^{\mathbb {R}}(x-y) f(y) dy$ and $p^\mathbb {R}$ is the *heat kernel* on $\mathbb {R}$ defined by

2.3 $$\begin{aligned} p_t^{\mathbb {R}}(x) := \frac{1}{(4\pi t)^{1/2}}\exp \Big (-\frac{|x|^2}{4t}\Big ). \end{aligned}$$

Note that for any $\alpha \in \mathbb {R}\setminus \mathbb {Z}$, the space $C^\alpha (\mathbb {R},\mathbb {R})$ coincides with the Besov space $\mathscr {B}_{\infty ,\infty }^\alpha$. We also define the *periodic heat kernel* on $\mathbb {T}$ for t∈[0,1] and $x,y \in \mathbb {T}$ by

2.4 $$\begin{aligned} p_t(x,y) := \sum _{k \in \mathbb {Z}}p_t^{\mathbb {R}}(x-y+k) \end{aligned}$$

and for $g:\mathbb {T} \rightarrow \mathbb {R}$ we denote $P_t g(x) := \int _{\mathbb {T}} p_t(x,y)g(y)dy$. When no ambiguity can arise, we will simply abbreviate $C^\alpha (A)$ or $C^\alpha$ for $C^\alpha (A,B)$. For α>-1 we denote the completion of $C^\infty$ in the norm $\Vert \cdot \Vert _{C^\alpha }$ by $C^{\alpha +}$.

### Remark 2.1

For all ε>0 we have the inclusions $C^{\alpha + \varepsilon } \subset C^{\alpha +} \subset C^{\alpha }$.

## Formulation and main result

### Formulation and main result

We introduce our main assumptions and the concept of solutions to (1.1).

#### Assumption 3.1

The function *b* is of class $C^{\alpha +}$ for some $\alpha \in (-1,0)$ and the function σ is of class $C^4$. Moreover, there exists a positive constant μ such that

$$\sigma (x) \ge \mu \quad \text {for all }x \in {\mathbb {R}}.$$

Finally, the initial condition $u_0 : \mathbb {T} \rightarrow \mathbb {R}$ is a bounded and continuous deterministic function.

Due to continuity, the above inequality is equivalent to the usual uniform ellipticity condition that $\sigma ^2 (x) \ge \mu ^2$ for all $X\in {\mathbb {R}}$.

#### Definition 3.2

*(Regularised solution)* Let $u: \Omega \times [0,1]\times \mathbb {T} \rightarrow \mathbb {R}$ be a $\mathscr {P}\otimes \mathscr {B}(\mathbb {T})$-measurable random field, such that *u*(*t*, *x*) is continuous in $(t,x) \in [0,1]\times \mathbb {T}$. We say that *u* is a *regularised solution* of (1.1) if there exists a $\mathscr {P}\otimes \mathscr {B}(\mathbb {T})$-measurable random field $D^{u}:\Omega \times [0,1]\times \mathbb {T} \rightarrow \mathbb {R}$ such that

1. For any sequence $(b^n)_{n\in \mathbb {N}} \subset C^\infty$ such that $b^n \rightarrow b$ in $C^{\alpha }$, we have that
  3.1 $$\begin{aligned} \sup _{(t,x) \in [0,1]\times \mathbb {T}}\Big |D_t^u(x) -\int _0^t \int _{\mathbb {T}}p_{t-r}(x,y)b^n(u(r,y))dydr\Big | \longrightarrow 0\end{aligned}$$
  in probability.
2. For each $$(t,x) \in [0,1]\times \mathbb {T}$$,
  3.2 $$\begin{aligned} u(t,x) = P_tu_0(x) + D_t^u(x) + \int _0^t \int _{\mathbb {T}} p_{t-r}(x,y) \sigma (u(r,y)) \xi (dy,dr)\qquad \text {a.s.} \end{aligned}$$

#### Remark 3.3

For a given regularised solution *u*, the random field $D^u$ is uniquely characterised by relation (3.1). Furthermore, in the more regular setting when $\alpha \ge 0$, Definition 3.2 reduces to the standard notion of a mild solution. In such case, one has $D^u_t(x)=\int _0^t \int _{\mathbb {T}}p_{t-r}(x,y)b(u(r,y))dydr$.

For $(S,T) \in [0,1]^2$ such that S≤T, let us define the simplices

$$\begin{aligned}\,[S,T]_{\le }^2 := \{ (s,t) \in [S,T]^2: s \le t\}\, \text { and } \,[S,T]_{<}^2 := \{(s,t) \in [S,T]^2 : s<t\}. \end{aligned}$$

To describe the regularity of the solutions, we introduce the following spaces of random fields.

#### Definition 3.4

*(The spaces *$\mathscr {V}_p^\beta$, $\mathscr {U}_p^\beta$*and *$\mathscr {U}^\beta$*)* Let $\beta \in [0, 1]$ and $p \in [1,\infty )$. We denote by $\mathscr {V}_p^{\beta }[0,1]$ the collection of all $\mathscr {P}\otimes \mathscr {B}(\mathbb {T})$-measurable functions $f:\Omega \times [0,1]\times \mathbb {T}\rightarrow \mathbb {R}$ such that $f \in \mathbb {B}([0,1]\times \mathbb {T}, L_p)$ and

$${[}f]_{\mathscr {V}_p^{\beta }[0,1]} := \sup _{x \in \mathbb {T}}\sup _{(s,t) \in [0,1]_{<}^2}\frac{\Vert f_t(x) - P_{t-s}f_s(x)\Vert _{L_{p,\infty }^{\mathscr {F}_s}}}{|t-s|^{\beta }} < \infty . $$

For $(S,T) \in [0,1]^2_{\le }$, the space $\mathscr {V}_p^{\beta }[S,T]$ and the corresponding seminorm are defined analogously. We denote by $\mathscr {U}_p^\beta$ the collection of all regularised solutions *u* of (1.1) such that $D^u \in \mathscr {V}_p^{\beta }[0,1]$. We moreover define

$$\mathscr {U}^\beta := \bigcap _{p=1}^\infty \mathscr {U}_p^\beta .$$

We are now in position to state our main theorem.

#### Theorem 3.5

(Well-posedness) Let Assumption 3.1 hold. There exists a regularised solution *u* to (1.1) in the class $\mathscr {U}^{1 + \alpha /4}$. Moreover if *v* is another solution of (1.1) in the class $\mathscr {U}_2^\beta$ for some $\beta \ge \frac{1}{2}- \frac{\alpha }{4}$, then u(t,x)=v(t,x) almost surely for all $(t,x) \in [0,1]\times \mathbb {T}$.

### Overview of methods of proofs

The bulk of the proofs relies on moment estimates for singular integrals which are typically of the form

$$\begin{aligned} I:=\int _0^1\int _{\mathbb {T}}h(y)f(u(r,y))dydr \end{aligned}$$

where *h* is an integrable function, *u* is a solution to (1.1) and *f* is a distribution with negative Hölder regularity. An effective tool to estimate moments of *I*, which emerges from [28], is the stochastic sewing lemma. Heuristically, the lemma decomposes *I* corresponding to partitions of the time interval [0, 1] with vanishing mesh size. More precisely, let π be a partition of [0, 1], then one writes

$$\begin{aligned} I=\sum _{[s,t]\in \pi }\int _s^t\int _{\mathbb {T}}h(y)f(u(r,y))dydr. \end{aligned}$$

On each subinterval [*s*, *t*], we approximate the random variable $\int _s^t\int _{\mathbb {T}}(\ldots )dydr$ by its conditional expectation given $\mathscr {F}_s$, i.e. $\mathbb {E}^s\int _s^t\int _{\mathbb {T}}(\ldots )dydr$. Because the conditional law of *u*(*r*, *y*) given $\mathscr {F}_s$ is unknown a priori, we further approximate *u*(*r*, *y*) by a random variable, denoted by $\psi ^s(r,y)$. There are two desirable properties for these approximations. First, one must recover *I* when the mesh size of π vanishes, namely

$$\begin{aligned} I=\lim _{|\pi |\downarrow 0} \sum _{[s,t]\in \pi }\mathbb {E}^s\int _s^t\int _{\mathbb {T}}h(y)f(\psi ^s(r,y))dydr. \end{aligned}$$

Second, the conditional expectation $\mathbb {E}^s f(\psi ^s(r,y))$ is well-defined and can be estimated so that for some p≥2 and ε>0, one has

3.3 $$\begin{aligned} \Vert \mathbb {E}^s\int _s^t\int _{\mathbb {T}}h(y)f(\psi ^s(r,y))dydr\Vert _{L_p(\Omega )}\lesssim (t-s)^{\frac{1}{2}+\varepsilon } \end{aligned}$$

and

3.4 $$\begin{aligned} \Vert \mathbb {E}^s\int _a^t\int _{\mathbb {T}}h(y)[f(\psi ^s(r,y))-f(\psi ^a(r,y)]dydr\Vert _{L_p(\Omega )}\lesssim (t-s)^{1+\varepsilon } \end{aligned}$$

for every $s\le a\le t$. Under these two properties, the stochastic sewing lemma can be applied, and it provides estimates for the *p*-th moment of *I*.

Let us explain how $\psi ^s$ is chosen. Relation (3.2) provides a natural decomposition of a solution as the sum of a nondegenerate noise and the drift, namely

$$\begin{aligned} u(t,x)&=P_tu_0(x)+D^u_t(x)+V_t(x), \text { where }\\ V_t(x)&=\int _0^t \int _{\mathbb {T}} p_{t-r}(x,y) \sigma (u(r,y)) \xi (dy,dr). \end{aligned}$$

It follows that for each s≤t,

$$\begin{aligned} u(t,x)=P_{t-s}u(s,\cdot )(x)+ [D^u_t(x)-P_{t-s}D^u_s(x)]+[V_t(x)-P_{t-s}V_s(x)]. \end{aligned}$$

One could then choose to approximate *u*(*t*, *x*) by the random variable

$$\begin{aligned} \psi ^s(t,x)&:=P_{t-s}u(s,\cdot )(x)+[V_t(x)-P_{t-s}V_s(x)]. \end{aligned}$$

The error of this approximation can be quantified by the following estimate

3.5 $$\begin{aligned} \Vert u(t,x)- \psi ^s(t,x)\Vert _{L_p(\Omega )} \lesssim |t-s|^\gamma \end{aligned}$$

for every s≤t and for some γ>0. The larger the value of γ is, the better the approximation is. We note that

$$\begin{aligned} [V_t(x)-P_{t-s}V_s(x)] =\int _s^t \int _{\mathbb {T}} p_{t-r}(x,y) \sigma (u(r,y)) \xi (dy,dr). \end{aligned}$$

In the additive case (i.e. when σ is a constant), $V_t(x)-P_{t-s}V_s(x)$ has a normal distribution and hence, the conditional expectation $\mathbb {E}^s f(\psi ^s(t,x))$ can be evaluated precisely. The stochastic sewing method described above can be applied (i.e. achieving (3.3) and (3.4)) under some suitable regularity assumptions on *f* and that $\gamma >1/2-\alpha /4 \approx 3/4$ for $\alpha \approx -1$ (which is the worst regularity index imposed on the drift).

This is the approach from [2].

Going toward the multiplicative noise case, one might hope that a similar argument would work. Notice that in this case, the distribution of $V_t(x)-P_{t-s}V_s(x)$ conditionally on $\mathscr {F}_s$ is not known a priori. A naive way to circumvent this issue is to consider

$$\begin{aligned} \psi ^s(t,x)&:=P_{t-s}u(s,\cdot )(x)+\int _s^t \int _{\mathbb {T}} p_{t-r}(x,y) \sigma (u(s,y)) \xi (dy,dr), \end{aligned}$$

which is obtained by freezing the solution in the integrand at time *s*. In this way, conditionally on $\mathscr {F}_s$, $\psi ^s(t,x)$ once again has a normal distribution, which allows for concrete analysis. However, one can not go far with this choice as it is immediate that

3.6 $$\begin{aligned} u(t,x)- \psi ^s(t,x) =\,&\int _s^t \int _{\mathbb {T}} p_{t-r}(x,y) \big ( \sigma (u(r,y))- \sigma (u(s,y)) \big )\xi (dy,dr)\nonumber \\&{+[D^u_t(x)-P_{t-s}D^u_s(x)] }. \end{aligned}$$

The moments of the stochastic integral on the right-hand side above are (expectedly) of order $$ |t-s|^{1/2}$$ (consisting of two contributions of the same order 1/4 from the stochastic integral and from the temporal regularity of the solution). The exponent 1/2 falls short of the required threshold 3/4 which is necessary in the additive case. This makes the naive approximation (3.6) unsuitable for the sewing method under Assumption 3.1.

Moving forward, to resolve these issues, we introduce the following approximation

3.7 $$\begin{aligned} \psi ^s(t,x):= \phi ^{u(s,\cdot ),s}(t,x), \end{aligned}$$

where $\phi ^{z,s}$ denotes the solution to the *driftless equation*

$$\begin{aligned} (\partial _t - \Delta )\phi ^{z,s} = \sigma (\phi ^{z,s})\xi , \quad \phi ^{z,s}(s,\cdot )=z(\cdot ). \end{aligned}$$

Observe that when σ is a constant, (3.6) and (3.7) coincide, but otherwise they are generally different. Indeed, we show in Section 6 that the approximation (3.7) satisfies the estimate (3.5) with $\gamma = 1+\alpha /4$ which is larger than 1/2-α/4 as is required for the application of the sewing method. The distribution of $\psi ^s(t,x)$ conditioned on $\mathscr {F}_s$ might not be as explicit as in the additive noise case but nevertheless, one can extract the information which is sufficient to verify (3.3) and (3.4). This essentially boils down to obtaining estimates related to the density of the solution of the driftless equation and its derivatives, which are achieved by tools from Malliavin calculus (see Section 5.3).

When comparing our method to the existing ones from the literature, we can draw some similarities as well as genuine differences. The works [1, 3, 22] also utilise estimates on the density of the solution to the driftless equation, however, in a completely different way. In fact, these works use Girsanov theorem to extract relevant and useful a priori estimates for the solution to (1.1) from the solution of the driftless equation. Under our main assumption, the Girsanov theorem is not applicable which makes this argument obsolete. Additionally, our uniqueness argument relies on qualitative stability estimates, as opposed to comparison principles in the aforementioned works. Compare to [2], we also use stochastic sewing method. However, while [2] relies on the approximation (3.6), we introduce and utilise the better approximation (3.7). To the best of our knowledge, this is the first time it has been used in the study of regularisation by noise phenomena by sewing methods. Furthermore, because the conditional law of $\psi ^s$ is not explicit, additional works have been carried out in order to apply the sewing method successfully.

The driftless equation also appears in [9] in the study of regularisation by multiplicative fractional noise for SDEs. In this work, the authors employ a transformation, which is based on the inverse of the flow generated by the driftless equation, to transform the original equation into an additive one. Comparing the results of [9] and [14] reveals that such transformation is quite demanding and does not lead to results which are in alignment with [8]. The connection between (1.1) and the driftless equation is well-known, perhaps since the Girsanov theorem. Another instance of such relation appears in [26] in a different context. Our work therefore exhibits a new connection between the two equations.

## Preliminaries

We state and recall some facts about stochastic sewing and Malliavin calculus. When a result is known, we refer to the cited references for proofs. Other statements are relatively straightforward and short proofs are given.

### Stochastic sewing

Let $(S,T) \in [0,1]_{<}^2$. For any functions $\mathscr {A}:[S,T] \rightarrow \mathbb {R}$, $A:[S,T]^2_\le \rightarrow \mathbb {R}$, for any $(s,t) \in [0,1]_{\le }^2$ and a∈[s,t], we define $\mathscr {A}_{s,t} := \mathscr {A}_t - \mathscr {A}_s$, and $\delta A_{s,a,t} := A_{s,t} - A_{s,a} - A_{a,t}$.

The first stochastic sewing lemma is introduced by the third co-author in [28]. To best suit our purpose herein, we state a conditional version of the lemma which applies in settings with $L_{q,p}^{\mathscr {F}_s}$-norms (defined in (2.1)). This version is originated from the works [2, 19, 29], where the reader can find its proof.

#### Lemma 4.1

(Conditional stochastic sewing lemma) Let *p*, *q* satisfy $2 \le q \le p \le \infty$ with q<∞. Let $(S,T) \in [0,1]_{\le }^2$ and let $A:[S,T]_{\le }^2 \rightarrow L_p(\Omega )$ be a function such that for any $(s,t) \in [S,T]_{\le }^2$ the random vector $A_{s,t}$ is $\mathscr {F}_t$-measurable. Suppose that for some $\varepsilon _1, \varepsilon _2>0$ and $C_1,C_2$ the bounds

4.1 $$\begin{aligned} \Vert A_{s,t}\Vert _{L_{q,p}^{\mathscr {F}_s}} \le C_1|t-s|^{1/2+\varepsilon _1}, \quad \Vert \mathbb {E}^s \delta A_{s,a,t}\Vert _{L_p} \le C_2|t-s|^{1+\varepsilon _2} \end{aligned}$$

hold for all $S \le s \le a \le t \le T$. Then, there exists a unique map $\mathscr {A}:[S,T] \rightarrow L_p(\Omega )$ such that $\mathscr {A}_S =0$, $\mathscr {A}_t$ is $\mathscr {F}_t$-measurable for all t∈[S,T], and the following bounds hold for some constants $K_1,K_2>0$:

4.2 $$\begin{aligned} \Vert \mathscr {A}_{s,t} - A_{s,t}\Vert _{L_{q,p}^{\mathscr {F}_s}}&\le K_1 |t-s|^{1/2 + \varepsilon _1} \end{aligned}$$

4.3 $$\begin{aligned} \Vert \mathbb {E}^s(\mathscr {A}_{s,t} - A_{s,t})\Vert _{L_p}&\le K_2 |t-s|^{1+\varepsilon _2}. \end{aligned}$$

Furthermore, there exists a constant *K* depending only on $\varepsilon _1,\varepsilon _2, d,p$ such that $\mathscr {A}$ satisfies the bound

4.4 $$\begin{aligned} \Vert \mathscr {A}_{s,t}\Vert _{L_{q,p}^{\mathscr {G}_s}} \le K C_1 |t-s|^{1/2 + \varepsilon _1} + K C_2 |t-s|^{1+\varepsilon _2} \end{aligned}$$

for all $$(s,t)\in [S,T]_{\le }^2$$.

We will call *A* *a germ of* the process $\mathscr {A}$. In practice, we mostly take q=p (in which case, $L_{q,p}^{\mathscr {F}_s}$-norm and $L_{p}$-norm coincide) and p=∞.

### Malliavin calculus

Let $\mathscr {W}$ denote the the space of *smooth and cylindrical random variables*, i.e. random variables of the form

$$F =f(\xi (h_1),\dots , \xi (h_n))$$

for some $n \in \mathbb {N}$, $h_1,\dots , h_n \in H$, and for some smooth *f* such that *f* and its partial derivatives of all orders have polynomial growth. The *Malliavin derivative* of such a random variable is given by

$$\mathscr {D}_{\theta ,\zeta }F:= \sum _{i=1}^n \partial _{i}f(\xi (h_1),\dots , \xi (h_n)) h_i(\theta ,\zeta )$$

for all $(\theta ,\zeta ) \in [0,1]\times \mathbb {T}$ where $\partial _i$ denotes partial derivative with respect to the *i*-th argument. For all $k \in \mathbb {Z}_{\ge 0}$, p≥1 the iterated Malliavin derivative $\mathscr {D}^k$ is closable as an operator from $L_p(\Omega )$ into $L_p(\Omega ; H^{\otimes k})$. By convention, the 0-th Malliavin derivative is the identity map, and $H^{\otimes 0} := \mathbb {R}$. For $k \in \mathbb {Z}_{\ge 0}$ and p≥1, we denote by $\mathscr {W}^k_p$ the completion of $\mathscr {W}$ with respect to the norm

$$F \mapsto \Vert F\Vert _{\mathscr {W}^k_p} :=\Big ( \mathbb {E}|F|^p + \sum _{i=1}^k \mathbb {E}\Vert \mathscr {D}^iF\Vert _{H^{\otimes i}}^p \Big )^{1/p}.$$

We moreover use the notation

$$\mathscr {W}^k := \bigcap _{p \ge 1} \mathscr {W}_p^k.$$

On the class $\mathscr {W}_p^k$ one can also define the -seminorm byBy convention, we haveNote that $\Vert \cdot \Vert _{\mathscr {W}_p^k}$ and  are equivalent norms. The above definitions can be extended for the Hilbert-space valued case as follows. Let *V* be a separable Hilbert-space, and consider the family $\mathscr {W}(V)$ of random variables of the form

$$F = \sum _{i=1}^m F_i v_i$$

for some $F_1,\dots , F_m \in \mathscr {W}$, and $v_1,\dots , v_m \in V$. For k≥1, we define

$$\mathscr {D}^k F := \sum _{j=1}^m \mathscr {D}^kF_j \otimes v_j.$$

Then $\mathscr {D}^k$ is a closable operator from $L_p(\Omega ;V)$ into $L_p(\Omega ; H^{\otimes k}\otimes V)$ for any p≥1. We define the space $\mathscr {W}^k_p(V)$ as the completion $\mathscr {W}(V)$ with respect to the norm

$$F \mapsto \Vert F\Vert _{\mathscr {W}^k_p(V)} :=\Big ( \mathbb {E}\Vert F\Vert _V^p + \sum _{i=1}^k \mathbb {E}\Vert \mathscr {D}^iF\Vert _{H^{\otimes i}\otimes V}^p \Big )^{1/p}.$$

For a random variable $u \in L_2(\Omega ;H)$ it is said that $u \in \text {dom}(\delta )$, if there exists a constant c>0 such that

$$\mathbb {E}\langle \mathscr {D}F, u \rangle _H \le c\Vert F\Vert _{L_2}$$

for all $F \in \mathscr {W}^1_2$. If this holds, then δ(u) denotes the unique element of $L_2(\Omega )$ that satisfies

$$\begin{aligned}\mathbb {E}(F\delta (u)) = \mathbb {E}\langle \mathscr {D}F , u \rangle _H \end{aligned}$$

for any $F \in \mathscr {W}^1_2$. The random variable δ(u) is called the *Skorokhod integral* (or the *divergence*) of *u*. If in addition *u* is adapted, then the Skorokhod integral coincides with the usual stochastic integral, that is for all t∈[0,1] we have

$$\int _0^t \int _{\mathbb {T}} u(r,y) \xi (dy,dr) = \delta (u \textbf{1}_{[0,t]}).$$

The following result follows from [31, Proposition 2.1.4].

#### Proposition 4.2

(Malliavin integration by parts) Let $n \in \mathbb {N}$, $u, G_0 \in \mathscr {W}^n$ and let $f:\mathbb {R} \rightarrow \mathbb {R}$ be *n* times differentiable. Suppose moreover that for all $p \in [1,\infty )$, we have $\mathbb {E}\Vert \mathscr {D}u\Vert _{H}^{-p} <\infty .$ Define iterated Skorokhod integrals recursively for $k \in \{0,\dots , n-1\}$ by

$$\begin{aligned} G_{k+1} := \delta \Big (\frac{\mathscr {D}u}{\Vert \mathscr {D}u\Vert _H^2}G_k\Big ). \end{aligned}$$

The following holds:

$$\begin{aligned} \mathbb {E}\big (\nabla ^n f(u) G_0\big ) = \mathbb {E}\big ( f(u) G_n\big ). \end{aligned}$$

We also recall the combinatorial notation from [10]. Let $n \in \mathbb {N}$.

- For $1 \le k \le n$, we denote by Λ(n,k) the set of partitions of the integer *n* of length *k*, that is, if $\lambda \in \Lambda (n,k)$, then $\lambda \in \mathbb {N}^k$, and by writing $\lambda = (\lambda _1 ,\dots , \lambda _k)$, it satisfies
  $$\lambda _1 \ge \dots \ge \lambda _k \ge 1 \qquad \text { and }\qquad \sum _{i=1}^k \lambda _i = n. $$
- For $\lambda \in \Lambda (n,k)$, we let $\mathcal {P}(n, \lambda )$ be all partitions of *n* ordered objects $\{\theta _1 ,\dots , \theta _n\}$, with $\theta _1 \ge \dots \ge \theta _n$ into *k* groups $\{\theta _1^1,\dots , \theta _{\lambda _1}^1\},\dots , \{\theta _1^k,\dots , \theta _{\lambda _k}^k\}$, such that within each group the elements are ordered, i.e. $\theta _1^j \ge \dots \ge \theta _{\lambda _j}^j$ for $1 \le j \le k$. Note that $| \mathcal {P}(n,\lambda )| = { n \atopwithdelims ()\lambda _1,\dots , \lambda _k} = \frac{n!}{\lambda _1!\dots \lambda _k!}.$
- For a generic element
  $$\begin{aligned} \gamma&:= ((\theta _1,\zeta _1),\dots ,(\theta _n,\zeta _n)) \in ([0,1]\times \mathbb {T})^n, \end{aligned}$$
  we will denote by $\hat{\gamma }_k$ the element of $([0,1]\times \mathbb {T})^{n-1}$ that is obtained by omitting the *k*-th entry of γ, i.e.
  4.5 $$\begin{aligned} \hat{\gamma }_k&:= ((\theta _1,\zeta _1),\dots , (\theta _{k-1},\zeta _{k-1}),(\theta _{k+1},\zeta _{k+1}),\dots , (\theta _n,\zeta _n)). \end{aligned}$$

We state some generic estimates on the Malliavin derivatives of functions of random variables which are needed in later sections. The proofs of these results rely purely on elementary principles, such as the chain rule.

#### Proposition 4.3

([10, Lemma 5.3]) Suppose that $f \in C^n$ and $\phi \in \mathscr {W}^n$. Then, for almost all $\gamma = ((\theta _1,\zeta _1), \dots , (\theta _n, \zeta _n)) \in ([0,1]\times \mathbb {T})^n$, we have

4.6 $$\begin{aligned} \mathscr {D}_\gamma ^n f(\phi ) = \sum _{k=1}^{n} \nabla ^kf(\phi ) \sum _{\lambda \in \Lambda (n,k)} \sum _{\mathcal {P}(n,\lambda )} \prod _{j=1}^k \mathscr {D}^{\lambda _j}_{(\theta _1^j, \zeta _1^j),\dots , (\theta _{\lambda _j}^j, \zeta _{\lambda _j}^j) }\phi . \end{aligned}$$

#### Lemma 4.4

Fix some constants ε>0 and $n \in \mathbb {N}$. For $i \in \{1,\dots , 4\}$ consider random variables $\phi ^i \in \mathscr {W}^n$. Suppose that for all $p \in [1,\infty )$ and positive integer k≤n-1 there exists a constant $N_0 = N_0(k,p)$ such that4.7Suppose that $f: \mathbb {R} \rightarrow \mathbb {R}$ is smooth. For all $p \in [1,\infty )$ the following statements hold.

- There exists a constant $N = N(n,p,\Vert f\Vert _{C^n})>0$ such that
- There exists a constant $N = N(n,p, \Vert f\Vert _{C^{n+1}})$ such that 4.8
- Suppose moreover that (4.7) also holds for k=n. There exists a constant $N = N(n,p,\Vert f\Vert _{C^{n+2}})$ such that

#### Proof

By (4.6) we can see thatThe second term in this expression can be estimated using (4.7) by

$$\begin{aligned} \sum _{k=2}^n \sum _{\lambda \in \Lambda (n,k)} \sum _{\mathscr {P}(n,\lambda )}\prod _{j=1}^k \varepsilon ^{ \lambda _j}\lesssim \varepsilon ^{n} \end{aligned}$$

where we used the definition of Λ(n,k). This proves point (a).

We proceed by proving point (b). Note that by the Minkowski inequality and the Leibniz rule, by (4.7), and by point (a) we getFrom here point (b) follows.

Finally, we prove point (c). By Lemma 11.7 we have thatwhere for each $\theta ,\eta \in [0,1]$, the expressions $\Theta _1(\theta ,\eta ), \Theta _2(\theta )$ are convex combinations of $\phi ^1,\dots ,\phi ^4$. By the Minkowski inequality and by the Hölder inequality, we getBy using point (a) for k≥1, and using the regularity of *f* for k=0, we can see that  Therefore we getFinally,where the last inequality again follows from point (a). Hence the proof is finished. □

#### Lemma 4.5

Consider constants ε,c>0, $n \in \mathbb {Z}_{\ge 0}$. Let $X \in \mathscr {W}^{n+1}$, $Y \in \mathscr {W}^n$ with Y≥0. Suppose that for all $p \in (2,\infty )$ there exists a constant $N_0 = N_0(p)>0$ such that for all $k \in \{1,\dots , n+1\}$, $l \in \{0,\dots , n\}$ we have4.9Define an *H*-valued random variable by $w := \frac{\mathscr {D}X}{Y}$. Then, for each $p \in [1,\infty )$, there exists a constant $N = N(N_0,n,p)>0$ such that

$$\Vert \mathscr {D}^n w \Vert _{L_p (\Omega , H^{\otimes (n+1)})} \le N \varepsilon ^{n+1 -c}.$$

#### Proof

We may assume that p>2. By (4.6) and a simple approximation argument (shifting *Y* away from 0) we have that $Y \in \mathscr {W}^n$, and for $m \in \{1,\dots , n\}$, $\gamma \in ([0,1]\times \mathbb {T})^m$ we have

$$\begin{aligned} \mathscr {D}^m_\gamma (Y)^{-1} = \sum _{k=1}^m \frac{(-1)^k k!}{(Y )^{k+1}}\sum _{\lambda \in \Lambda (n,k)}\sum _{\mathcal {P}(n, \lambda )}\prod _{j=1}^k \mathscr {D}^{\lambda _j}_{(\theta _1^j,\zeta _1^j),\dots (\theta _{\lambda _j}^j,\zeta _{\lambda _j}^j)}Y. \end{aligned}$$

Thus by (4.9) we have

4.10 $$\begin{aligned} \Vert \mathscr {D}^m (Y^{-1})\Vert _{L_p(\Omega ; H^{\otimes m})}&\lesssim \sum _{k=1}^m \varepsilon ^{-c(k+1)} \sum _{\lambda \in \Lambda (m,k)}\sum _{\mathcal {P}(m,\lambda )}\prod _{j=1}^k \varepsilon ^{c + \lambda _j} \nonumber \\&\lesssim \sum _{k=1}^m \varepsilon ^{-c(k+1)} \sum _{\lambda \in \Lambda (m,k)}\sum _{\mathcal {P}(m,\lambda )} \varepsilon ^{\sum _{j=1}^k(c+\lambda _j)}\nonumber \\&\lesssim \sum _{k=1}^m\varepsilon ^{-c(k+1) + ck + m} \lesssim \varepsilon ^{m-c}. \end{aligned}$$

For $\gamma \in ([0,1]\times \mathbb {T})^n$ and for $\eta \in [0,1]\times \mathbb {T}$, using the Leibniz rule, we have

4.11 $$\begin{aligned} \mathscr {D}_\gamma ^n w(\eta ) = \sum _{\lambda _1 + \lambda _2 =n}\sum _{(\gamma _1 , \gamma _2) \in \mathcal {P}(n,2)} \mathscr {D}_{\gamma _1}^{\lambda _1}(\mathscr {D}_\eta X) \mathscr {D}_{\gamma _2}^{\lambda _2}(Y^{-1}). \end{aligned}$$

Using (4.11), (4.9) and (4.10) we get that

$$\begin{aligned}&\Vert \Vert \mathscr {D}^n w \Vert _{H^{\otimes (n+1)}}\Vert _{L_p} \\&\quad \lesssim \sum _{ \lambda _1 + \lambda _2 =n}\sum _{(\gamma _1 , \gamma _2) \in \mathcal {P}(n,2)} \Vert \Vert \mathscr {D}^{\lambda _1 +1}X \Vert _{H^{\otimes (\lambda _1 +1)}}\Vert _{L_{2p}}\Vert \Vert \mathscr {D}^{\lambda _2}(Y^{-1})\Vert _{H^{\otimes \lambda _2}}\Vert _{L_{2p}}\\&\quad \lesssim \sum _{\lambda _1 + \lambda _2 =n}\sum _{(\gamma _1 , \gamma _2) \in \mathcal {P}(n,2)} \varepsilon ^{\lambda _1 +1}\varepsilon ^{\lambda _2 -c}\lesssim \varepsilon ^{n+1 -c} \end{aligned}$$

as required. □

## Malliavin regularity for the driftless equation

This section is concerned with the solution of the driftless multiplicative stochastic heat equation

5.1 $$\begin{aligned} (\partial _t - \Delta )\phi&= \sigma (\phi ) \xi , \qquad \phi (0,\cdot )&=\phi _0 \end{aligned}$$

and its Malliavin derivatives. Herein, $\phi _0 \in C(\mathbb {T})$ is fixed and the solution $\phi : \Omega \times [0,1]\times \mathbb {T} \rightarrow \mathbb {R}$ is a $\mathscr {P}\otimes \mathscr {B}(\mathbb {T})$-measurable random field, a.s. continuous on $[0,1]\times \mathbb {T}$, and satisfies the following equation almost surely

5.2 $$\begin{aligned} \phi (t,x) =P_t\phi _0(x)+ \int _0^t \int _{\mathbb {T}}p_{t-r}(x,y) \sigma (\phi (r,y))\xi (dy,dr), \quad \forall (t,x) \in [0,1]\times \mathbb {T}. \end{aligned}$$

### Moment bounds for Malliavin derivatives

We will show the following result.

#### Lemma 5.1

Let ϕ be the solution of (5.1). For any $n \in \mathbb {Z}_{\ge 0}$ and $p \in [1,\infty )$, if in addition $\sigma \in C^n$, then there exists some constant $N = N(n,p,\Vert \sigma \Vert _{C^n})$ such that for all t∈[0,1] we have

To show this, let us recall the following non-quantitative result from [4, Proposition 4.3].

#### Proposition 5.2

(Boundedness and Malliavin differentiability) Let ϕ be the solution of (5.1). For any $n \in \mathbb {N}$, $p \in [1,\infty )$, if $\sigma \in C^n$, then we have

$$\sup _{(t,x) \in [0,1]\times \mathbb {T}}\Vert \phi (t,x)\Vert _{{\mathscr {W}}_p^n} <\infty .$$

We will also need the following result from [10, Lemma 5.6]:

#### Proposition 5.3

Let ϕ be the solution of (5.1), and let $n \in \mathbb {N}, p \in [1,\infty ), \sigma \in C^n.$ For all $(t,x) \in [0,1]\times \mathbb {T}$ and for almost every $\gamma = (\theta _i,\zeta _i)_{i=1}^n \in ([0,1]\times \mathbb {T})^n,$ we have

5.3 $$\begin{aligned} \mathscr {D}_\gamma ^n \phi (t,x)&= \textbf{1}_{[0,t]}(\theta ^*)\sum _{k=1}^n p_{t-\theta _k}(x,\zeta _k) \mathscr {D}_{\hat{\gamma }_k}^{n-1}[\sigma (\phi (\theta _k,\zeta _k))] \nonumber \\&\qquad +\textbf{1}_{[0,t]}(\theta ^*)\int _{{\theta }^*}^t \int _{\mathbb {T}}p_{t-r}(x,y) \mathscr {D}_{\gamma }^n[\sigma (\phi (r,y))] \xi (dy,dr), \end{aligned}$$

where $\hat{\gamma }_k$ is defined by (4.5) and $\theta ^* := \max _{k \in \{1,\dots , n\}} \theta _k$.

#### Remark 5.4

Note that in the above equation the stochastic integral can be taken over the time interval [0, *t*] rather than $[\theta ^*,t]$, since for $r \le \theta ^*$ the Malliavin derivative $\mathscr {D}_\gamma ^n \sigma (\phi (r,y))$ is zero (see [33, Remark 5.1]).

We can now proceed with the proof of Lemma 5.1.

#### Proof of Lemma 5.1

We will prove the result by induction. By the BDG inequality and by the boundedness of σ, the result holds for the case n=0. Suppose that the result holds for the first (n-1) Malliavin derivatives. We aim to show that the result also holds for the *n*-th Malliavin derivative. Assume without loss of generality that p≥2. By (5.3) and the BDG inequality, we get5.4We proceed with proving that

5.5 $$\begin{aligned} A(t,x) \lesssim t^{n/2}. \end{aligned}$$

Indeed, in the n=1 case, we have by the boundedness of σ that

$$\begin{aligned} A(t,x) = \Vert p_{t-\cdot }(x,\cdot )\sigma (\phi (\cdot ,\cdot ))\textbf{1}_{[0,t]}(\cdot )\Vert _{L_p(\Omega , H)}^2 \lesssim \Vert p_{t-\cdot }(x,\cdot )\textbf{1}_{[0,t]}(\cdot )\Vert _{H}^2 \lesssim t^{1/2}. \end{aligned}$$

Moreover in the n≥2 case, by point (a) of Lemma 4.4 and by the induction hypothesis we have for $(\theta ,\zeta ) \in [0,t]\times \mathbb {T}$ thatand thus using Minkowski’s inequality and the above bound, we get thatas required. Hence (5.5) is proven. We now proceed by bounding *B*. By point (a) of Lemma 4.4 and by the induction hypothesis we have5.6By (5.4), and by our bounds (5.5), (5.6) on *A*, *B*, we conclude thatBy Proposition 5.2 we have that  Therefore by Lemma 11.4, the statement we aim to show also holds for the *n*-th Malliavin derivative. Thus the proof is finished. □

#### Lemma 5.5

Let $n \in \mathbb {Z}_{\ge 0}$, $p \in [1,\infty )$, $\sigma \in C^{n+1}$, and let ϕ solve (5.1). There exists some constant $N =N(n,p, \Vert \sigma \Vert _{C^{n+1}})$ such that for all t∈(0,1] we have

#### Proof

By using the Minkowski inequality, the Leibniz rule, Hölder’s inequality, and Lemma 5.1, we can see thatas required. □

### Lipschitz regularity with respect to the initial condition

For any $z \in C(\mathbb {T})$, let $\phi ^{z}$ denote the solution of (5.1) with $\phi _0=z$. For $n \in \mathbb {N}$, $\sigma \in C^n$, $q \in [1, \infty )$, $(z_1,z_2) \in (C(\mathbb {T}))^2$, $(t,x) \in [0,1]\times \mathbb {T}$, we define5.75.8The main result of this section is the following:

#### Lemma 5.6

Let $n \in \mathbb {Z}_{\ge 0}$ and assume that $\sigma \in C^{n+1}$. For all $q \in [2, \infty )$, $(t,x) \in [0,1]\times \mathbb {T}$, the map $F_{q,n}^{(2)}(t,x,\cdot , \cdot )$ is continuous on $C(\mathbb {T})\times C(\mathbb {T})$.

Moreover for all $q,p_1 \in [2, \infty )$ and $p_2 \in [2, \infty ]$ there exists a constant $N = N(n,p_1,p_2,q,\Vert \sigma \Vert _{C^{n+1}})$ such that for any σ-algebra $\mathcal {G} \subset \mathcal {F}$ and any random variables $Z,\bar{Z} \in L_p(\Omega , C(\mathbb {T}))$ and for all $(t,x) \in [0,1]\times \mathbb {T}$ we have

$$\big \Vert F_{q,n}^{(2)}(t,x,Z,\bar{Z})\big \Vert _{L^{\mathscr {G}}_{p_1,p_2}}\le N t^{n/4}\sup _{w \in \mathbb {T}}\Vert Z(w)-\bar{Z}(w)\Vert _{L_{p_1,p_2}^{\mathscr {G}}}.$$

#### Proof

We proceed by induction.

Step 1: We show that the statement holds for n=0. For $z,\bar{z} \in C(\mathbb {T})$, we have by (5.2) that

$$\begin{aligned} \phi ^z(t,x) - \phi ^{\bar{z}}(t,x)&= P_t(z-\bar{z})(x) + \int _0^t\int _{\mathbb {T}} p_{t-r}(x,y)(\sigma (\phi ^z(r,y)) \\&\qquad - \sigma (\phi ^{\bar{z}}(r,y))\xi (dy,dr). \end{aligned}$$

Therefore, by the BDG inequality we get

5.9 $$\begin{aligned}&\Vert \phi ^z(t,x) - \phi ^{\bar{z}}(t,x)\Vert _{L_q}^2 \nonumber \\&\qquad \lesssim |P_{t}(z-\bar{z})(x)|^2 + \int _0^t \int _{\mathbb {T}} p_{t-r}^2(x,y)\Vert \phi ^z(r,y) - \phi ^{\bar{z}}(r,y)\Vert _{L_q}^2 dy dr. \end{aligned}$$

In view of Proposition 5.2, we see that the norm in the integrand is bounded uniformly in (*r*, *y*). Hence using Lemma 11.4, we conclude that

5.10 $$\begin{aligned} F_{q,0}^{(2)}(t,x,z,\bar{z}) =\Vert \phi ^{z}(t,x) - \phi ^{\bar{z}}(t,x)\Vert _{L_q}&\lesssim \sup _{(t,w) \in [0,1]\times \mathbb {T}}|P_{t}(z-\bar{z})(w)|\nonumber \\&\lesssim \sup _{w \in \mathbb {T}}|z(w) - \bar{z}(w)|. \end{aligned}$$

By the triangle inequality, it follows that $F^{(2)}_{q,0}(t,x,\cdot ,\cdot ) \in C((C(\mathbb {T}))^2, \mathbb {R}).$ This implies that $F_{q,0}^{(2)}(t,x,Z,\bar{Z})$ is indeed defined as a random variable. We begin by showing that the desired inequality holds for the case when $\Vert Z\Vert _{\mathbb {B}},\Vert \bar{Z}\Vert _{\mathbb {B}} < N$ almost surely, for some constant N<∞. By evaluating (5.9) at $(z,\bar{z}) = (Z,\bar{Z})$, and then taking the $L_{p_1,p_2}^{\mathscr {G}}$-norm of the square root of both sides, we get

$$\begin{aligned}&\Vert F_{q,0}^{(2)}(t,x,Z,\bar{Z})\Vert _{L_{p_1,p_2}^{\mathscr {G}}}\\&\quad \lesssim \Vert P_{t}(Z-\bar{Z})(x)\Vert _{L_{p_1,p_2}^{\mathscr {G}}}+ \Big \Vert \int _0^t \int _{\mathbb {T}} p_{t-r}^2(x,y)|F_{q,0}^{(2)}(r,y,Z,\bar{Z})|^2 dydr\Big \Vert _{L_{\frac{p_1}{2},\frac{p_2}{2}}^{\mathscr {G}}}^{1/2}\\&\quad \lesssim \sup _{w \in \mathbb {T}}\Vert Z(w)-\bar{Z}(w)\Vert _{L_{p_1, p_2}^\mathscr {G}} \\&\quad \quad + \Big (\int _0^t \int _{\mathbb {T}} p_{t-r}^2(x,y)\Vert F_{q,0}^{(2)}(r,y,Z,\bar{Z})\Vert _{L_{p_1,p_2}^{\mathscr {G}}}^2 dydr\Big )^{1/2}, \end{aligned}$$

where to obtain the last expression, we used Minkowski’s inequality. Hence we may conclude that

$$\begin{aligned}\Vert F_{q,0}^{(2)}(t,x,Z,\bar{Z})\Vert _{L_{p_1,p_2}^{\mathscr {G}}}^2&\lesssim \Big (\sup _{w \in \mathbb {T}}\Vert Z(w)-\bar{Z}(w)\Vert _{L_{p_1,p_2}^{\mathscr {G}}}\Big )^2\\&\qquad \qquad + \int _0^t \int _{\mathbb {T}} p_{t-r}^2(x,y)\Vert F_{q,0}^{(2)}(r,y,Z,\bar{Z})\Vert _{L_{p_1,p_2}^{\mathscr {G}}}^2 dydr. \end{aligned}$$

Note that by (5.10) and due to the fact that $\Vert Z\Vert _{\mathbb {B}},\Vert \bar{Z}\Vert _{\mathbb {B}}\le N$, we have $\sup _{r,y}\Vert F_{q,0}^{(2)}(r,y,Z,\bar{Z})\Vert _{L_{p_1,p_2}^{\mathscr {G}}} \lesssim 2N$. Hence by the above inequality and by Lemma 11.4 we get

$$\Vert F^{(2)}_{q,0}(t,x,Z,\bar{Z})\Vert _{L_{p_1,p_2}^{\mathscr {G}}} \lesssim \sup _{w \in \mathbb {T}}\Vert Z(w) - \bar{Z}(w)\Vert _{L_{p_1,p_2}^{\mathscr {G}}}.$$

We now remove the assumption that $\Vert Z\Vert _{\mathbb {B}},\Vert \bar{Z}\Vert _{\mathbb {B}}<N$. Indeed, we define the sequence of truncations $(Z_N,{\bar{Z}}_N) := ((N\wedge Z) \vee (-N), (N\wedge \bar{Z}) \vee (-N))$. Then using the continuity of $F_{q,0}^{(2)}$ and Fatou’s lemma, the above inequality, and the fact that truncation is a Lipschitz operation, we get that

$$\begin{aligned} \Vert F_{q,0}^{(2)}(t,x,Z,\bar{Z})\Vert _{L_{p,\infty }^{\mathscr {G}}}&\le \liminf _{N \rightarrow \infty } \Vert F_{q,0}^{(2)}(t,x,Z_N,\bar{Z}_N)\Vert _{L_{p_1,p_2}^{\mathscr {G}}}\\&\lesssim \liminf _{N \rightarrow \infty } \sup _{w \in \mathbb {T}}\Vert Z_N(w) - \bar{Z}_N(w)\Vert _{L_{p_1,p_2}^{\mathscr {G}}}\\&\le \sup _{w \in \mathbb {T}}\Vert Z(w) - \bar{Z}(w)\Vert _{L_{p_1,p_2}^{\mathscr {G}}}. \end{aligned}$$

This finishes the proof of the case n=0.

Step 2: Suppose that for some $n \in \mathbb {N}$ the result holds for $F_{q,0}^{(2)},\dots , F_{q,n-1}^{(2)}$ for all q≥2. We aim to show that it also holds for $F_{q,n}^{(2)}$. We first assume that for all $(t,x)\in [0,1]\times \mathbb {T}$, $F_{q,n}^{(2)}(t,x,z,\bar{z})$ is continuous with respect to $(z,\bar{z})$ so that $F_{q,n}^{(2)}(t,x,Z,\bar{Z})$ is a well-defined random variable. We will verify this continuity property later. By Proposition 5.3, we have

5.11 $$\begin{aligned}&F^{(2)}_{q,n}(t,x,z,\bar{z})\nonumber \\&\quad =\Vert \mathscr {D}^n[\phi ^z(t,x) - \phi ^{\bar{z}}(t,x)]\Vert _{L_q(\Omega , H^{\otimes n})} \nonumber \\&\quad \lesssim \Big \Vert p_{t-\cdot }(x,\cdot )\mathscr {D}^{n-1}\big ( \sigma (\phi ^z(\cdot ,\cdot )) - \sigma (\phi ^{\bar{z}}(\cdot ,\cdot ))\big )\textbf{1}_{[0,t]}\Big \Vert _{L_q(\Omega ; H^{\otimes n})} \nonumber \\&\qquad + \Big \Vert \int _0^t \int _{\mathbb {T}} p_{t-r}(x,y) \mathscr {D}^n\big ( \sigma (\phi ^z(r,y)) - \sigma (\phi ^{\bar{z}}(r,y))\big )\xi (dy,dr)\Big \Vert _{L_q(\Omega ; H^{\otimes n})} \nonumber \\&\quad =: A(z,\bar{z})+B(z,\bar{z}). \end{aligned}$$

By Lemma 5.1 we may apply point (b) of Lemma 4.4 with $\varepsilon = s^{1/4}$, to see that for any m≤n and $(s,y) \in [0,1]\times \mathbb {T}$ we haveThus by using the induction hypothesis and the definition of $F^{(2)}$, we can see that

5.12 $$\begin{aligned} \Vert \Sigma _{q,m}^{(2)}(s,y,Z,\bar{Z})\Vert _{L_{p_1,p_2}^{\mathscr {G}}} \lesssim s^{ m/4}\sup _{w \in \mathbb {T}}\Vert Z(w) - \bar{Z}(w)\Vert _{L_{p_1,p_2}^{\mathscr {G}}}+ \Vert F_{q,m}^{(2)}(s,y,Z,\bar{Z})\Vert _{L_{p_1,p_2}^{\mathscr {G}}}. \end{aligned}$$

We can bound *A* by applying this result (with m:=n-1) as follows:

5.13 $$\begin{aligned}&\Vert A(Z,\bar{Z})\Vert _{L_{p_1,p_2}^{\mathscr {G}}} \nonumber \\&\quad \lesssim \big \Vert p_{t-\cdot }(x,\cdot )\big \Vert \Sigma _{q,n-1}^{(2)}(\cdot ,\cdot ,Z,\bar{Z})\big \Vert _{L_{p_1,p_2}^{\mathscr {G}}} \textbf{1}_{[0,t]}\big \Vert _{H} \nonumber \\&\quad \lesssim \Big \Vert p_{t-\cdot }(x,\cdot )\sup _{(\theta ,\zeta ) \in [0,t]\times \mathbb {T}}\Big ( \theta ^{\frac{n-1}{4}}\sup _{w \in \mathbb {T}}\Vert Z(w) -\bar{Z}(w)\Vert _{L_{p_1,p_2}^{\mathscr {G}}}\nonumber \\&\quad \quad + \Vert F_{q,n-1}^{(2)}(\theta ,\zeta ,Z,\bar{Z})\Vert _{L_{p_1,p_2}^{\mathscr {G}}} \Big )\textbf{1}_{[0,t]}\Big \Vert _H \nonumber \\&\quad \lesssim t^{n/4}\sup _{w \in \mathbb {T}}\Vert Z(w) -\bar{Z}(w)\Vert _{L_{p_1,p_2}^\mathscr {G}} , \end{aligned}$$

where for the last inequality we used the induction hypothesis. Now we also bound *B*. To this end, note that by the BDG inequality we haveand thus using (5.12) we get

5.14 $$\begin{aligned}&\Vert B(Z,\bar{Z})\Vert _{L_{p_1,p_2}^{\mathscr {G}}}^2 \nonumber \\&\quad \lesssim \int _0^t \int _{\mathbb {T}} p_{t-r}^2(x,y)\big \Vert \Sigma _{q,n}^{(2)}(r,y,Z,\bar{Z})\big \Vert _{L_{p_1,p_2}^{\mathscr {G}}}^2 dydr\nonumber \\&\quad \lesssim \int _0^t \int _{\mathbb {T}}p_{t-r}^2(x,y)\Big (t^{n/4}\sup _{w \in \mathbb {T}}\Vert Z(w) - \bar{Z}(w)\Vert _{L_{p_1,p_2}^{\mathscr {G}}}+\Vert F_{q,n}^{(2)}(r,y,Z,\bar{Z})\Vert _{L_{p_1,p_2}^{\mathscr {G}}} \Big )^2dydr\nonumber \\&\quad \lesssim t^{(n+1)/2}\sup _{w \in \mathbb {T}}\Vert Z(w) - \bar{Z}(w)\Vert _{L_{p_1,p_2}^{\mathscr {G}}}^2 + \int _0^t \int _{\mathbb {T}} p_{t-r}^2(x,y)\Vert F_{q,n}^{(2)}(r,y,Z,\bar{Z})\Vert _{L_{p_1,p_2}^{\mathscr {G}}}^2 dydr. \quad \end{aligned}$$

By putting the bounds (5.13) and (5.14) into (5.11) we can see that

5.15 $$\begin{aligned} \Vert F_{q,n}^{(2)}(t,x,Z,\bar{Z})\Vert _{L_{p_1,p_2}^{\mathscr {G}}}^2&\lesssim t^{n/2}\sup _{w \in \mathbb {T}}\Vert Z(w) - \bar{Z}(w)\Vert _{L_{p_1,p_2}^{\mathscr {G}}}^2 \nonumber \\&\qquad + \int _0^t \int _{\mathbb {T}} p_{t-r}^2(x,y)\Vert F_{q,n}^{(2)}(r,y,Z,\bar{Z})\Vert _{L_{p_1,p_2}^{\mathscr {G}}}^2 dydr. \end{aligned}$$

If $(Z,\bar{Z}) = (z,\bar{z}) \in (C(\mathbb {T}))^2$ is deterministic, then we may repeat the proof without assuming that $F_{q,n}^{(2)}(t,x,z,\bar{z})$ is continuous in $(z,\bar{z})$. The above inequality then simply states that

$$|F_{q,n}^{(2)}(t,x,z,\bar{z})|^2 \lesssim t^{n/2}\sup _{w \in \mathbb {T}}|z(w) - \bar{z}(w)|^2 + \int _0^t \int _{\mathbb {T}} p^2_{t-r}(x,y)|F_{q,n}^{(2)}(r,y,z,\bar{z})|^2dydr.$$

Note that by the definition of $F^{(2)}$ and by Proposition 5.2 we haveso using Lemma 11.4 we getFrom this it easily follows that for all $(t,x) \in [0,1]\times \mathbb {T}$ the map $F_{q,n}^{(2)}(t,x,\cdot ,\cdot )$ is continuous on $C(\mathbb {T})\times C(\mathbb {T})$. Now going back to (5.15), if $\Vert Z\Vert _{\mathbb {B}}, \Vert \bar{Z}\Vert _{\mathbb {B}} \le N$ for some given N>0 then the desired result follows by Lemma 11.4. For the general case we can repeat the truncation argument from the n=0 case to finish the proof. □

### Nondegeneracy of convex combinations of solutions

Throughout the section we assume that $\sigma \in C^1$ such that there exists a constant μ>0 such that for all $x \in \mathbb {R}$ we have $\sigma (x) \ge \mu$. Let $\phi ^1,\dots , \phi ^K$ solve the driftless equation (5.1) with initial conditions $\phi ^1_0,\dots , \phi ^K_0 \in C(\mathbb {T})$ respectively. Consider the convex combination

5.16 $$\begin{aligned} \Theta (t,x) := \sum _{i=1}^K c_i \phi ^i(t,x). \end{aligned}$$

with $c_1,\dots , c_K \in [0,1]$ satisfying $\sum _{i=1}^K c_i =1$. For a smooth map *g* and a nonnegative integer *n*, we aim to obtain estimates on the expectation of $\nabla ^n g(\Theta (t,x))$ which depend only on a Besov–Hölder norm of *g* with a negative index, see Lemma 5.10 below.

The following lemma quantifies Theorem 4.5 in the chapter by Nualart in [12].

#### Lemma 5.7

For any $p \in (2,\infty )$ there exists some constant $N = N(p,\Vert \sigma \Vert _{C^1}, \mu , K)$, such that for all t∈[0,1] we have

$$\sup _{x \in \mathbb {T}}\mathbb {E}[\Vert \mathscr {D}\Theta (t,x)\Vert _H^{-p} ]\le N t^{-p/4}.$$

#### Proof

By Proposition 5.3 we have for $(t,x), (\theta ,\zeta ) \in [0,1]\times \mathbb {T}$ that

$$\begin{aligned} \mathscr {D}_{\theta ,\zeta } \Theta (t,x)&= \textbf{1}_{[0,t]}(\theta )p_{t-\theta }(x,\zeta )\sum _{i=1}^K c_i \sigma (\phi ^i(\theta ,\zeta ))\\&\qquad + \textbf{1}_{[0,t]}(\theta )\int _0^t \int _{\mathbb {T}} p_{t-r}(x,y)\\&\qquad \Big (\sum _{i=1}^K c_i \nabla \sigma (\phi ^i(r,y)) \mathscr {D}_{\theta ,\zeta } \phi ^i(r,y) \Big )\xi (dy,dr)\\&=: A(\theta ,\zeta )+B(\theta ,\zeta ). \end{aligned}$$

From this we can see that for any $\delta \in (0,t)$,

$$\begin{aligned}\int _0^t \int _{\mathbb {T}} | \mathscr {D}_{\theta ,\zeta } \Theta (t,x)|^2 d\zeta d\theta&\ge \frac{1}{2}\int _{t-\delta }^t \int _{\mathbb {T}} | A(\theta ,\zeta )|^2 d\zeta d\theta - \int _{t-\delta }^t \int _{\mathbb {T}} | B(\theta ,\zeta )|^2 d\zeta d\theta \\&=: I_A^\delta - I_B^\delta . \end{aligned}$$

So since $|A(\theta ,\zeta )| \ge \textbf{1}_{[0,t]}(\theta )p_{t-\theta }(x,\zeta )\mu$, and by the properties of the heat kernel, it follows that

$$\begin{aligned}I_A^\delta = \frac{1}{2}\int _{t-\delta }^t \int _{\mathbb {T}}|A(\theta ,\zeta )|^2 d\zeta d\theta&\ge \mu ^2 \int _{t-\delta }^t \int _{\mathbb {T}}|p_{t-\theta }(x,\zeta )|^2 d\zeta d \theta \ge k\mu ^2 \delta ^{1/2} \end{aligned}$$

for some universal constant k>0. Thus

$$I_A^\delta \ge c_0 \delta ^{1/2} \quad \text {with} \quad c_0 = k \mu ^2.$$

Therefore for $\varepsilon \in (0, c_0\delta ^{1/2})$ we have

5.17 $$\begin{aligned} \mathbb {P}\Big (\int _0^t \int _{\mathbb {T}} | \mathscr {D}_{\theta ,\zeta } \Theta (t,x)|^2 d\zeta d\theta< \varepsilon \Big )&\le \mathbb {P}(I_A^\delta - I_B^\delta <\varepsilon ) \nonumber \\&\le \mathbb {P}(I_B^\delta >c_0\delta ^{1/2} - \varepsilon ) \nonumber \\&\le (c_0\delta ^{1/2} - \varepsilon )^{-p} \mathbb {E}|I_B^\delta |^p \end{aligned}$$

where the last inequality holds by Markov’s inequality. We will now need to bound the expectation in the last line. Note that

$$\begin{aligned} \mathbb {E}|I_B^\delta |^p&= \mathbb {E}\Big |\int _{t-\delta }^t \int _{\mathbb {T}} \Big | \int _0^t \int _{\mathbb {T}} p_{t-r}(x,y)\\&\qquad \Big (\sum _{i=1}^K c_i \nabla \sigma (\phi ^i(r,y)) \mathscr {D}_{\theta ,\zeta } \phi ^i(r,y) \Big )\xi (dy,dr)\Big |^2 d\zeta d\theta \Big |^p\\&= \mathbb {E}\Big \Vert \int _0^t \int _{\mathbb {T}} p_{t-r}(x,y)\Big (\sum _{i=1}^K c_i \nabla \sigma (\phi ^i(r,y)) \mathscr {D} \phi ^i(r,y) \Big )\xi (dy,dr)\Big \Vert _{L_2([t-\delta ,t]\times \mathbb {T})}^{2p}\\&\lesssim \mathbb {E}\Big (\int _{t-\delta }^t \int _{\mathbb {T}}|p_{t-r}(x,y)|^2 \sum _{i=1}^K \Vert \mathscr {D} \phi ^i(r,y)\Vert _{L_2([t-\delta ,t]\times \mathbb {T})}^2 dydr\Big )^{p} \end{aligned}$$

where we used the BDG inequality, and the fact that $\Vert \mathscr {D} \phi ^i(r,y)\Vert _{L_2([t-\delta ,t]\times \mathbb {T})} =0$ for r<t-δ. Noting that $r-\delta <t-\delta$, and that $\mathscr {D}_{\theta ,\zeta } \phi ^i(r,y) =0$ for θ>r, we may bound the $L_2([t-\delta ,t]\times \mathbb {T})$ norm in the expression by the $L_2([r-\delta ,r]\times \mathbb {T})$-norm, and write

5.18 $$\begin{aligned} \mathbb {E}|I_B^\delta |^p&\lesssim \sum _{i=1}^K \mathbb {E}\Big (\int _{t-\delta }^t \int _{\mathbb {T}} |p_{t-r}(x,y)|^2 \Vert \mathscr {D} \phi ^i(r,y)\Vert _{L_2([r-\delta ,r]\times \mathbb {T})}^2 dydr\Big )^{p} \nonumber \\&\le \delta ^{p/2 -1}\sum _{i=1}^K \int _{t-\delta }^t \int _{\mathbb {T}} p_{t-r}(x,y) \mathbb {E}\Vert \mathscr {D} \phi ^i(r,y) \Vert _{L_2([r-\delta , r]\times \mathbb {T})}^{2p} dydr \nonumber \\&=: \delta ^{p/2-1}\sum _{i=1}^K H_i \end{aligned}$$

where we used Lemma 11.5 (with γ=1). To bound $H_i$, we will need to bound $\Vert \mathscr {D} \phi ^i(t,x)\Vert _{L_2([t-\delta , t]\times \mathbb {T})}$ for each (*t*, *x*). To this end, note that for $(\theta ,\zeta ) \in [0,t]\times \mathbb {T}$ we have

$$\begin{aligned}\mathscr {D}_{\theta ,\zeta } \phi ^i(t,x)&= p_{t-\theta }(x,\zeta )\sigma (\phi ^i(\theta ,\zeta ))\\  &\quad + \int _0^t \int _{\mathbb {T}} p_{t-r}(x,y) \nabla \sigma (\phi ^i(r,y)) \mathscr {D}_{\theta ,\zeta }\phi ^i(r,y) \xi (dy,dr). \end{aligned}$$

For any q>4, by using the BDG inequality, we get

$$\begin{aligned}&\mathbb {E}\Vert \mathscr {D} \phi ^i(t,x)\Vert _{L_2([t-\delta , t] \times \mathbb {T})}^q \\&\quad \lesssim \Vert p_{t-\cdot }(x,\cdot )\Vert _{L_2([t-\delta , t] \times \mathbb {T})}^q \\&\qquad + \mathbb {E}\Big (\int _0^t \int _{\mathbb {T}} |p_{t-r}(x,y)|^2 \Vert \mathscr {D} \phi ^i(r,y)\Vert _{L_2([t-\delta , t] \times \mathbb {T})}^2 dydr\Big )^{q/2}\\&\quad =: \bar{A} + \bar{B}. \end{aligned}$$

We have $\bar{A} \lesssim \delta ^{q/4}$. Moreover, applying Lemma 11.5 (with γ=2) to $\bar{B}$ and that $\Vert \mathscr {D} \phi ^i(r,y)\Vert _{L_2([t-\delta ,t]\times \mathbb {T})} \le \Vert \mathscr {D} \phi ^i(r,y)\Vert _{L_2([r-\delta ,r]\times \mathbb {T})}$, we get that

$$\begin{aligned} \bar{B}&\lesssim \int _0^t \int _{\mathbb {T}} |p_{t-r}(x,y)|^2 \mathbb {E}\Vert \mathscr {D} \phi ^i(r,y)\Vert _{L_2([r-\delta , r] \times \mathbb {T})}^{q} dydr. \end{aligned}$$

Therefore we obtain that

$$\mathbb {E}\Vert \mathscr {D} \phi ^i(t,x)\Vert _{L_2([t-\delta , t] \times \mathbb {T})}^q \lesssim \delta ^{q/4} + \int _0^t \int _{\mathbb {T}} |p_{t-r}(x,y)|^2 \mathbb {E}\Vert \mathscr {D} \phi ^i(r,y)\Vert _{L_2([r-\delta , r] \times \mathbb {T})}^{q} dydr.$$

By Proposition 5.2 we can see that the *q*-th moment in the integrand is bounded uniformly in (*r*, *y*). Hence by Lemma 11.4 it follows that

$$\mathbb {E}\Vert \mathscr {D} \phi ^i(t,x)\Vert _{L_2([t-\delta ,t]\times \mathbb {T})}^q \lesssim \delta ^{q/4}.$$

Applying this with q=2p to bound $H_i$, we get

5.19 $$\begin{aligned} H_i \lesssim \int _{t-\delta }^t \int _{\mathbb {T}}p_{t-r}(x,y) \delta ^{p/2}dydr \lesssim \delta ^{p/2+1}. \end{aligned}$$

Now putting (5.19) into (5.18), we get $\mathbb {E}|I_B^\delta |^p \lesssim \delta ^p$. Putting this into (5.17) we see that for all $\delta \in [0, t]$ and all $\varepsilon \in (0, c_0\delta ^{1/2})$, we have

$$\mathbb {P}(\Vert \mathscr {D} \Theta (t,x)\Vert _{H}^2 <\varepsilon ) \lesssim (c_0 \delta ^{1/2}- \varepsilon )^{-p}\delta ^{p}.$$

So if $\varepsilon \in (0, (c_0/2) \sqrt{t})$, we can choose $\delta (\varepsilon ) := \frac{4}{c_0^2}\varepsilon ^2$, to get

$$\mathbb {P}(\Vert \mathscr {D} \Theta (t,x)\Vert _{H}^2 <\varepsilon ) \lesssim \varepsilon ^p.$$

Let $L:= (2/c_0)^{p/2} t^{-p/4}$. Notice that if γ>L, then $\gamma ^{-2/p} \in (0, (c_0/2) \sqrt{t})$, and consequently we have

$$\mathbb {P}(\Vert \mathscr {D} \Theta (t,x)\Vert _{H}^2 < \gamma ^{-2/p}) \lesssim \gamma ^{-2} .$$

Hence, we have

$$\begin{aligned} \mathbb {E}\Vert \mathscr {D}\Theta (t,x)\Vert _{H}^{-p} = \int _0^\infty \mathbb {P}\big (\Vert \mathscr {D} \Theta \Vert _H^{-p} \ge \gamma \big ) d\gamma&\le L+ \int _L^\infty \mathbb {P}(\Vert \mathscr {D}\Theta \Vert _H^2 < \gamma ^{-2/p}) d\gamma \\&\lesssim L + \int _{L}^\infty \gamma ^{-2} d\gamma \\&\lesssim L + L^{-1} \lesssim t^{-p/4}, \end{aligned}$$

which finishes the proof. □

For $(t,x) \in [0,1]\times \mathbb {T}$, we consider the *H*-valued random variables $w_{t,x}$ which are given for all $(\theta ,\zeta ) \in [0,1]\times \mathbb {T}$ by

$$w_{t,x}(\theta ,\zeta ) := \frac{\mathscr {D}_{\theta ,\zeta }\Theta (t,x)}{\Vert \mathscr {D}\Theta (t,x)\Vert _{H}^2}.$$

For given $n \in \mathbb {N}$ and for a $C([0,1]\times \mathbb {T})$-valued random variable $G_0$ such that for all $(t,x) \in [0,1]\times \mathbb {T}$ $G_0(t,x) \in \mathscr {W}^n$, we may define iterated Skorokhod integrals for all $k \in \{0,\dots , n-1\}$ and $(t,x) \in [0,1]\times \mathbb {T}$ recursively by

$$\begin{aligned} G_{k+1}(t,x)&= \delta (w_{t,x} G_k(t,x)). \end{aligned}$$

Then by Proposition 4.2, for any $f \in C^\infty$ we have the integration-by-parts formula

$$\mathbb {E}\big (\nabla ^k f(\Theta ) G_0\big ) = \mathbb {E}\big (f(\Theta )G_k\big ).$$

To bound the iterations $(G_k)_{k \in \{0,\dots , n\}}$, we will need the following bounds on *w* and its Malliavin derivatives.

#### Lemma 5.8

Let $p \in [1,\infty )$, $n \in \mathbb {Z}_{\ge 0}$, and $\sigma \in C^{n+1}$. Then there exists a constant $N = N(p,n, \Vert \sigma \Vert _{C^{n+1}}, \mu )$ such that for all $(t,x) \in [0,1]\times \mathbb {T}$, we have

$$\Vert \mathscr {D}^n w_{t,x}\Vert _{L_p(\Omega ;H^{\otimes (n+1)})} \le N t^{(n-1)/4}.$$

#### Proof

Fix $(t,x) \in [0,1]\times \mathbb {T}$ and let X:=Θ(t,x), $Y := \Vert \mathscr {D}\Theta (t,x)\Vert _H^2$ and $w := (\mathscr {D}X)/Y$. We may assume that p>2. By Lemma 5.1, Lemma 5.5 and Lemma 5.7 respectively, we haveTherefore by Lemma 4.5 (with $\varepsilon := t^{1/4}$ and c=2) to obtain

$$\Vert \mathscr {D}^n w\Vert _{L_p(\Omega ; H^{\otimes (n+1)})} \lesssim (t^{1/4})^{n+1 -2} \lesssim t^{(n-1)/4}$$

as required. □

#### Lemma 5.9

Let $n \in \mathbb {Z}_{\ge 0}$, and $\sigma \in C^n$. Then for each $k,m \in \mathbb {Z}_{\ge 0}$ such that k+m≤n and for all $p \in [1,\infty )$ there exists a constant $N = N(k,m,p, \Vert \sigma \Vert _{C^n}, \mu )$ such that with $q := 2^m p$ we have for all $(t,x) \in [0,1]\times \mathbb {T}$ that

$$\begin{aligned}\Vert G_m(t,x) \Vert _{\mathscr {W}_p^k} \le Nt^{-m/4}\Vert G_0(t,x)\Vert _{\mathscr {W}_{q}^{k+m}}. \end{aligned}$$

#### Proof

For notational convenience, fix $(t,x) \in [0,1]\times \mathbb {T}$, set $w := w_{t,x}$, and for i=1,⋯,n set $G_i:= G_i(t,x)$. The proof will be done by induction with respect to the *m* variable. For m=0 the statement is obviously true. Now suppose that the statement is true for some m≤n-1. That is, we suppose that for all $l \in \mathbb {Z}_{\ge 0}$ such that l+m≤n we have

$$\Vert G_m\Vert _{\mathscr {W}_p^l} \lesssim t^{-m/4}\Vert G_0\Vert _{\mathscr {W}_{2^m p}^{l+m}}.$$

We show that the statement is also true for m+1, i.e. that for all $k \in \mathbb {Z}_{\ge 0}$ such that k+(m+1)≤n, we have

5.20 $$\begin{aligned} \Vert G_{m+1}\Vert _{\mathscr {W}_p^k} \lesssim t^{-(m+1)/4}\Vert G_0\Vert _{\mathscr {W}_{2^{m+1}p}^{k+m+1}}. \end{aligned}$$

Let $k \in \mathbb {Z}_{\ge 0}$, such that k+m+1≤n. Since the divergence $\delta : \mathscr {W}_p^{k+1} \rightarrow \mathscr {W}_p^k$ is a bounded linear operator (see [31, Proposition 1.5.7 and point 1 of remarks of Chapter 1]), we have

5.21 $$\begin{aligned} \Vert G_{m+1}\Vert _{\mathscr {W}_p^k} = \Vert \delta (w G_{m}) \Vert _{\mathscr {W}_p^k}&\lesssim \Vert w G_{m} \Vert _{\mathscr {W}_p^{k+1}(H)}\nonumber \\  &\lesssim \sum _{i=0}^{k+1} \Vert \mathscr {D}^i(wG_{m}) \Vert _{L_p(\Omega ;H^{\otimes (i+1)})} \nonumber \\&\lesssim \sum _{i=0}^{k+1}\sum _{\lambda _1 + \lambda _2 = i} \Vert \mathscr {D}^{\lambda _1}w\Vert _{L_{2p}(\Omega ; H^{\otimes (\lambda _1 +1)})}\Vert \mathscr {D}^{\lambda _2} G_m\Vert _{L_{2p}(\Omega ; H^{\otimes \lambda _2})} . \end{aligned}$$

By Lemma 5.8, and since $\lambda _1\ge 0$, we have

5.22 $$\begin{aligned} \Vert \mathscr {D}^{\lambda _1}w\Vert _{L_{2p}(\Omega ; H^{\otimes (\lambda _1 +1)})} \lesssim t^{(\lambda _1 -1)/4} \lesssim t^{-1/4}. \end{aligned}$$

Moreover since $\lambda _2 + m \le k+1+m \le n$, by the induction hypothesis we have

5.23 $$\begin{aligned} \Vert \mathscr {D}^{\lambda _2} G_m\Vert _{L_{2p}(\Omega ; H^{\otimes \lambda _2})} \le \Vert G_m\Vert _{\mathscr {W}_{2p}^{\lambda _2}} \lesssim t^{-m/4}\Vert G_0\Vert _{\mathscr {W}_{2^{m +1} p}^{\lambda _2+m}} \le t^{-m/4}\Vert G_0\Vert _{\mathscr {W}_{2^{m+1}p}^{k+1+m}} . \end{aligned}$$

Now putting (5.22) and (5.23) into (5.21), we get

$$\Vert G_{m+1}\Vert _{\mathscr {W}_p^k} \lesssim t^{-1/4}t^{-m/4}\Vert G_0\Vert _{\mathscr {W}_{2^{m+1}p}^{k+1+m}}.$$

Hence (5.20) holds, and the proof is finished. □

#### Lemma 5.10

Let $n \in \mathbb {Z}_{\ge 0}$, $\sigma \in C^{n+1}$, $\beta \in (-2,-1)\cup (-1,0)$, and set $q:= 2^{n+2}\textbf{1}_{(-1,0)}(\beta ) + 2^{n+3}\textbf{1}_{(-2,-1)}(\beta )$ and $m := (n+1)\textbf{1}_{(-1,0)}(\beta ) + (n+2)\textbf{1}_{(-2,-1)}(\beta )$. There exists a constant $N = N(n,\beta , \Vert \sigma \Vert _{C^{n+1}}, \mu )$ such that for all $g \in C^\infty$, $(t,x) \in [0,1]\times \mathbb {T}$, we have

$$\big |\mathbb {E}\big (\nabla ^n g(\Theta (t,x)) G_0(t,x)\big )\big | \le N\Vert g\Vert _{C^\beta } t^{\frac{\beta -n}{4}}\Vert G_0(t,x)\Vert _{\mathscr {W}_{q}^{m}}.$$

#### Proof

Let $f \in C^\infty$ be the solution of (1-Δ)f=g and let $(t,x) \in [0,1]\times \mathbb {T}$. By Proposition 4.2 and by the definition of *f*, we get

$$\begin{aligned} |\mathbb {E}(\nabla ^n g(\Theta (t,x)) G_0(t,x))|&= |\mathbb {E}( g(\Theta (t,x)) G_n(t,x))|\\&\le | \mathbb {E}(f(\Theta (t,x))G_n(t,x))|\\&\quad +|\mathbb {E}(\Delta f(\Theta (t,x))G_n(t,x))| =:A+B. \end{aligned}$$

It follows easily from Lemma 5.9 and Proposition 11.8 that

5.24 $$\begin{aligned} A \le \Vert f\Vert _{\mathbb {B}}\Vert G_n(t,x)\Vert _{L_1}&\le \Vert f\Vert _{C^{2+\beta }}t^{-n/4}\Vert G_0(t,x)\Vert _{\mathscr {W}_{2^n}^n}\nonumber \\  &\lesssim \Vert g\Vert _{C^{\beta }} t^{-n/4 + \beta /4}\Vert G_0(t,x)\Vert _{\mathscr {W}_{q}^{m}}.\quad \quad \end{aligned}$$

It remains to be shown that the desired bound also holds on *B*. To this end, we first note that by Jensen’s inequality and by the BDG inequality for $\gamma \in (0,1)$ we have

5.25 $$\begin{aligned} \Big \Vert \Big (\Theta (t,x) - \sum _{i=1}^K c_i P_t \phi ^i(0,\cdot )(x)\Big )^{\gamma }\Big \Vert _{L_2}&\le \Big \Vert \sum _{i=1}^K c_i\Big (\phi ^i(t,x) - P_t \phi ^i(0,\cdot )(x)\Big )\Big \Vert _{L_{2}}^{\gamma }\nonumber \\&\le \sum _{i=1}^K \Big \Vert \int _0^t \int _{\mathbb {T}} p_{t-r}(x,y)\sigma (\phi ^i(r,y)) \xi (dy,dr)\Big \Vert _{L_{2}}^{\gamma } \nonumber \\&\lesssim t^{\gamma /4}. \end{aligned}$$

We first consider the case that $\beta \in (-1,0)$. By Proposition 4.2, the fact that $\mathbb {E}G_{n+1} =0$, and (5.25) with $\gamma = 1+\beta \in (0,1)$, we get

5.26 $$\begin{aligned} B&= |\mathbb {E}(\nabla f(\Theta (t,x))G_{n+1}(t,x))| \nonumber \\&= \Big |\mathbb {E}\Big (\Big (\nabla f(\Theta (t,x)) - \nabla f\Big (\sum _{i=1}^K c_i P_t \phi ^i(0,\cdot )(x)\Big )\Big ) G_{n+1}(t,x))\Big )\Big |\nonumber \\&\lesssim \Vert \nabla f\Vert _{C^{ 1+\beta }}\Big \Vert \Big (\Theta (t,x) - \sum _{i=1}^K c_i P_t \phi ^i(0,\cdot )(x)\Big )^{1+\beta }\Big \Vert _{L_2}\Vert G_{n+1}(t,x)\Vert _{L_2}\nonumber \\&\lesssim \Vert f\Vert _{C^{2+\beta }} t^{\frac{1+\beta }{4}}\Vert G_{n+1}(t,x)\Vert _{L_2}\nonumber \\&\lesssim \Vert g\Vert _{C^\beta }t^{\frac{\beta - n}{4}}\Vert G_0(t,x)\Vert _{\mathscr {W}_{q}^{n+1}}, \end{aligned}$$

where for the last inequality we used Proposition 11.8 and Lemma 5.9. We now also deal with the case when $\beta \in (-2,-1)$. Repeating the same steps with one more iteration of Malliavin integration by parts and with $\gamma = 2+\beta \in (0,1)$, we can see that

5.27 $$\begin{aligned} B&= |\mathbb {E}(f(\Theta (t,x))G_{n+2}(t,x))|\nonumber \\&\lesssim \Vert f\Vert _{C^{ 2+\beta }}\Big \Vert \Big (\Theta (t,x) - \sum _{i=1}^K c_i P_t \phi ^i(0,\cdot )(x)\Big )^{2+\beta }\Big \Vert _{L_2}\Vert G_{n+2}(t,x)\Vert _{L_2}\nonumber \\&\lesssim \Vert g\Vert _{C^\beta }t^{\frac{\beta - n}{4}}\Vert G_0(t,x)\Vert _{\mathscr {W}_{q}^{n+2}}.\qquad \end{aligned}$$

By (5.26) and (5.27) we can see that for all $\beta \in (-2,-1) \cup (-1,0)$ we have

5.28 $$\begin{aligned} B \lesssim \Vert g\Vert _{C^\beta }t^{\frac{\beta - n}{4}}\Vert G_0(t,x)\Vert _{\mathscr {W}_{q}^{m}}. \end{aligned}$$

By the bounds (5.24) and (5.28) on *A* and *B* respectively, the proof is finished. □

Let s≥0 and suppose that $Z:\Omega \times \mathbb {T} \rightarrow \mathbb {R}$ is an $\mathscr {F}_s \otimes \mathscr {B}(\mathbb {T})$-measurable map, such that *Z*(*x*) is continuous in *x* and that $\sup _{x \in \mathbb {T}}\Vert Z(x)\Vert _{L_2} <\infty$. Let $\phi ^{Z,s}$ denote the solution of

5.29 $$\begin{aligned} (\partial _t - \Delta )\phi ^{Z,s} = \sigma (\phi ^{Z,s})\xi \qquad \text {in }(s, 1) \times \mathbb {T}, \qquad \phi ^{Z,s}_s = Z. \end{aligned}$$

For $(t,x) \in [s,1]\times \mathbb {T}$, the solution satisfies the integral equation

5.30 $$\begin{aligned} \phi ^{Z,s}(t,x) = P_{t-s}Z(x) + \int _s^t \int _{\mathbb {T}}p_{t-r}(x,y) \sigma (\phi ^{Z,s}(r,y)) \xi (dy,dr). \end{aligned}$$

We will use the shorthand

5.31 $$\begin{aligned} \phi ^Z(t,x) := \phi ^{Z,0}(t,x). \end{aligned}$$

#### Lemma 5.11

Let $z_1,\dots , z_K \in C(\mathbb {T})$, $g \in C^\infty (\mathbb {R})$ and $h:\mathbb {R}^K\rightarrow \mathbb {R}$ be a smooth function whose derivatives have polynomial growth. For $(t,x)\in [0,1]\times \mathbb {T}$, s∈[0,t], $q \in [1,\infty )$ and $i \in \mathbb {Z}_{\ge 0}$ define $H_{q,i}: (C(\mathbb {T}))^K \rightarrow \mathbb {R}$ byLet $Z_1,\dots ,Z_K$ be $\mathscr {F}_s$-measurable $C(\mathbb {T})$-valued random variables and define

$$\begin{aligned} \Phi ^{Z_1,\dots ,Z_K,s}(t,x)&:= \sum _{i=1}^K c_i \phi ^{Z_i,s}(t,x), \end{aligned}$$

where $c_i$’s are the constants as in (5.16). For all $n \in \mathbb {Z}_{\ge 0}$, $\beta \in (-2,-1)\cup (-1,0)$ there exists a constant $N = N(n, \beta , \Vert \sigma \Vert _{C^{n+1}},\mu )$ such that with $q := 2^{n+2}\textbf{1}_{(-1,0)}(\beta ) + 2^{n+3}\textbf{1}_{(-2,-1)}(\beta )$ and $m:= (n+1)\textbf{1}_{(-1,0)}(\beta ) + (n+2)\textbf{1}_{(-2,-1)}(\beta )$, we have

$$\begin{aligned}&\Big |\mathbb {E}^s\Big ( \nabla ^n g\Big (\Phi ^{Z_1,\dots , Z_K,s}(t,x)\Big ) h\big (\phi ^{Z_1,s}(t,x),\dots , \phi ^{Z_K,s}(t,x)\big ) \Big )\Big | \\  &\qquad \qquad \qquad \qquad \qquad \qquad \le N\Vert g\Vert _{C^\beta } (t-s)^{\frac{\beta -n}{4}}\sum _{i=0}^{m}H_{q,i}\big (Z_1,\dots , Z_K\big ). \end{aligned}$$

#### Proof

By Lemma 11.11 we have

5.32 $$\begin{aligned} \Big |\mathbb {E}^s\Big ( \nabla ^n g\big (\Phi ^{Z_1,\dots , Z_K,s}(t,x)\big ) h\big (\phi ^{Z_1,s}(t,x),\dots , \phi ^{Z_K,s}(t,x)\big ) \Big )\Big | = G(Z_1,\dots ,Z_K), \end{aligned}$$

where for $z_1, \dots , z_K \in C(\mathbb {T})$ we define

$$G(z_1,\dots ,z_K) := \Big |\mathbb {E}\Big (\nabla ^n g\Big (\sum _{i=1}^K c_i \phi ^{z_i}(t-s,x)\Big ) h(\phi ^{z_i}(t-s,x),\dots , \phi ^{z_K}(t-s,x))\Big )\Big |.$$

By Lemma 5.10 we have5.33Now putting (5.33) into (5.32), the desired result follows. □

## Driftless approximation

In this section, we establish error estimates for the approximation $\phi ^{u(s, \cdot ),s}(t,x)$ to the solution *u*(*t*, *x*). The main results of this section are Lemma 6.4 and Lemma 6.6.

### Lemma 6.1

Let *u* be a regularised solution of (1.1) with initial condition $u(0,\cdot ) = u_0 \in C(\mathbb {T})$ and let $p \in [2,\infty )$. There exists a constant $N = N(p,\Vert \sigma \Vert _{\mathbb {B}})$ such that for all $(t,x) \in [0,1]\times \mathbb {T}$ we have

6.1 $$\begin{aligned} \Vert u(t,x)\Vert _{L_p} \le N\Big (\Vert u_0\Vert _{\mathbb {B}(\mathbb {T})}+ \Vert D^u_t(x)\Vert _{L_p} + t^{1/4}\Big ). \end{aligned}$$

Consequently, if $u \in \mathscr {U}_p^0$, then

6.2 $$\begin{aligned} \sup _{(t,x) \in [0,1]\times \mathbb {T}}\Vert u(t,x)\Vert _{L_p} <\infty . \end{aligned}$$

### Proof

From (3.2) and the BDG inequality we can see that

$$\begin{aligned}\Vert u(t,x)\Vert _{L_p}^2&\lesssim \Vert u_0\Vert _{\mathbb {B}(\mathbb {T})}^2 + \Vert D_t^u(x)\Vert _{L_p}^2 + \int _0^t \int _{\mathbb {T}}p_{t-r}^2(x,y)\Vert \sigma \Vert _{\mathbb {B}}^2dydr, \end{aligned}$$

and thus the inequality (6.1) follows. Now suppose that $u \in \mathscr {U}_p^0$. Then noting that $D_t^u = D_t^u - P_{t-0}D_0^u$, we have

$$\sup _{(t,x) \in [0,1]\times \mathbb {T}}\Vert D^u_t(x)\Vert _{L_p} \le [D^u]_{\mathscr {V}_p^0} <\infty .$$

Also, note that by Assumption 3.1 the initial condition u(0,·) is bounded. Therefore all terms on the right hand side of (6.1) are bounded in (*t*, *x*), and thus (6.2) follows. □

Recall that the random fields $\phi ^{z,s}(t,x)$ and $\phi ^z(t,x)$ are defined by (5.30) and (5.31) respectively. Let $\sigma \in C^1$, $\alpha \in (-1,0)$, $\beta \in [0,1]$, $p \in [2,\infty )$, and for i=1,2 let $b^i \in C^{\alpha }$, and suppose that $u^i \in \mathscr {U}_p^\beta$ are regularised solutions of the stochastic reaction–diffusion equations

6.3 $$\begin{aligned} (\partial _t - \Delta )u^i = b^i(u^i) + \sigma (u^i)\xi . \end{aligned}$$

For $(S,T) \in [0,1]_{\le }^2$ we define the $\mathscr {S}_p^{\beta }[S,T]$*-bracket* of $u^1$ and $u^2$ by

6.4 $$\begin{aligned} [ u^1,u^2]_{\mathscr {S}^{\beta }_p[S,T]} := \sup _{x \in \mathbb {T}}\sup _{(s,t)\in [S,T]_{<}^2}\frac{ \Vert u^1(t,x) - \phi ^{u^1(s,\cdot ),s}(t,x) - u^2(t,x) + \phi ^{u^2(s,\cdot ),s}(t,x) \Vert _{L_p}}{|t-s|^{\beta }}. \end{aligned}$$

### Remark 6.2

Note that for all s∈[0,1], by definition, the random field $u^i(s,x)$ is continuous in *x*, and by Lemma 6.1 we have $\sup _{x \in \mathbb {T}}\Vert u^i(s,x)\Vert _{L_p} <\infty$. Therefore the equation (5.29) starting from $u^i(s,\cdot )$ does indeed have a unique solution, which is denoted by $\phi ^{u^i(s,\cdot ),s}(t,x)$. Consequentially, the expression (6.4) is well-defined.

For brevity, we will use the convention

$${[}u^1,u^2]_{\mathscr {S}_p^{\beta }} := [u^1,u^2]_{\mathscr {S}_p^{\beta }[0,1]} \quad \text {and}\quad [f]_{\mathscr {V}_p^\beta } :=[f]_{\mathscr {V}_p^\beta [0,1]},$$

where the last notation is defined in Definition 3.4. By the triangle inequality and by Lemma 5.6 the following result holds:

### Lemma 6.3

Let $\sigma \in C^1$; $b^1,b^2 \in C^\alpha$; $\beta \in (0,1]$; $p \in [2,\infty )$ and let $u^1,u^2$ be regularised solutions of (6.3) in the class $\mathscr {U}_p^\beta$. There exists some constant $N =N(p, \Vert \sigma \Vert _{C^1}, \alpha , \beta )$ such that for all $(s,t) \in [0,1]_{\le }^2$ we have

$$\Vert u^1(t,\cdot ) - u^2(t,\cdot )\Vert _{\mathbb {B}(\mathbb {T}, L_p)} \le [u^1,u^2]_{\mathscr {S}_p^{\beta }[s,t]}(t-s)^\beta + N\Vert u^1(s,\cdot ) - u^2(s,\cdot )\Vert _{\mathbb {B}(\mathbb {T}, L_p)}.$$

### Lemma 6.4

Let $\sigma \in C^1$, $\alpha \in (-1,0)$, $b \in C^{\alpha }$. Let $p \in [2,\infty )$, $\beta \in [0, 1+ \frac{\alpha }{4}]$ and let *u* be a regularised solution of (1.1) in the class $\mathscr {U}_p^\beta$. Then there exists a constant $N = N(p, \Vert \sigma \Vert _{C^1}, \alpha , \beta )$ such that for all $0\le s \le t \le 1$, we have

$$\sup _{x \in \mathbb {T}}\Vert u(t,x) - \phi ^{u(s,\cdot ),s}(t,x)\Vert _{L_{p,\infty }^{\mathscr {F}_s}} \le N [D^u]_{\mathscr {V}_p^\beta [s,t]}(t-s)^{\beta }.$$

### Proof

By splitting the stochastic integral in (5.2) at time *s*, we have

6.5 $$\begin{aligned} u(t,x)&=P_tu(0,\cdot )(x) + D_t^u(x) + \int _0^s \int _{\mathbb {T}} p_{t-r}(x,y)\sigma (u(r,y))\xi (dy,dr) \nonumber \\&\qquad \qquad \qquad \qquad \qquad + \int _s^t \int _{\mathbb {T}} p_{t-r}(x,y)\sigma (u(r,y))\xi (dy,dr). \end{aligned}$$

Moreover using (5.30) to compute $\phi ^{u(s,\cdot ),s}(t,x)$ and then (3.2) to express u(s,·), we get

6.6 $$\begin{aligned} \phi ^{u(s,\cdot ),s}(t,x)&= P_{t-s}\Big (P_s u(0,\cdot )+ D_s^u(\cdot ) + \int _0^s \int _{\mathbb {T}}p_{s-r}(\cdot ,y)\sigma (u(r,y))\xi (dy,dr)\Big )(x)\nonumber \\&\qquad \qquad \qquad \qquad \qquad + \int _s^t \int _{\mathbb {T}}p_{t-r}(x,y)\sigma (\phi ^{u(s,\cdot ),s}(r,y))\xi (dy,dr)\nonumber \\&=P_{t}u(0,\cdot )(x) + P_{t-s}D_s^u(x) + \int _0^s\int _{\mathbb {T}} p_{t-r}(x,y)\sigma (u(r,y))\xi (dy,dr) \nonumber \\&\qquad \qquad \qquad \qquad \qquad + \int _s^t \int _{\mathbb {T}}p_{t-r}(x,y)\sigma (\phi ^{u(s,\cdot ),s}(r,y))\xi (dy,dr) \end{aligned}$$

where the last equality follows from the semigroup property that $P_{t-s}P_s = P_t$. Comparing (6.5) and (6.6), we can see that the error of the driftless approximation is given by

$$\begin{aligned}&u(t,x) - \phi ^{u(s,\cdot ),s}(t,x)\\&\quad = D_t^u(x) - P_{t-s}D_s^u(x) \\&\qquad + \int _s^t \int _{\mathbb {T}}p_{t-r}(x,y)\big ( \sigma (u(r,y)) -\sigma (\phi ^{u(s,\cdot ),s}(r,y))\big )\xi (dy,dr). \end{aligned}$$

Hence by the conditional BDG inequality (see Lemma 11.6), we get

6.7 $$\begin{aligned}&\Vert u(t,x) - \phi ^{u(s,\cdot ),s}(t,x)\Vert _{L_p | \mathscr {F}_s}^2 \nonumber \\&\quad \lesssim \Vert D_t^u(x) - P_{t-s}D_s^u(x)\Vert _{L_p|\mathscr {F}_s}^2\nonumber \\&\qquad + \int _s^t \int _{\mathbb {T}}p^2_{t-r}(x,y)\big \Vert \sigma (u(r,y)) -\sigma (\phi ^{u(s,\cdot ),s}(r,y))\big \Vert _{L_p | \mathscr {F}_s}^2dydr. \end{aligned}$$

Therefore

6.8 $$\begin{aligned} \Vert u(t,x) - \phi ^{u(s,\cdot ),s}(t,x)\Vert _{L_{p,\infty }^{\mathscr {F}_s}}^2&\lesssim [D^u]_{\mathscr {V}_p^\beta [s,t]}^2(t-s)^{2\beta } \nonumber \\&+\int _s^t \int _{\mathbb {T}}p^2_{t-r}(x,y)\Vert u(r,y) - \phi ^{u(s,\cdot ),s}(r,y)\Vert _{L_{p,\infty }^{\mathscr {F}_s}}^2 dydr. \end{aligned}$$

From (6.7) and the fact that $u \in \mathscr {U}_p^\beta$, we see that

$$\begin{aligned}&\sup _{(t,x)\in [0,1]\times \mathbb {T}}\Vert u(t,x) - \phi ^{u(s,\cdot ),s}(t,x)\Vert _{L_{p,\infty }^{\mathscr {F}_s}} \lesssim [D^u]_{\mathscr {V}_p^0} + \Vert \sigma \Vert _{\mathbb {B}} <\infty . \end{aligned}$$

Hence by (6.8) and by Lemma 11.4, we obtain that

$$\sup _{x \in \mathbb {T}}\Vert u(t,x) - \phi ^{u(s,\cdot ),s}(t,x)\Vert _{L_{p,\infty }^{\mathscr {F}_s}}^2 \lesssim [D^u]^2_{\mathscr {V}_p^\beta [s,t]}(t-s)^{2\beta },$$

which implies the desired result. □

### Assumption 6.5

Let $\alpha \in (-1,0)$, $b \in C^\alpha$, $n \in \mathbb {Z}_{\ge 0}$, and let $\sigma \in C^{n+2}$ such that there exists a constant μ>0 such that for all $x \in \mathbb {R}$, $\sigma ^2(x) > \mu ^2$. Let $\beta \in [\frac{1}{2} ,1+\frac{\alpha }{4}]$, and suppose that $u^1,u^2$ are regularised solutions of (1.1) in the class $\mathscr {U}^\beta$.

For $(s,a) \in [0,1]_{\le }^2$, consider the $(C(\mathbb {T}))^4$-valued $\mathscr {F}_a$-measurable random variable

6.9 $$\begin{aligned} Z :=\Big (\phi ^{u^1(s,\cdot ),s}(a,\cdot ),\ \phi ^{u^2(s,\cdot ),s}(a,\cdot ),\ u^1(a,\cdot ),\ u^2(a,\cdot )\Big ). \end{aligned}$$

Recall the definitions of $F^{(2)}$ and $\Sigma ^{(2)}$ from (5.7) and (5.8). Moreover for $(t,x) \in [0,1]\times \mathbb {T}$ and $z = (z_1,\dots , z_4) \in (C(\mathbb {T}))^4$, define6.106.11By Lemma 5.6, it follows that the expression in (6.10) is continuous in *z*. Similarly, by Lemma 5.6, the product and chain rule formulas for Malliavin derivatives, it is easy to see that the same holds for the expression in (6.11). Our next task is to obtain an estimate on $F^{(4)}$ evaluated at *Z*. This estimate is given in the next lemma.

### Lemma 6.6

(Four point estimate) Let Assumption 6.5 hold. Then for all $p \in [2,\infty )$, there exists a constant $N = N(n,p,\Vert \sigma \Vert _{C^{n+2}}, \alpha , \beta )$ such that for all $(S,T) \in [0,1]_{\le }^2$, $(s,a) \in [S,T]_{\le }^2$, t∈[0,1-a+s] and $x \in \mathbb {T}$, we have that

$$\begin{aligned} \Vert F^{(4)}_{p,n}(t,\,x,Z)\Vert _{L_{p}} \le \,&N (1+\max _{i \in \{1,2\}}[D^{u^i}]_{\mathscr {V}_{2p}^\beta })\Big ([u^1,u^2]_{\mathscr {S}_p^{1/2}[S,a]} \\&+ \Vert u^1(S,\cdot ) - u^2(S,\cdot )\Vert _{\mathbb {B}(\mathbb {T}, L_p)}\Big )|a-s|^{\frac{1}{2}}, \end{aligned}$$

where *Z* is defined by (6.9).

To prove the above estimate, we will need the following auxiliary lemma.

### Lemma 6.7

Let Assumption 6.5 hold. Then for all $p \in [2,\infty )$ there exists a constant $N = N(n,p, \Vert \sigma \Vert _{C^{n+2}}, \alpha , \beta )$ such that for all $(s,a) \in [0,1]_{\le }^2$, t∈[0,1-a+s] and $x \in \mathbb {T}$ we have

$$\begin{aligned}\Vert \Sigma ^{(4)}_{p,n}(t,x,Z)\Vert _{L_{p}}&\le N \max _{i \in \{1,2\}}[D^{u^i}]_{\mathscr {V}_{2p}^\beta }\Vert u^1(s,\cdot ) - u^2(s,\cdot )\Vert _{\mathbb {B}(\mathbb {T}, L_p)} (a-s)^{\beta }\\&\qquad + N\textbf{1}_{n \ge 1}\sum _{i=0}^{n-1} \Vert F^{(4)}_{2p,i}(t,x,Z)\Vert _{L_{p}} + N\Vert F_{p,n}^{(4)}(t,x, Z)\Vert _{L_{p}}. \end{aligned}$$

### Proof

We consider first the case when n≥1. By Lemma 4.4 (c) (which we can apply with $\varepsilon = t^{1/4} \in [0,1]$ by Lemma 5.1) and by the triangle inequality and Hölder’s inequality we have that

$$\begin{aligned}&\Vert \Sigma _{p,n}^{(4)}(t,x,Z)\Vert _{L_p|\mathscr {F}_s}\\&\lesssim \sum _{i+j \le n} \big \Vert F_{4p,i}^{(2)}(t,x,Z_1,Z_2)\big \Vert _{L_{2p}|\mathscr {F}_s} \Big ( \sum _{l \in \{1,2\}}\big \Vert F_{4p,j}^{(2)}(t,x, Z_l, Z_{l+2}) \big \Vert _{L_{2p}|\mathscr {F}_s} \Big )\\&\qquad + \sum _{i=0}^{n-1} \big \Vert F^{(4)}_{2p,i}(t,x,Z)\big \Vert _{L_p|\mathscr {F}_s} + \Vert F_{p,n}^{(4)}(t,x,Z)\Vert _{L_p|\mathscr {F}_s}\\&=: A+B+C. \end{aligned}$$

We can immediately see that $\Vert B\Vert _{L_p}, \Vert C\Vert _{L_p}$ can be estimated by the second and third terms of the desired bound. We proceed with showing that $\Vert A\Vert _{L_p}$ can be estimated by the first term of the desired bound. To this end, note that by Lemmata 5.6 and 6.4 we have for l=1,2, uniformly in $j \in \{0,\dots , n\}$, that

$$\begin{aligned} \big \Vert F^{(2)}_{4p,j}(t,x,Z_l,Z_{l+2})\big \Vert _{L_{2p,\infty }^{\mathscr {F}_s}}&\lesssim \sup _{w \in \mathbb {T}}\Vert Z_l(w) - Z_{l+2}(w)\Vert _{L_{2p,\infty }^{\mathscr {F}_s}}\\&= \sup _{w \in \mathbb {T}}\Vert \phi ^{u^l(s,\cdot ),s}(a,w) - u^l(a,w)\Vert _{L_{2p,\infty }^{\mathscr {F}_s}}\\&\lesssim (a-s)^{\beta }\max _{l \in \{1,2\}}[D^{u^l}]_{\mathscr {V}_{2p}^\beta [s,a]}. \end{aligned}$$

Therefore

6.12 $$\begin{aligned} A \lesssim (a-s)^{\beta }\max _{l \in \{1,2\}}[D^{u^l}]_{\mathscr {V}_{2p}^\beta }\sum _{i+j \le n} \Vert F^{(2)}_{4p,i}(t,x,Z_1,Z_2) \Vert _{L_{2p}|\mathscr {F}_s} . \end{aligned}$$

Applying Lemma 5.6 with q=4p, $(p_1,p_2) = (2p,p)$, $\mathscr {G} = \mathscr {F}_s$, we get

$$\begin{aligned} \Vert F_{4p,i}^{(2)}(t,x,Z_1,Z_2)\Vert _{L_{2p, p}^{\mathscr {F}_s}}&\lesssim t^{i/4}\sup _{w \in \mathbb {T}}\Vert Z_1(w) - {Z}_2(w)\Vert _{L_{2p,p}^{\mathscr {F}_s}}\\&= t^{i/4}\sup _{w \in \mathbb {T}}\Vert \phi ^{u^1(s,\cdot ), s}(a,w) - \phi ^{u^2(s,\cdot ), s}(a,w)\Vert _{L_{2p,p}^{\mathscr {F}_s}}\\&= t^{i/4}\sup _{w \in \mathbb {T}}\Vert F^{(2)}_{2p, 0}(a-s, w, u^1(s,\cdot ), u^2(s,\cdot ))\Vert _{L_p}, \end{aligned}$$

for all $i \in \{0,\dots ,n\}$, where the last equality holds by Lemma 11.11. So applying Lemma 5.6 with q=2p, $p_1 = p_2 = p$, and with an arbitrary sub-σ-algebra $\mathscr {G}\subset \mathscr {F}$, we get from the above inequality that

$$\begin{aligned} \sup _i\Vert F_{4p,i}^{(2)}(t,x,Z_1,Z_2)\Vert _{L_{2p,p}^{\mathscr {F}_s}} \lesssim \sup _{w \in \mathbb {T}}\Vert u^1(s,w) - u^2(s,w)\Vert _{L_p}. \end{aligned}$$

Now taking the $L_p$-norm on (6.12), and applying the above inequality, we get that

$$\begin{aligned} \Vert A\Vert _{L_p}&\lesssim (a-s)^{\beta }\max _{l \in \{1,2\}}[D^{u^l}]_{\mathscr {V}_{2p}^\beta }\sum _{i+j\le n} \Vert F_{4p,i}^{(2)}(t,x,Z_1,Z_2)\Vert _{L_{2p,p}^{\mathscr {F}_s}} \\  &\lesssim (a-s)^{\beta }\max _{l \in \{1,2\}}[D^{u^l}]_{\mathscr {V}_{2p}^\beta }\sup _{w \in \mathbb {T}}\Vert u^1(s,w) - u^2(s,w)\Vert _{L_p}, \end{aligned}$$

which finishes the proof for the case when n≥1.

Finally, when n=0, using (11.12), we have

$$\begin{aligned} \Vert \Sigma _{p,n}^{(4)}(t,x,Z)\Vert _{L_p|\mathscr {F}_s}&\le \Vert F_{2p,n}^{(4)}(t,x,Z_1,Z_2)\Vert _{L_{2p}|\mathscr {F}_s}\\&\qquad \sum _{l \in \{1,2\}}\Vert F_{2p,j}^{(2)}(t,x,Z_{l}, Z_{l+2})\Vert _{L_{2p}|\mathscr {F}_s}\\&\qquad + \Vert F_{p,0}^{(4)}(t,x,Z)\Vert _{L_p|\mathscr {F}_s}\\&= : \tilde{A} + \tilde{C}. \end{aligned}$$

By estimating $\Vert \tilde{A}\Vert _{L_p}$ and $\Vert \tilde{C}\Vert _{L_p}$ the same way as we did for $\Vert A\Vert _{L_p}$ and $\Vert C\Vert _{L_p}$ respectively, one can show that the desired result also holds for n=0, which finishes the proof. □

We are now in position to prove Lemma 6.6.

### Proof of Lemma 6.6

We begin by confirming that $\sup _{(t,x) \in [0,1]\times \mathbb {T}}\Vert F^{(4)}_{p,n}(t,x,Z)\Vert _{L_{p}} < \infty$. This is indeed true, since by the triangle inequality, Lemma 5.6 and Lemma 6.4 we have

6.13 $$\begin{aligned} \sup _{x \in \mathbb {T}}\Vert F^{(4)}_{p,n}(t,x,Z)\Vert _{L_{p}}&\le \sum _{i \in \{1,2\}}\Vert F_{p,n}^{(2)}\big (t,x, \phi ^{u^i(s,\cdot ),s}(a,\cdot ), u^i(a,\cdot )\big )\Vert _{L_{p}} \nonumber \\&\lesssim \sum _{i \in \{1,2\}}t^{\frac{n}{4}}\sup _{x \in \mathbb {T}}\Vert \phi ^{u^i(s,\cdot ),s}(a,x)- u^i(a,x)\Vert _{L_{p}} \nonumber \\  &\lesssim t^{\frac{n}{4}}(a-s)^{\beta }\max _{i \in \{1,2\}}[D^{u^i}]_{\mathscr {V}_p^\beta }. \end{aligned}$$

The rest of the proof will be done by induction.

Step 1: We prove that the statement holds for n=0. By (5.30) we have for $z=(z_1,...,z_4) \in (C(\mathbb {T}))^4$ that

$$\begin{aligned}&(\phi ^{z_1} - \phi ^{z_2} - \phi ^{z_3} + \phi ^{z_4})(t,x)= P_{t}(z_1 -z_2 -z_3 + z_4)(x)\\&\qquad + \int _0^{t}\int _{\mathbb {T}} p_{t-r}(x,y)\big (\sigma (\phi ^{z_1}(r,y)) -\sigma (\phi ^{z_2}(r,y)) \\&\qquad - \sigma (\phi ^{z_3}(r,y))+\sigma (\phi ^{z_4}(r,y))\big )\xi (dy,dr). \end{aligned}$$

Therefore by the BDG inequality

6.14 $$\begin{aligned}&F_{p,0}^{(4)}(t,x,z) \nonumber \\&\lesssim P_t(z_1 - z_2 - z_3 + z_4)(x)+ \Big (\int _0^{t}\int _{\mathbb {T}}p_{t-r}^2(x,y)\big |\Sigma _{p,0}^{(4)}(r,y,z)\big |^2 dydr\Big )^{1/2} \nonumber \\&=: \bar{A}(z) + \bar{B}(z). \end{aligned}$$

By the definition of the $\mathscr {S}_p^{1/2}$-bracket, we have

$$\begin{aligned} \Vert \bar{A}(Z)\Vert _{L_{p}}&\le \sup _{x \in \mathbb {T}}\Vert \phi ^{u^1(s,\cdot ),s}(a,x) - \phi ^{u^2(s,\cdot ),s}(a,x) - u^1(a,x) + u^2(a,x)\Vert _{L_p}\\&\lesssim [u^1,u^2]_{\mathscr {S}_p^{1/2}[S,a]}(a-s)^{1/2}. \end{aligned}$$

Moreover using Lemm 6.7, one can show that

$$\begin{aligned} \Vert \bar{B}(Z)\Vert _{L_{p}}&\lesssim \max _{i \in \{1,2\}}[D^{u^i}]_{\mathscr {V}_{2p}^\beta }\sup _{x \in \mathbb {T}}\Vert u^1(s,x) - u^2(s,x)\Vert _{L_p}(a-s)^{\beta } \\&\qquad + \Big (\int _0^{t}\int _{\mathbb {T}}p_{t-r}^2(x,y)\Vert F_{p,0}^{(4)}(r,y,Z)\Vert _{L_{p}}^2dydr\Big )^{1/2}. \end{aligned}$$

Using the above bounds on $\bar{A}, \bar{B}$, the decomposition (6.14), the assumption that $\beta \ge 1/2$ and Lemm 6.3, we can see that

$$\begin{aligned}&\Vert F^{(4)}_{p,0}\big (t,x,Z\big )\Vert _{L_{p}}^2 \\&\quad \lesssim \Big |(1+\max _{i \in \{1,2\}}[D^{u^i}]_{\mathscr {V}_{2p}^\beta })\big ([u^1,u^2]_{\mathscr {S}_p^{1/2}[S,a]} + \Vert u^1(S,\cdot ) - u^2(S,\cdot )\Vert _{\mathbb {B}(\mathbb {T}, L_p)}\big )(a-s)^{1/2}\Big |^2\\&\qquad + \int _0^{t}\int _{\mathbb {T}}p_{t-r}^2(x,y)\Vert F^{(4)}_{p,0}(r,y,Z)\Vert _{L_{p}}^2dydr. \end{aligned}$$

Since by assumption $u^1,u^2 \in \mathscr {U}_{2p}^\beta$, we have $\max _{i \in \{1,2\}}[D^{u^i}]_{\mathscr {V}_{2p}^\beta } <\infty$. Therefore by (6.13) we have that the norm in the integrand is bounded in (*r*, *y*). Hence, an application of Lemma 11.4 yields the result for the n=0 case.

Step 2: Let $n \in \mathbb {N}$ and suppose that the statement holds for $F_{p,0}^{(4)},\dots , F^{(4)}_{p,n-1}$ for all p≥2. We aim to show that then the result also holds for $F^{(4)}_{p,n}$ for all p≥2. Let $\gamma = (\theta _i,\zeta _i)_{i=1}^n\in ([0,1]\times \mathbb {T})^n$ and $z=(z_1,...,z_4) \in (C(\mathbb {T}))^4$. Then by Proposition 5.3 we have that

$$\begin{aligned}&\mathscr {D}_\gamma ^n(\phi ^{z_1} - \phi ^{z_2} - \phi ^{z_3} + \phi ^{z_4})(t,x)\\&= \textbf{1}_{[0,t]}(\theta ^*)\sum _{k=1}^n p_{t-\theta _k}(x,\zeta _k)\\&\qquad \qquad \times \mathscr {D}_{\hat{\gamma }_k}^{n-1}\big [ \sigma (\phi ^{z_1}(\theta _k,\zeta _k)) -\sigma (\phi ^{z_2}(\theta _k,\zeta _k))-\sigma (\phi ^{z_3}(\theta _k,\zeta _k)) +\sigma (\phi ^{z_4}(\theta _k,\zeta _k))\big ]\\&+ \textbf{1}_{[0,t]}(\theta ^*)\int _{0}^{t}\int _{\mathbb {T}} p_{t-r}(x,y)\\&\qquad \qquad \times \mathscr {D}_\gamma ^n\big [\sigma (\phi ^{z_1}(r,y)) -\sigma (\phi ^{z_2}(r,y)) - \sigma (\phi ^{z_3}(r,y)) + \sigma (\phi ^{z_4}(r,y)) \big ]\xi (dy,dr). \end{aligned}$$

Taking the $\Vert \cdot \Vert _{L_p(\Omega ;H^{\otimes n})}$-norm of both sides and using the BDG inequality gives

6.15 $$\begin{aligned}&F^{(4)}_{p,n}(t,x,z) \nonumber \\&\quad \lesssim \Vert \textbf{1}_{[0,t]}(\cdot )p_{t-\cdot }(x,\cdot ) \mathscr {D}^{n-1}[\sigma (\phi ^{z_1}(\cdot ,\cdot )) - \sigma (\phi ^{z_2}(\cdot ,\cdot )) \nonumber \\&\qquad - \sigma (\phi ^{z_3}(\cdot ,\cdot ))+ \sigma (\phi ^{z_4}(\cdot ,\cdot ))]\Vert _{L_p(\Omega ;H^{\otimes n})} \nonumber \\&\qquad + \Big ( \int _0^t\int _{\mathbb {T}} p_{t-r}^2(x,y)|\Sigma _{p,n}^{(4)}(r,y,z)|^2dydr \Big )^{1/2} \nonumber \\&\quad =: A(z)+B(z). \end{aligned}$$

We begin by bounding *A*. Note that

$$\begin{aligned} A(z) \lesssim \,&\Vert \textbf{1}_{[0,t]}(\cdot )p_{t-\cdot }(x,\cdot )\Vert \Vert \mathscr {D}^{n-1}[\sigma (\phi ^{z_1}(\cdot ,\cdot )) - \sigma (\phi ^{z_2}(\cdot ,\cdot )) \\  &- \sigma (\phi ^{z_3}(\cdot ,\cdot ))+ \sigma (\phi ^{z_4}(\cdot ,\cdot ))]\Vert _{H^{\otimes (n-1)}}\Vert _{L_p}\Vert _{H}\\ =\,&\Vert \textbf{1}_{[0,t]}(\cdot )p_{t-\cdot }(x,\cdot )\Sigma ^{(4)}_{p,n-1}(\cdot ,\cdot ,z)\Vert _H, \end{aligned}$$

and thus (recalling the definition of *Z* from (6.9)) we have $A(Z) \lesssim \Vert \textbf{1}_{[0,t]}(\cdot )p_{t-\cdot }(x,\cdot )\Sigma ^{(4)}_{p,n-1}(\cdot ,\cdot ,Z)\Vert _H$. Hence using the Minkowski inequality we obtain that

$$\begin{aligned} \Vert A(Z)\Vert _{L_{p}}&\lesssim \Vert \textbf{1}_{[0,t](\cdot )} p_{t-\cdot }(x,\cdot ) \Vert \Sigma ^{(4)}_{p,n-1}(\cdot ,\cdot ,Z)\Vert _{L_{p}}\Vert _H\\&\le \Vert \textbf{1}_{[0,t]}(\cdot )p_{t-\cdot }(x,\cdot )\Vert _{H}\sup _{(\theta ,\zeta ) \in [0,t]\times \mathbb {T}}\Vert \Sigma ^{(4)}_{p,n-1}(\theta ,\zeta ,Z)\Vert _{L_{p}}. \end{aligned}$$

To bound the first factor, we note that $\Vert \textbf{1}_{[0,t]}(\cdot )p_{t-\cdot }(x,\cdot )\Vert _{H} \lesssim t^{1/4} \le 1$, To bound the second factor, we apply Lemmata 6.7 and 6.3, the induction hypothesis and the fact that $\beta \ge 1/2$ to get that for $(\theta ,\zeta ) \in [0,t]\times \mathbb {T}$,

$$\begin{aligned}&\Vert \Sigma ^{(4)}_{p,n-1}(\theta ,\zeta ,Z)\Vert _{L_{p}}\\&\lesssim \max _{i \in \{1,2\}}[D^{u^i}]_{\mathscr {V}_{2p}^\beta }\Vert u^1(s,\cdot ) - u^2(s,\cdot )\Vert _{\mathbb {B}(\mathbb {T}, L_p)} (a-s)^{\beta } + \sum _{i=0}^{n-1} \Vert F^{(4)}_{2p,i}(\theta ,\zeta ,Z)\Vert _{L_{p}} \\&\lesssim (1+ \max _{i \in \{1,2\}}[D^{u^i}]_{\mathscr {V}_{2p}^\beta })\Big ([u^1,u^2]_{\mathscr {S}_p^{1/2}[S,a]} + \Vert u^1(S,\cdot ) - u^2(S,\cdot )\Vert _{\mathbb {B}(\mathbb {T}, L_p)}\Big ) (a-s)^{\frac{1}{2}}. \end{aligned}$$

Hence we can see that

$$\begin{aligned}\Vert A(Z)\Vert _{L_{p}} \lesssim \,&(1+ \max _{i \in \{1,2\}}[D^{u^i}]_{\mathscr {V}_{2p}^\beta })\\&\Big ([u^1,u^2]_{\mathscr {S}_p^{1/2}[S,a]} + \Vert u^1(S,\cdot ) - u^2(S,\cdot )\Vert _{\mathbb {B}(\mathbb {T}, L_p)}\Big )(a-s)^{\frac{1}{2}}.\end{aligned}$$

We proceed with bounding *B*. Note that

6.16 $$\begin{aligned} \Vert B(Z)\Vert _{L_{p}} \lesssim \Big (\int _{0}^{t} \int _{\mathbb {T}} p_{t-r}^2(x,y) \Vert \Sigma _{p,n}^{(4)}(r,y,Z)\Vert _{L_p}^2 dydr \Big )^{1/2}. \end{aligned}$$

By Lemma 6.7, that $\beta \ge 1/2$ and the induction hypothesis we have for all $(r,y) \in [0,t]\times \mathbb {T}$ that

$$\begin{aligned}&\Vert \Sigma ^{(4)}_{p,n}(r,y,Z)\Vert _{L_{p}}\\&\quad \lesssim \max _{i \in \{1,2\}}[D^{u^i}]_{\mathscr {V}_{2p}^\beta }\Vert u^1(s,\cdot ) - u^2(s,\cdot )\Vert _{\mathbb {B}(\mathbb {T}, L_p)} (a-s)^{\beta }\\&\qquad + \sum _{i=0}^{n-1} \Vert F^{(4)}_{2p,i}(r,y,Z)\Vert _{L_{p}} + \Vert F^{(4)}_{p,n}(r,y,Z)\Vert _{L_{p}}\\&\quad \lesssim (1+ \max _{i \in \{1,2\}}[D^{u^i}]_{\mathscr {V}_{2p}^\beta })\Big ([u^1,u^2]_{\mathscr {S}_p^{1/2}[S,a]} + \Vert u^1(S,\cdot ) - u^2(S,\cdot )\Vert _{\mathbb {B}(\mathbb {T}, L_p)}\Big ) (a-s)^{1/2}\\&\qquad +\Vert F_{p,n}^{(4)}(r,y,Z)\Vert _{L_{p}}. \end{aligned}$$

Putting this into (6.16), we can see that

$$\begin{aligned}&\Vert B(Z)\Vert _{L_{p} }\\&\lesssim (1+ \max _{i \in \{1,2\}}[D^{u^i}]_{\mathscr {V}_{2p}^\beta })\Big ([u^1,u^2]_{\mathscr {S}_p^{1/2}[S,a]} + \Vert u^1(S,\cdot ) - u^2(S,\cdot )\Vert _{\mathbb {B}(\mathbb {T}, L_p)}\Big ) (a-s)^{1/2}\\&\qquad + \Big (\int _{0}^{t}\int _{\mathbb {T}} p_{t-r}^2(x,y)\Vert F^{(4)}_{p,n}(r,y,Z)\Vert _{L_{p}}^2 dydr\Big )^{1/2}. \end{aligned}$$

By our bounds on *A*, *B*, we may conclude that

$$\begin{aligned}&\Vert F^{(4)}_{p,n}(t,x,Z)\Vert _{L_{p}}\\&\lesssim (1+ \max _{i \in \{1,2\}}[D^{u^i}]_{\mathscr {V}_{2p}^\beta })\Big ([u^1,u^2]_{\mathscr {S}_p^{1/2}[S,a]} + \Vert u^1(S,\cdot ) - u^2(S,\cdot )\Vert _{\mathbb {B}(\mathbb {T}, L_p)}\Big )(a-s)^{1/2} \\&\qquad + \Big (\int _{0}^{t}\int _{\mathbb {T}} p_{t-r}^2(x,y)\Vert F^{(4)}_{p,n}(r,y,Z)\Vert _{L_{p}}^2 dydr\Big )^{1/2}. \end{aligned}$$

By (6.13) the norm in the integrand is bounded in (*r*, *y*). Hence, we can apply Lemma 11.4 to conclude the proof. □

### Lemma 6.8

(Four point BDG inequality for driftless approximations) Let $p \in [2,\infty )$, $\sigma \in C^2$, $\alpha \in (-1,0)$, $\beta \in [0, 1+\alpha /4]$ and for i=1,2, let $b^i \in C^\alpha$ and suppose that $u^i \in \mathscr {U}^\beta$ are regularised solutions of

$$(\partial _t - \Delta )u^i = b^i(u^i) + \sigma (u^i)\xi (dy,dr).$$

There exists a constant $N = N(p, \Vert \sigma \Vert _{C^2}, \varepsilon , \alpha , \beta )$ such that for $(s,t) \in [0,1]_{\le }^2$ we have

$$\begin{aligned}&\Big \Vert \int _s^t \int _{\mathbb {T}} p_{t-r}(x,y)\\&\qquad \times \Big (\sigma (u^1(r,y)) - \sigma (u^2(r,y))- \sigma (\phi ^{u^1(s,\cdot ),s}(r,y)) + \sigma (\phi ^{u^2(s,\cdot ),s}(r,y))\Big ) \xi (dy,dr)\Big \Vert _{L_p} \\&\quad \le N[D^{u^1}]_{\mathscr {V}_{2p}^\beta } \Vert u^1(s,\cdot ) -u^2(s,\cdot )\Vert _{\mathbb {B}(\mathbb {T}, L_p)}(t-s)^{ \frac{1}{4}+\beta }\\&\qquad + N\Big (\int _s^t\int _{\mathbb {T}} p_{t-r}^2(x,y)\big \Vert u^1(r,y) - u^2(r,y) - \phi ^{u^1(s,\cdot ),s}(r,y) + \phi ^{u^2(s,\cdot ),s}(r,y)\big \Vert _{L_p}^2 dydr \Big )^{1/2}. \end{aligned}$$

### Proof

By the BDG inequality and by (11.13) in Lemma 11.7 we have

$$\begin{aligned}&\Big \Vert \int _s^t \int _{\mathbb {T}} p_{t-r}(x,y)\Big (\sigma (u^1(r,y))- \sigma (u^2(r,y)) \\&\qquad \qquad \qquad \qquad -\sigma (\phi ^{u^1(s,\cdot ),s}(r,y)) + \sigma (\phi ^{u^2(s,\cdot ),s}(r,y))\Big ) \xi (dy,dr)\Big \Vert _{L_p}^2\\&\lesssim \int _s^t \int _{\mathbb {T}}p_{t-r}^2(x,y)\Big \Vert \sigma (u^1(r,y))- \sigma (u^2(r,y))\\&\qquad \qquad \qquad \qquad - \sigma (\phi ^{u^1(s,\cdot ),s}(r,y)) + \sigma (\phi ^{u^2(s,\cdot ),s}(r,y))\Big \Vert _{L_p}^2 dydr\\&\lesssim I+J \end{aligned}$$

with

$$\begin{aligned} I&:= \int _s^t \int _{\mathbb {T}} p_{t-r}^2(x,y)\Big \Vert \Big (\phi ^{u^1(s,\cdot ),s}(r,y) - \phi ^{u^2(s,\cdot ),s}(r,y)\Big )\\&\qquad \Big (\phi ^{u^1(s,\cdot ),s}(r,y) - u^1(r,y)\Big )\Big \Vert _{L_p}^2dydr\\ J&:= \int _s^t \int _{\mathbb {T}} p_{t-r}^2(x,y)\Vert u^1(r,y) - u^2(r,y) - \phi ^{u^1(s,\cdot ),s}(r,y) \\&\qquad + \phi ^{u^2(s,\cdot ),s}(r,y)\Vert _{L_p}^2 dydr. \end{aligned}$$

By the tower rule, Hölder’s inequality, Lemma 6.4, Lemma 11.11 and Lemma 5.6 (where we recall the definition (5.7) of $F^{(2)}$), we can see that

$$\begin{aligned}&\Big \Vert \Big (\phi ^{u^1(s,\cdot ),s}(r,y) - \phi ^{u^2(s,\cdot ),s}(r,y)\Big )\Big (\phi ^{u^1(s,\cdot ),s}(r,y) - u^1(r,y)\Big )\Big \Vert _{L_p} \\&\quad \le \Big \Vert \big \Vert \phi ^{u^1(s,\cdot ),s}(r,y) - \phi ^{u^2(s,\cdot ),s}(r,y)\big \Vert _{L_{2p}|\mathscr {F}_s}\big \Vert \phi ^{u^1(s,\cdot ),s}(r,y) - u^1(r,y)\big \Vert _{L_{2p}|\mathscr {F}_s} \Big \Vert _{L_p}\\&\quad \lesssim [D^{u^1}]_{\mathscr {V}_{2p }^\beta }(t-s)^{\beta } \Big \Vert \big \Vert \phi ^{u^1(s,\cdot ),s}(r,y) - \phi ^{u^2(s,\cdot ),s}(r,y)\big \Vert _{L_{2p}|\mathscr {F}_s}\Big \Vert _{L_p}\\&\quad = [D^{u^1}]_{\mathscr {V}_{2p}^\beta }(t-s)^{\beta }\Big \Vert F^{(2)}_{2p,0}\big (r-s,y, u^1(s,\cdot ), u^2(s,\cdot )\big )\Big \Vert _{L_p}\\&\quad \lesssim [D^{u^1}]_{\mathscr {V}_{2p}^\beta }(t-s)^{\beta }\Vert u^1(s,\cdot ) - u^2(s,\cdot )\Vert _{\mathbb {B}(\mathbb {T},L_p)}. \end{aligned}$$

Putting this bound into the definition of *I*, we get that

$$\begin{aligned} I&\lesssim [D^{u^1}]_{\mathscr {V}_{2p}^\beta }^2(t-s)^{2\beta }\Vert u^1(s,\cdot ) - u^2(s,\cdot )\Vert _{C(\mathbb {T},L_p)}^2\int _s^t\int _{\mathbb {T}}p_{t-r}^2(x,y)dydr\\&\lesssim [D^{u^1}]_{\mathscr {V}_{2p}^\beta }^2(t-s)^{2\beta + \frac{1}{2}}\Vert u^1(s,\cdot ) - u^2(s,\cdot )\Vert _{\mathbb {B}(\mathbb {T},L_p)}^2. \end{aligned}$$

This bound on *I* and the definition of *J* together give the desired bound. □

## Regularisation estimates

Let $u,u^1,u^2$ be regularised solutions of (1.1) with potentially different drift terms, *f* be a measurable kernel on $(0,1]\times \mathbb {T}$ and *g* be a smooth function on ${\mathbb {R}}$. In this section, we obtain quantitative bounds for expressions of the forms $\int _s^t \int _{\mathbb {T}} f_r(y)g(u(r,y))dydr$ and $\int _s^t \int _{\mathbb {T}}f_r(y)(g(u^1(r,y))- g(u^2(r,y)))dydr$ which depend on a Besov–Hölder norm of *g* with a negative index.

### Lemma 7.1

Let Assumption 3.1 hold and let *u* be a regularised solution of (1.1). Suppose that $f:(0,1]\times \mathbb {T} \rightarrow \mathbb {R}$ is a measurable function such that there exist constants K>0 and $\zeta \in [0,\frac{1}{4}]$ such that for all t∈(0,1] it holds that

7.1 $$\begin{aligned} \int _{\mathbb {T}}|f_t(y)|dy \le Kt^{-\zeta }. \end{aligned}$$

Let $p \in [1,\infty )$. For all $\lambda \in \big (4\zeta -2,-1\big )\cup (-1,0)$ and for all β in the nonempty set $(\frac{1}{4} - \frac{\lambda }{4} + \zeta , 1 + \frac{\alpha }{4}]$, if $u \in \mathscr {U}_2^\beta$ then there exists a constant $N = N(p,\Vert \sigma \Vert _{C^2}, \mu , \lambda , \alpha , \beta , \zeta )$, such that for all $g \in C^\infty$, $(s,t) \in [0,1]_{\le }^2$ and $\mathscr {G} \in \{\mathscr {F}_s, \{\emptyset , \Omega \}\}$ we have

$$\begin{aligned}&\Big \Vert \int _s^t \int _{\mathbb {T}}f_{t-r}(y)g(u(r,y))dydr \Big \Vert _{L_{p,\infty }^\mathscr {G}}\\&\quad \le N\Vert g\Vert _{C^\lambda }K\Big ((t-s)^{1 + \frac{\lambda }{4} -\zeta } + [D^u]_{\mathscr {V}_2^\beta [s,t]}(t-s)^{\beta +\frac{\lambda + 3}{4}- \zeta } \Big ). \end{aligned}$$

### Proof

We may assume without loss of generality that p>2 and K=1. For $(S,T) \in [0,1]_{\le }^2$ and $(s,t) \in [S,T]_{\le }^2$ we consider the germ

$$\begin{aligned}A_{s,t} := \mathbb {E}^s\int _s^t \int _{\mathbb {T}} f_{T-r}(y)g(\phi ^{u(s,\cdot ),s}(r,y))dydr. \end{aligned}$$

We have

$$\begin{aligned} |A_{s,t}|&\le \int _s^t \int _{\mathbb {T}} |f_{T-r}(y)||\mathbb {E}^s g(\phi ^{u(s,\cdot ),s}(r,y))|dydr. \end{aligned}$$

Note that by Lemma 5.11 (with n=0) we have $|\mathbb {E}^sg(\phi ^{u(s,\cdot ),s}(r,y))| \lesssim \Vert g\Vert _{C^\lambda }(r-s)^{\lambda /4}$. Using this estimate and (7.1), it follows that

$$\begin{aligned} \Vert A_{s,t}\Vert _{L_{p,\infty }^{\mathscr {G}}} \lesssim \Vert g\Vert _{C^\lambda }\int _s^t (r-s)^{\lambda /4} \int _{\mathbb {T}}|f_{T-r}(y)|dydr \lesssim \Vert g\Vert _{C^\lambda }(t-s)^{1 + \lambda /4 - \zeta }. \end{aligned}$$

From the assumption that $\lambda > 4\zeta -2$ it follows that the exponent $1+\lambda /4-\zeta$ is greater than 1/2, and thus the first condition in (4.1) is satisfied. Let a∈[s,t]. Then

$$\begin{aligned} |\mathbb {E}^s \delta A_{s,a,t}|&= |\mathbb {E}^s(A_{s,t} - A_{s,a} - A_{a,t})|\\&= \Big |\mathbb {E}^s \int _a^t \int _{\mathbb {T}} f_{T-r}(y)\mathbb {E}^a\Big (g(\phi ^{u(s,\cdot ),s}(r,y)) - g(\phi ^{u(a,\cdot ),a}(r,y))\Big )dydr\Big |. \end{aligned}$$

By the Fundamental Theorem of Calculus and Lemma 5.11 (with n=1), we get that

$$\begin{aligned}&\Big |\mathbb {E}^a\Big (g(\phi ^{u(s,\cdot ),s}(r,y)) - g(\phi ^{u(a,\cdot ),a}(r,y))\Big )\Big |\\&\quad = \Big | \int _0^1 \mathbb {E}^a\Big (\nabla g\Big (\theta \phi ^{u(s,\cdot ),s}(r,y) + (1-\theta ) \phi ^{u(a,\cdot ),a}(r,y)\Big ) \\&\quad \quad \Big (\phi ^{u(s,\cdot ),s}(r,y) - \phi ^{u(a,\cdot ),a}(r,y)\Big )\Big )d\theta \Big |\\&\quad = \Big | \int _0^1 \mathbb {E}^a\Big (\nabla g\Big (\theta \phi ^{\phi ^{u(s,\cdot ),s}(a,\cdot ),\,a}(r,y) + (1-\theta ) \phi ^{u(a,\cdot ),a}(r,y)\Big )\\&\qquad \times \Big (\phi ^{\phi ^{u(s,\cdot ),s}(a,\cdot ),\,a}(r,y) - \phi ^{u(a,\cdot ),a}(r,y)\Big )\Big )d\theta \Big |\\&\quad \lesssim \Vert g\Vert _{C^\lambda }(r-a)^{(\lambda -1)/4}\sum _{i=0}^3F_{16,i}^{(2)}(r-a,y,\phi ^{u(s,\cdot ),s}(a,\cdot ), u(a,\cdot )), \end{aligned}$$

where $F^{(2)}$ is defined by (5.7). Therefore using the above result, Lemma 5.6 and Lemma 6.4, we get

$$\begin{aligned}&\mathbb {E}^s\Big |\mathbb {E}^a\Big (g(\phi ^{u(s,\cdot ),s}(r,y)) - g(\phi ^{u(a,\cdot ),a}(r,y))\Big )\Big |\\  &\qquad \lesssim \Vert g\Vert _{C^\lambda }(r-a)^{ (\lambda -1)/4}\sum _{i=0}^3\mathbb {E}^s F_{16,i}^{(2)}\big (r-a,y,\phi ^{u(s,\cdot ),s}(a,\cdot ), u(a,\cdot )\big )\\&\qquad \lesssim \Vert g\Vert _{C^\lambda }(r-a)^{ (\lambda -1)/4}\sup _{x \in \mathbb {T}}\Vert \phi ^{u(s,\cdot ),s}(a,x) - u(a,x)\Vert _{L_{2,\infty }^{\mathscr {F}_s}}\\&\qquad \lesssim \Vert g\Vert _{C^\lambda }(r-a)^{(\lambda -1)/4}[D^u]_{\mathscr {V}_2^\beta [S,T]}(a-s)^{\beta }. \end{aligned}$$

Using the above inequality and (7.1), we get

$$\begin{aligned} \Vert \mathbb {E}^s \delta A_{s,a,t}\Vert _{L_\infty }&\lesssim \Vert g\Vert _{C^\lambda }[D^u]_{\mathscr {V}_2^\beta [S,T]}(a-s)^\beta \int _a^t(T-r)^{-\zeta }(r-a)^{\lambda /4 - 1/4}dr\\&\lesssim \Vert g\Vert _{C^\lambda }[D^u]_{\mathscr {V}_2^\beta [S,T]}(t-s)^{\beta - \zeta + \lambda /4 + 3/4}. \end{aligned}$$

The assumption on β ensures that the exponent $\beta - \zeta + \lambda /4 + 3/4$ is greater than 1, and thus the second condition in (4.1) is also satisfied. Let

$$\mathscr {A}_{s,t} := \int _s^t\int _{\mathbb {T}}f_{T-r}(y)g(u(r,y))dydr.$$

Using the regularity of *g* and Lemma 6.4, we can easily verify that $A-\mathscr {A}$ satisfies (4.2) and (4.3). By an application of Lemma 4.1, the conclusion follows from (4.4) and the fact that $(S,T) \in [0,1]_{\le }^2$ is arbitrary. □

### Corollary 7.2

Let Assumption 3.1 hold and let *u* be a regularised solution of (1.1) and let $p \in [1,\infty )$. Then for all $\lambda \in ( -2,-1)\cup (-1,0)$, $\beta \in (\frac{1}{4} - \frac{\lambda }{4}, 1+ \frac{\alpha }{4}]$, if $u \in \mathscr {U}_2^\beta$ then there exists a constant $N = N(p, \Vert \sigma \Vert _{C^2},\mu , \lambda , \alpha , \beta )$, such that for all $g \in C^{\infty }$, $(s,t) \in [0,1]_{\le }^2$, $x \in \mathbb {T}$, we have

$$\begin{aligned}&\Big \Vert \int _s^t \int _{\mathbb {T}} p_{t-r}(x,y) g(u(r,y))dy dr \Big \Vert _{L_{p,\infty } ^{\mathscr {F}_s}}\\&\quad \le N \Vert g\Vert _{C^\lambda }\Big ((t-s)^{1 + \lambda /4} + [D^u]_{\mathscr {V}_2^\beta [s,t]}(t-s)^{\beta + \frac{\lambda +3}{4}}\Big ). \end{aligned}$$

### Proof

Fix $x \in \mathbb {T}$ and for each $(r,y) \in (0,1]\times \mathbb {T}$ define $f_r(y) := p_{r}(x,y)$. Then *f* is a measurable function which satisfies $\int _{\mathbb {T}} f_r(y) dy =1$. Hence, applying Lemma 7.1 (with K:=1, ζ:=0), we obtain the result. □

### Corollary 7.3

Let Assumption 3.1 hold and let *u* be a regularised solution of (1.1) and let $p\in [1,\infty )$. Then for all $\lambda \in (-1,0)$ and for all $\beta \in (\frac{1}{2} - \frac{\lambda }{4}, 1+ \frac{\alpha }{4}]$, if $u \in \mathscr {U}_2^\beta$ then there exists a constant $N = N(p,\Vert \sigma \Vert _{C^2}, \mu , \lambda , \beta )$ such that for all $g \in C^{\infty }$, $(s,t) \in [0,1]_{\le }^2$ and $x \in \mathbb {T}$ we have

$$\begin{aligned}&\Big \Vert \int _s^t \int _{\mathbb {T}}\big (p_{t-r}(x,y) - p_{t-r}(\bar{x},y)\big ) g(u(r,y))dydr \Big \Vert _{L_{p,\infty }^{\mathscr {F}_s}}\\&\qquad \le N\Vert g\Vert _{C^\lambda }(1+[D^u]_{\mathscr {V}_2^\beta })|x-\bar{x}|^{1/2}.\end{aligned}$$

### Proof

Fix $x,\bar{x} \in \mathbb {T}$, and for each $(r,y) \in (0,1]\times \mathbb {T}$, define $f_r(y) := p_{r}(x,y) - p_{r}(\bar{x},y)$. Then by (11.3), we have $\int _{\mathbb {T}}f_r(y)dy \le C|x-\bar{x}|^{1/2}r^{-1/4}$ for some constant positive *C*. Hence applying Lemma 7.1 (with $K: = C|x-\bar{x}|^{1/2}$ and ζ:=1/4), we obtain the stated estimate. □

### Lemma 7.4

Let $p\in [2,\infty )$, $\alpha \in (-1,0)$, and let $\sigma \in C^4$ such that there exists constant μ>0 such that for all $x \in \mathbb {R}$ we have $\sigma ^2(x) \ge \mu ^2$. For i=1,2, let $b^i \in C^{\alpha }$ and let $u^i$ be regularised solutions of

$$(\partial _t - \Delta )u^i = b^i(u^i) + \sigma (u^i)\xi (dy,dr)$$

in the class $\mathscr {U}^\beta$ for some $\beta \in (\frac{1}{2} - \frac{\alpha }{4}, 1+\frac{\alpha }{4}]$. There exists a constant $N = N(p, \Vert \sigma \Vert _{C^4}, \mu , \alpha , \beta )$ such that for all $g \in C^\infty$, $(s,t) \in [0,1]^2_{\le }$ and $x \in \mathbb {T}$ we have

$$\begin{aligned}&\Big \Vert \int _s^t \int _{\mathbb {T}}p_{t-r}(x,y)\big (g(u^1(r,y)) - g(u^2(r,y))\big ) dydr\Big \Vert _{L_p} \\&\quad \le \, N\Vert g\Vert _{C^\alpha }(1+\max _{i \in \{1,2\}}[D^{u^i}]_{\mathscr {V}_{2p}^\beta })(t-s)^{ (3+\alpha )/4} \\&\qquad \times \Big ([u^1,u^2]_{\mathscr {S}^{1/2}_p[s,t]} +\Vert u^1(s,\cdot ) - u^2(s,\cdot )\Vert _{\mathbb {B}(\mathbb {T}, L_p)}\Big ). \end{aligned}$$

### Proof

Let $(S, T) \in [0,1]_{\le }^2$, $x \in \mathbb {T}$ and for $(s,t) \in [S, T]^2_\le$ define the germ

$$A_{s,t}(x) := \mathbb {E}^s \int _s^t \int _{\mathbb {T}} p_{T-r}(x,y)\Big (g(\phi ^{u^1(s,\cdot ),s}(r,y)) -g(\phi ^{ u^2(s,\cdot ),s}(r,y))\Big )dydr.$$

We first bound $\Vert A_{s,t}\Vert _{L_p}$. Using Lemma 5.11(with n=1 and thus q=8) and recalling the definition of $F^{(2)}$ from (5.7), we have

$$\begin{aligned}&\Big |\mathbb {E}^s\Big (g(\phi ^{u^1(s,\cdot ),s}(r,y)) - g(\phi ^{u^2(s,\cdot ),s}(r,y))\Big )\Big |\\&\quad =\Big |\int _0^1 \mathbb {E}^s\Big (\nabla g\Big (\theta \phi ^{u^1(s,\cdot ),s}(r,y) + (1-\theta )\phi ^{u^2(s,\cdot ),s}(r,y)\Big )\\&\quad \quad \Big (\phi ^{u^1(s,\cdot ),s}(r,y) - \phi ^{u^2(s,\cdot ),s}(r,y)\Big )\Big ) d\theta \Big |\\&\quad \lesssim \Vert g\Vert _{C^\alpha } (r-s)^{ (\alpha -1)/4}\sum _{i=0}^2F^{(2)}_{8,i}\big (r-s,y, u^1(s,\cdot ), u^2(s,\cdot )\big ). \end{aligned}$$

We take the $L_p$-norm on the inequality, and by Lemma 5.6 we get

$$\begin{aligned}&\Vert \mathbb {E}^s(g(\phi ^{u^1(s,\cdot ),s}(r,y)) - g(\phi ^{u^2(s,\cdot ),s}(r,y))\Vert _{L_p}\\&\qquad \lesssim \Vert g\Vert _{C^\alpha }(r-s)^{ (\alpha -1)/4}\Vert u^1(s,\cdot ) - u^2(s,\cdot )\Vert _{\mathbb {B}(\mathbb {T}, L_p)}. \end{aligned}$$

Using the definition of *A*, and the above inequality, we get

7.2 $$\begin{aligned}&\Vert A_{s,t}(x)\Vert _{L_p}\nonumber \\&\quad \lesssim \int _s^t \int _{\mathbb {T}}p_{T-r}(x,y)\Vert g\Vert _{C^\alpha }(r-s)^{ (\alpha -1)/4}\Vert u^1(s,\cdot ) - u^2(s,\cdot )\Vert _{\mathbb {B}(\mathbb {T},L_p)}dydr \nonumber \\&\quad \lesssim \Vert g\Vert _{C^\alpha }\Big ([u^1,u^2]_{\mathscr {S}_p^{1/2}[S,T]} + \Vert u^1(S,\cdot ) - u^2(S,\cdot )\Vert _{\mathbb {B}(\mathbb {T}, L_p)}\Big )(t-s)^{(3+ \alpha )/4}. \end{aligned}$$

We proceed with an estimate for $\Vert \mathbb {E}^s\delta A_{s,a,t}\Vert _{L_p}$ for a∈[s,t]. Note that

7.3 $$\begin{aligned} |\mathbb {E}^s\delta A_{s,a,t}|&= |\mathbb {E}^s(A_{s,t}- A_{s,a} - A_{a,t})| \nonumber \\&= \Big |\mathbb {E}^s \int _a^t \int _{\mathbb {T}}p_{T-r}(x,y) \mathbb {E}^a\Big (g(\phi ^{u^1(s,\cdot ),s}(r,y)) - g(\phi ^{u^2(s,\cdot ),s}(r,y)) \nonumber \\&\qquad \qquad \qquad \qquad \qquad - g(\phi ^{u^1(a,\cdot ),a}(r,y)) + g(\phi ^{u^2(a,\cdot ),a}(r,y))\Big )dydr\Big |. \end{aligned}$$

By Lemma 11.11, we have that

$$\begin{aligned}&\Big | \mathbb {E}^a\Big (g(\phi ^{u^1(s,\cdot ),s}(r,y)) - g(\phi ^{u^2(s,\cdot ),s}(r,y)) - g(\phi ^{u^1(a,\cdot ),a}(r,y)) + g(\phi ^{u^2(a,\cdot ),a}(r,y))\Big )\Big |\\&\quad \quad = \Gamma _{r,y}(Z), \end{aligned}$$

where for $(r,y) \in [0,1]\times \mathbb {T}$ and $z=(z_1,z_2,z_3,z_4) \in (C(\mathbb {T}))^4$,

7.4 $$\begin{aligned} {\Gamma }_{r,y}(z)&:=\big |\mathbb {E}\big (g(\phi ^{z_1}(r-a,y)) - g(\phi ^{z_2}(r-a,y)) \nonumber \\&\qquad - g(\phi ^{z_3}(r-a,y)) + g(\phi ^{z_4}(r-a,y))\big )\big |, \end{aligned}$$

and

$$\begin{aligned} Z=(Z_1,Z_2,Z_3,Z_4) := (\phi ^{u^1(s,\cdot ), s}(a, \cdot ), \phi ^{u^2(s,\cdot ), s}(a, \cdot ), u^1(a,\cdot ), u^2(a,\cdot )). \end{aligned}$$

We proceed with deriving an estimate for $\Gamma _{r,y}(Z)$. For brevity, we fix $(r,y) \in [s,t]\times \mathbb {T}$ and we set ${\Gamma }(z) := {\Gamma }_{r,y}(z)$, $\phi _i :=\phi ^{z_i}(r-a, y)$ and $\delta _{i,j}:= \phi _j - \phi _i$. By Lemma 11.7 we get

$$\begin{aligned}{\Gamma }(z)&\le \Big |\int _0^1 \int _0^1 \mathbb {E}\Big ( \delta _{1,2}\big (\theta \delta _{1,3} + (1-\theta )\delta _{2,4}\big ) \nabla ^2 g(\Theta _1(\theta ,\eta )) \Big )d\theta d\eta \Big |\\&\qquad + \Big | \int _0^1 \mathbb {E}\big ((\delta _{3,4} - \delta _{1,2}) \nabla g(\Theta _2(\theta )) \big ) d\theta \Big | \end{aligned}$$

where $\Theta _1,\Theta _2$ are convex combinations^2^ of $\phi _1,\dots , \phi _4$. Hence using Lemma 5.10 (with n=2 and n=1 for the first and second terms respectively) and recalling the definition of $F^{(4)}$ from (6.10) we get that

7.5 $$\begin{aligned} {\Gamma }(z)&\lesssim \Vert g\Vert _{C^{\alpha }}(r-a)^{-1/2 + \alpha /4}\int _0^1\Vert \delta _{1,2}(\theta \delta _{1,3} + (1-\theta )\delta _{2,4})\Vert _{\mathscr {W}_{16}^3}d\theta \nonumber \\  &\qquad + \Vert g\Vert _{C^\alpha }(r-a)^{-1/4 + \alpha /4}\Vert \delta _{3,4} - \delta _{1,2}\Vert _{\mathscr {W}_8^2}\nonumber \\&\lesssim \Vert g\Vert _{C^{\alpha }}(r-a)^{-1/2 + \alpha /4}\sum _{i=0}^3F^{(2)}_{32,i}(r-a,y,z_1,z_2)\nonumber \\&\qquad \times \Big (F^{(2)}_{32,i}(r-a,y,z_1,z_3) + F^{(2)}_{32,i}(r-a,y,z_2,z_4)\Big )\nonumber \\&\qquad + \Vert g\Vert _{C^\alpha }(r-a)^{-1/4 + \alpha /4}\sum _{i=0}^2F^{(4)}_{8,i}(r-a,y,z). \end{aligned}$$

By Lemma 5.6 and Lemma 6.4, we have that for l=1,2, $i \in \mathbb {Z}_{\ge 0}$ that

$$\begin{aligned}\Vert F^{(2)}_{32,i}(r-a,y,Z_l, Z_{l+2})\Vert _{L_2|\mathscr {F}_s} \lesssim \sup _{x \in \mathbb {T}}\Vert Z_l(x) - Z_{l+2}(x)\Vert _{L_{2,\infty }^{\mathscr {F}_s}} \lesssim [D^{u^l}]_{\mathscr {V}_p^\beta }(t-s)^{\beta }. \end{aligned}$$

Using this, and Lemma 5.6, we can see that

7.6 $$\begin{aligned}&\Vert \mathbb {E}^s\Big (F_{32,i}^{(2)}(r-a,y,Z_1,Z_2)F_{32,i}^{(2)}(r-a,y,Z_l, Z_{l+2})\Big )\Vert _{L_p} \nonumber \\&\lesssim \Vert \Vert F_{32,i}^{(2)}(r-a,y,Z_1,Z_2)\Vert _{L_2|\mathscr {F}_s}\Vert F_{32,i}^{(2)}(r-a,y,Z_l, Z_{l+2})\Vert _{L_2|\mathscr {F}_s}\Vert _{L_p} \nonumber \\&\lesssim [D^{u^l}]_{\mathscr {V}_p^\beta }(t-s)^{\beta } \Vert \Vert F^{(2)}_{32,i}(r-a,y,Z_1,Z_2)\Vert _{L_2|\mathscr {F}_s}\Vert _{L_p}\nonumber \\&\lesssim [D^{u^l}]_{\mathscr {V}_p^\beta }(t-s)^{\beta } \sup _{x \in \mathbb {T}}\Vert \phi ^{u^1(s,\cdot ),s}(a,x) - \phi ^{u^2(s,\cdot ),s}(a,x) \Vert _{L_{2,p}^{\mathscr {F}_s}}\ \nonumber \\&\lesssim \max _{l \in \{1,2\}}[D^{u^l}]_{\mathscr {V}_{2p}^\beta }(t-s)^{\beta } \sup _{x \in \mathbb {T}}\Vert u^1(s,x) - u^2(s,x)\Vert _{L_p}. \end{aligned}$$

Note moreover that by Jensen’s inequality for conditional expectations and Lemma 6.6,

7.7 $$\begin{aligned} \sum _{i=0}^2\Vert&\mathbb {E}^sF_{8,i}^{(4)}(r-a,y,Z)\Vert _{L_p}\le \sum _{i=0}^2\Vert F_{8,i}^{(4)}(r-a,y,Z)\Vert _{L_p}\nonumber \\&\lesssim (1+\max _{l \in \{1,2\}}[D^{u^l}]_{\mathscr {V}_{2p}^\beta }) \Big ([u^1,u^2]_{\mathscr {S}_p^{1/2}[S,T]} + \Vert u^1(S,\cdot ) - u^2(S,\cdot )\Vert _{\mathbb {B}(\mathbb {T}, L_p)}\Big )|t-s|^{1/2}. \end{aligned}$$

Using (7.5), (7.6) and (7.7), we get that

$$\begin{aligned}&\Vert \mathbb {E}^s{\Gamma }(Z)\Vert _{L_p} \\&\quad \lesssim \Vert g\Vert _{C^\alpha }(r-a)^{-1/2 + \alpha /4}\\&\qquad \qquad \sum _{i=0}^3 \sum _{l \in \{1,2\}}\Vert \mathbb {E}^s \Big (F_{32,i}^{(2)}(r-a,y,Z_1, Z_2) F_{32,i}^{(2)}(r-a,y,Z_l, Z_{l+2})\Big )\Vert _{L_p}\\&\qquad + \Vert g\Vert _{C^\alpha }(r-a)^{-1/4 +\alpha /4}\sum _{i=0}^2 \Vert \mathbb {E}^s F_{8,i}^{(4)}(r-a,y, Z)\Vert _{L_p}\\&\qquad \lesssim \Vert g\Vert _{C^\alpha }(r-a)^{-1/2 + \alpha /4} \max _{l \in \{1,2\}}[D^{u^l}]_{\mathscr {V}_{2p}^\beta }(t-s)^{\beta }\sup _{x \in \mathbb {T}}\Vert u^1(s,x) - u^2(s,x)\Vert _{L_p} \\&\qquad + \Vert g\Vert _{C^\alpha }(r-a)^{-1/4 + \alpha /4}\\&\qquad \times (1+\max _{l \in \{1,2\}}[D^{u^l}]_{\mathscr {V}_{2p}^\beta })([u^1, u^2]_{\mathscr {S}_p^{1/2}[S,T]} + \Vert u^1(S,\cdot ) - u^2(S,\cdot )\Vert _{\mathbb {B}(\mathbb {T}, L_p)})(t-s)^{1/2}\\&\quad \lesssim \Vert g\Vert _{C^\alpha }(1+\max _{l \in \{1,2\}}[D^{u^l}]_{\mathscr {V}_{2p}^\beta })([u^1, u^2]_{\mathscr {S}_p^{1/2}[S,T]} + \Vert u^1(S,\cdot ) - u^2(S,\cdot )\Vert _{\mathbb {B}(\mathbb {T}, L_p)})\\&\qquad \times \Big ((r-a)^{-1/2 + \alpha /4}(t-s)^{\beta } + (r-a)^{-1/4 + \alpha /4}(t-s)^{1/2} \Big ). \end{aligned}$$

Therefore, going back to (7.3) and using (7.4) and the above bound, we get

7.8 $$\begin{aligned}&\Vert \mathbb {E}^s \delta A_{s,a,t}\Vert _{L_p}\nonumber \\&\quad \lesssim \int _a^t \int _{\mathbb {T}} p_{T-r}(x,y)\Vert \mathbb {E}^s \Gamma _{r,y}(Z)\Vert _{L_p} dydr\nonumber \\&\quad \lesssim \Vert g\Vert _{C^\alpha }(1+\max _{l \in \{1,2\}}[D^{u^l}]_{\mathscr {V}_{2p}^\beta })([u^1, u^2]_{\mathscr {S}_p^{1/2}[S,T]} + \Vert u^1(S,\cdot ) - u^2(S,\cdot )\Vert _{\mathbb {B}(\mathbb {T}, L_p)})\nonumber \\&\qquad \times \Big ((t-s)^{\beta }\int _a^t \int _{\mathbb {T}}p_{T-r}(x,y)(r-a)^{-1/2 + \alpha /4}dydr\nonumber \\&\qquad + (t-s)^{1/2}\int _a^t \int _{\mathbb {T}}p_{T-r}(x,y)(r-a)^{-1/4 + \alpha /4}dydr\Big )\nonumber \\&\lesssim \Vert g\Vert _{C^\alpha }(1+\max _{l \in \{1,2\}}[D^{u^l}]_{\mathscr {V}_{2p}})([u^1, u^2]_{\mathscr {S}_p^{1/2}[S,T]} + \Vert u^1(S,\cdot ) - u^2(S,\cdot )\Vert _{\mathbb {B}(\mathbb {T}, L_p)})\nonumber \\&\qquad \times \Big ((t-s)^{\beta }(t-a)^{1/2 + \alpha /4} + (t-s)^{1/2}(t-a)^{3/4 + \alpha /4}\Big )\nonumber \\&\quad \lesssim \Vert g\Vert _{C^\alpha }(1+\max _{l \in \{1,2\}}[D^{u^l}]_{\mathscr {V}_{2p}})([u^1, u^2]_{\mathscr {S}_p^{1/2}[S,T]} + \Vert u^1(S,\cdot ) - u^2(S,\cdot )\Vert _{\mathbb {B}(\mathbb {T}, L_p)})\nonumber \\&\qquad \times \Big ((t-s)^{\beta + 1/2 + \alpha /4} + (t-s)^{5/4 + \alpha /4}\Big ). \end{aligned}$$

Note that $\beta + 1/2 + \alpha /4$ and 5/4+α/4 are greater than 1 by the assumptions that $\beta > 1/2 - \alpha /4$ and that α>-1. Consequently, by (7.2) and (7.8), we have that the condition (4.1) is satisfied. In addition, by using Lemma 6.4 and the regularity of *g*, it is straightforward to see that the process

$$\begin{aligned} \mathscr {A}_t:=\int _0^t \int _{\mathbb {T}}p_{T-r}(x,y)\big (g(u^1(r,y)) - g(u^2(r,y))\big ) dydr \end{aligned}$$

satisfies (4.2) and (4.3). Consequently, the conclusion follows from Lemma 4.1 and the fact that $(S,T) \in [0,1]_{\le }^2$ was arbitrary. □

### Corollary 7.5

Let $p \in [2,\infty )$ and let $\sigma \in C^4$ such that there exists a constant μ>0 such that $\sigma ^2(x) \ge \mu ^2$. For i=1,2, let $b^i \in C^{\alpha }$ and let $u^i$ be regularised solutions of

$$(\partial _t - \Delta )u^i = b^i(u^i) + \sigma (u^i)\xi (dy,dr)$$

in the class $\mathscr {U}^\beta$ for some $\beta \in \big (\frac{1}{2} - \frac{\alpha }{4}, 1+\frac{\alpha }{4}\big ]$. There exists a constant $N = N(p, \Vert \sigma \Vert _{C^4}, \mu , \alpha , \beta )$ such that for all $g^1, g^2 \in C^\infty$, $(s,t) \in [0,1]^2_{\le }$, we have

$$\begin{aligned}&\Big \Vert \int _s^t \int _{\mathbb {T}} p_{t-r}(x,y)\big (g^1(u^1(r,y)) - g^2(u^2(r,y))\big )dydr\Big \Vert _{L_p}\\&\quad \le N\big (1+\max _{i \in \{1,2\}}[D^{u^i}]_{\mathscr {V}_{2p}^\beta }\big )|t-s|^{(3+\alpha )/4}\\&\qquad \times \Big (\Vert g^1 - g^2\Vert _{C^{\alpha -1}} + \Vert g^2\Vert _{C^\alpha }\Big ([u^1,u^2]_{\mathscr {S}_p^{1/2}[s,t]} + \Vert u^1(s,\cdot ) - u^2(s,\cdot )\Vert _{\mathbb {B}(\mathbb {T}, L_p)}\Big )\Big ). \end{aligned}$$

### Proof

Since $\beta >\frac{1}{2} - \frac{\alpha }{4} = \frac{1}{4} - \frac{\alpha -1}{4}$, we can see that the condition of Corollary 7.2 is satisfied with $\lambda = \alpha -1$. The desired result follows from Corollary 7.2 and Lemma 7.4 by the triangle inequality. □

## The $\mathscr {S}_p$-bracket of two solutions

Throughout the section we work with the following assumption:

### Assumption 8.1

Let $\sigma \in C^4$ such that there exists constant μ>0 such that $\sigma ^2(x) \ge \mu ^2$. Let $\alpha \in (-1,0)$, $\beta \in (\frac{1}{2} - \frac{\alpha }{4}, 1+\frac{\alpha }{4}]$, and suppose that for i=1,2 we are given $b^i \in C^{\alpha +}$ and that $u^i$ are regularised solutions of

$$(\partial _t - \Delta )u^i = b^i(u^i) + \sigma (u^i)\xi (dy,dr)$$

in the class $\mathscr {U}^\beta$ with initial conditions $u^i(0,\cdot ) = u_0^i \in C(\mathbb {T})$.

Recall the definition of the $\mathscr {S}_p^{1/2}$-bracket from (6.4). Informally, the aim of this section is to show that

$${[}u^1,u^2]_{\mathscr {S}_p^{1/2}[0,1]} \lesssim \Vert u^1_0 - u^2_0\Vert _{\mathbb {B}(\mathbb {T})}+ \Vert b^1 - b^2\Vert _{C^{\alpha -1}}.$$

### Lemma 8.2

Let Assumption 8.1 hold and let $p \in [2,\infty )$. Then $[u^1,u^2]_{\mathscr {S}_p^{1/2}} <\infty$. Moreover there exists a constant $N = N(p, \mu ,\Vert \sigma \Vert _{C^4}, \alpha , \beta )$ such that

8.1 $$\begin{aligned} \,&[u^1,u^2]_{\mathscr {S}_p^{1/2}[s,t]}\nonumber \\&\quad \le N \big (1+\max _{i \in \{1,2\}}[D^{u^i}]_{\mathscr {V}_{2p}^\beta }\big )(1+\Vert b^2\Vert _{C^\alpha })\nonumber \\&\qquad \times \Big (\Vert b^1 - b^2 \Vert _{C^{\alpha -1}}+[u^1,u^2]_{\mathscr {S}^{1/2}_p[s,t]} + \Vert u^1(s,\cdot )- u^2(s,\cdot )\Vert _{\mathbb {B}(\mathbb {T}, L_p)} \Big )(t-s)^{ (1+\alpha )/4} . \end{aligned}$$

### Proof

Let $(S,T) \in [0,1]_{\le }^2$. We begin by verifying that the $\mathscr {S}_p^{1/2}$-bracket is finite. By the triangle inequality, and by Lemma 6.4, we have for $(s,t) \in [S,T]_{\le }^2$ that

$$\begin{aligned}&\sup _{x \in \mathbb {T}}\Vert u^1(t,x) - \phi ^{u^1(s,\cdot ),s}(t,x) - u^2(t,x) + \phi ^{u^2(s,\cdot ),s}(t,x)\Vert _{L_{p,\infty }^{\mathscr {F}_s}}\\&\quad \le \max _{i \in \{1,2\}}\sup _{x \in \mathbb {T}}\Vert u^i(t,x) - \phi ^{u^i(s,\cdot ),s}(t,x)\Vert _{L_{p,\infty }^{\mathscr {F}_s}}\\&\quad \lesssim \max _{i \in \{1,2\}}[D^{u^i}]_{\mathscr {V}_p^\beta }(t-s)^{\beta }\lesssim \max _{i \{1,2\}}[D^{u^i}]_{\mathscr {V}_p^\beta }(t-s)^{1/2}, \end{aligned}$$

where we used that by assumption we have $\beta \ge \frac{1}{2}-\frac{\alpha }{4} > \frac{1}{2}$. Thus by the fact that $\Vert \cdot \Vert _{L_p} = \Vert \Vert \cdot \Vert _{L_p|\mathscr {F}_s}\Vert _{L_p} \le \Vert \cdot \Vert _{L_{p,\infty }^{\mathscr {F}_s}}$, it follows that

8.2 $$\begin{aligned} \,[u^1,u^2]_{\mathscr {S}_p^{1/2}} \lesssim \max _{i \in \{1,2\}}[D^{u^i}]_{\mathscr {V}_p^\beta }, \end{aligned}$$

which is finite, since by assumption $u^i \in \mathscr {U}^\beta$. Note that for $(s,t) \in [0,1]_{\le }^2$, $x \in \mathbb {T}$ we have by (5.30) and by (3.2) that

$$\begin{aligned}&u^1(t,x) - u^2(t,x) - \phi ^{u^1(s,\cdot ),s}(t,x) +\phi ^{u^2(s,\cdot ),s}(t,x) \\&\quad = u^1(t,x) - u^2(t,x) - P_{t-s}\big (u^1(s,\cdot ) - u^2(s,\cdot )\big )(x)\\  &\qquad \qquad - \int _s^t \int _{\mathbb {T}}p_{t-r}(x,y)\big (\sigma (\phi ^{u^1(s,\cdot ),s}(r,y)) - \sigma (\phi ^{u^2(s,\cdot ),s}(r,y)\big )\xi (dy,dr)\\&\quad = \Big (D^{u^1}_t -D^{u^2}_t - P_{t-s}D^{u^1}_s + P_{t-s}D^{u^2}_s\Big )(x) \\&\qquad + \int _s^t \int _{\mathbb {T}}p_{t-r}(x,y)\big (\sigma (u^1(r,y)) - \sigma (u^2(r,y))\\&\qquad - \sigma (\phi ^{u^1(s,\cdot ),s}(r,y))+ \sigma (\phi ^{u^2(s,\cdot ),s}(r,y))\big ) \xi (dy,dr)\\&\quad =: I(s, t,x)+J(s, t,x). \end{aligned}$$

For i=1,2 let $(b^{i,n})_{n \in \mathbb {N}} \subset C^\infty$ with $b^{i,n} \rightarrow b^i$ in $C^\alpha$. Then by Definition 3.2 and by Fatou’s lemma, we have

8.3 $$\begin{aligned} \Vert I(s, t,x)\Vert _{L_p} \lesssim \liminf _{n \rightarrow \infty } \Big \Vert \int _s^t \int _{\mathbb {T}}p_{t-r}(x,y) \big (b^{1,n}(u^1(r,y)) - b^{2,n}(u^2(r,y))\big )dydr\Big \Vert _{L_p} \end{aligned}$$

So by Corollary 7.5 we have

$$\begin{aligned}&\Vert I(s, t,x)\Vert _{L_p}\lesssim |t-s|^{(3+\alpha )/4}(1+\max _{i \in \{1,2\}}[D^{u^i}]_{\mathscr {V}_{2p}^\beta })\\&\quad \times \Big (\Vert b^1 -b^2\Vert _{C^{\alpha -1}} + \Vert b^2\Vert _{C^\alpha }\Big ([u^1,u^2]_{\mathscr {S}_p^{1/2}[s,t]} + \Vert u^1(s,\cdot ) - u^2(s,\cdot )\Vert _{\mathbb {B}(\mathbb {T}, L_p)}\Big )\Big ). \end{aligned}$$

Note moreover that by Lemma 6.8 we have

$$\begin{aligned}&\Vert J(s, t,x)\Vert _{L_p} \\&\quad \lesssim [D^{u^1}]_{\mathscr {V}_{2p}^\beta }\Vert u^1(s,\cdot ) -u^2(s,\cdot )\Vert _{\mathbb {B}(\mathbb {T}, L_p)}(t-s)^{ \frac{1}{4}+\beta }\\&\qquad + \Big (\int _s^t\int _{\mathbb {T}} p^2_{t-r}(x,y)\big \Vert u^1(r,y) - u^2(r,y) - \phi ^{u^1(s,\cdot ),s}(r,y) + \phi ^{u^2(s,\cdot ),s}(r,y)\big \Vert _{L_p}^2 dydr \Big )^{1/2}. \end{aligned}$$

By our bounds on *I*, *J* and by the observation that $\frac{1}{4} + \beta> \frac{1}{4} + \frac{1}{2} - \frac{\alpha }{4} > \frac{3}{4} + \frac{\alpha }{4}$, we conclude that

$$\begin{aligned}&\Vert u^1(t,x) - u^2(t,x) - \phi ^{u^1(s,\cdot ),s}(t,x) +\phi ^{u^2(s,\cdot ),s}(t,x)\Vert _{L_p}^2 \lesssim \\&\quad \lesssim (1+\max _{i \in \{1,2\}}[D^{u^i}]_{\mathscr {V}_{2p}^\beta })^2(1+\Vert b^2\Vert _{C^\alpha })^2\\&\qquad \times \Big (\Vert b^1 - b^2 \Vert _{C^{\alpha -1}}+[u^1,u^2]_{\mathscr {S}_p^{1/2}[s,t]} + \Vert u^1(s,\cdot )- u^2(s,\cdot )\Vert _{\mathbb {B}(\mathbb {T}, L_p)} \Big )^2(t-s)^{(3+\alpha )/2} \\&\qquad + \int _s^t\int _{\mathbb {T}} p^2_{t-r}(x,y)\big \Vert u^1(r,y) - u^2(r,y) - \phi ^{u^1(s,\cdot ),s}(r,y) + \phi ^{u^2(s,\cdot ),s}(r,y)\big \Vert _{L_p}^2 dydr. \end{aligned}$$

Note that the norm in the integrand is bounded in (*r*, *y*), since it is bounded by $[u^1,u^2]_{\mathscr {S}_p^{1/2}}$, which is finite by (8.2). Using Lemma 11.4, and Lemma 6.3 (where we recall that $S \le s \le t$), we get

$$\begin{aligned}&\Vert u^1(t,x) - u^2(t,x) - \phi ^{u^1(s,\cdot ),s}(t,x) +\phi ^{u^2(s,\cdot ),s}(t,x)\Vert _{L_p}\\&\quad \lesssim (1+\max _{i \in \{1,2\}}[D^{u^i}]_{\mathscr {V}_{2p}^\beta })(1+\Vert b^2\Vert _{C^\alpha })\\&\qquad \times \Big ([u^1,u^2]_{\mathscr {S}_p^{1/2}[S,t]} + \Vert u^1(S,\cdot )- u^2(S,\cdot )\Vert _{\mathbb {B}(\mathbb {T}, L_p)} +\Vert b^1 - b^2 \Vert _{C^{\alpha -1}} \Big )(t-s)^{ (3+\alpha )/4}. \end{aligned}$$

Therefore dividing both sides by $(t-s)^{1/2}$ and taking supremum over $(s,t) \in [S,T]^2_<$, we obtain the desired bound with (*S*, *T*) in place of (*s*, *t*). Now the desired result follows by the fact that $(S,T) \in [0,1]_{\le }^2$ was arbitrary. □

### Lemma 8.3

(Splitting the $\mathscr {S}_p^{1/2}$-bracket) Let Assumption 8.1 hold and let $p \in [2,\infty )$. There exists a constant $N = N(p,\Vert \sigma \Vert _{C^{4}}, \mu , \alpha , \beta )$ such for all $(S,T) \in [0,1]_{\le }^2$ and Q∈[S,T] we have

$$\begin{aligned}&{}[u^1,u^2]_{\mathscr {S}_p^{1/2}[S,T]} \\&\quad \le N(1+ \max _{i \in \{1,2\}}[D^{u^i}]_{\mathscr {V}_{2p}^\beta })\Big ([u^1,u^2]_{\mathscr {S}_p^{1/2}[S,Q]} + \Vert u^1(S,\cdot ) - u^2(S,\cdot )\Vert _{\mathbb {B}(\mathbb {T}, L_p)}\Big )\\&\qquad +2[u^1,u^2]_{\mathscr {S}_p^{1/2}[Q,T]}. \end{aligned}$$

### Proof

For $(s,t) \in [0,1]_{\le }^2$, we set

$$ A(s,t) := \sup _{x \in \mathbb {T}}\Vert u^1(t,x) - u^2(t,x) - \phi ^{u^1(s,\cdot ),s}(t,x) +\phi ^{u^1(s,\cdot ),s}(t,x) \Vert _{L_p}. $$

For $(s,t) \in [S,Q]^2_{\le }$ or $(s,t) \in [Q,T]^2_{\le }$, we clearly have

8.4 $$\begin{aligned} A(s,t) \le \big ([u^1,u^2]_{\mathscr {S}_p^{1/2}[S,Q]}+[u^1,u^2]_{\mathscr {S}_p^{1/2}[Q,T]}\big )|t-s|^{1/2}. \end{aligned}$$

For s≤Q<t, by using the triangle inequality, conditioning with respect to $\mathcal {F}_Q$, and keeping in mind the definition of $F^{(4)}_{p,0}$ (see (6.10)) and Lemma 11.11, we have

$$\begin{aligned} A(s,t)&\le A(Q, t)+ \sup _{x \in \mathbb {T}}\Vert \phi ^{u^1(Q,\cdot ), Q}(t,x) - \phi ^{u^2(Q,\cdot ), Q}(t,x) - \phi ^{u^1(s,\cdot ),s}(t,x) + \phi ^{u^2(s,\cdot ),s}(t,x)\Vert _{L_p} \\&= A(Q, t)+ \sup _{x \in \mathbb {T}}\Vert \phi ^{u^1(Q,\cdot ), Q}(t,x) - \phi ^{u^2(Q,\cdot ), Q}(t,x) - \phi ^{\phi ^{u^1(s,\cdot ),s}(Q,\cdot ), Q}(t,x) + \phi ^{\phi ^{u^2(s,\cdot ),s}(Q,\cdot ), Q}(t,x)\Vert _{L_p} \\&= A(Q, t)+\sup _{x \in \mathbb {T}}\big \Vert F^{(4)}_{p,0}\big (t-Q,\, x,\, \phi ^{u^1(s,\cdot ),s}(Q,\cdot ),\phi ^{u^2(s,\cdot ),s}(Q,\cdot ), u^1(Q,\cdot ), u^2(Q,\cdot )\big )\big \Vert _{L_p}. \end{aligned}$$

From this and Lemma 6.6, we conclude that for s≤Q<t

8.5 $$\begin{aligned} A(s,t)&\le [u^1,u^2]_{\mathscr {S}_p^{1/2}[Q,T]}|t-s|^{1/2} \nonumber \\&\qquad + N(1+\max _{i \in \{1,2\}}[D^{u^i}]_{\mathscr {V}_{2p}^\beta })\Big ([u^1,u^2]_{\mathscr {S}_p^{1/2}[S,Q]} \nonumber \\&\qquad + \Vert u^1(S,\cdot ) - u^2(S,\cdot )\Vert _{\mathbb {B}(\mathbb {T}, L_p)}\Big ) |t-s|^{1/2}.\qquad \end{aligned}$$

By the above combined with (8.4), the inequality (8.5) holds for any $(s,t) \in [S, T]^2_{\le }$, from which the claim follows. □

### Lemma 8.4

Let Assumption 8.1 hold and let $K \in \mathbb {Z}_{\ge 2}$, $p \in [2,\infty )$. There exists a constant $N = N(p,\Vert \sigma \Vert _{C^{4}}, K, \mu , \alpha , \beta )$ such that with $M : = N(1+ \max _{i \in \{1,2\}} [D^{u^i}]_{\mathscr {V}_{2p}})$ we have

$${[}u^1,u^2]_{\mathscr {S}_p^{1/2}} \le (K-1)M^{K-1}\Vert u^1_0 - u^2_0\Vert _{\mathbb {B}(\mathbb {T})} + 2\sum _{i=0}^{K-1}M^{i}[u^1,u^2]_{\mathscr {S}^{1/2}_p[\frac{K-i-1}{K}, \frac{K-i}{K}]}.$$

### Proof

Let $a_{s,t} := [u^1,u^2]_{\mathscr {S}_p^{1/2}[s,t]}$ and $u_0^{1,2} := \Vert u^1_0 - u^2_0\Vert _{\mathbb {B}(\mathbb {T})}$. We will begin by using induction to show that for all $n \in \{1,\dots , K-1\}$ we have

8.6 $$\begin{aligned} a_{0,1} \le M^n a_{0,\frac{K-n}{K}} + \Big (\sum _{i=1}^n M^i\Big )u^{1,2}_0+2\sum _{i=0}^{n-1}M^i a_{\frac{K-i -1}{K}, \frac{K-i}{K}}. \end{aligned}$$

By Lemma 8.3 we have that

$$a_{0,1} \le M(a_{0,\frac{K-1}{K}} + u_0^{1,2}) + 2a_{\frac{K-1}{K}, 1},$$

therefore (8.6) holds for the initial case n=1. Now suppose that (8.6) holds for some $n \in \{1, \dots , K-2\}$. We will show that it also holds for n+1. To this end, we first apply the induction hypothesis and then Lemma 8.3, to get that

$$\begin{aligned} a_{0,1}&\le M^n a_{0,\frac{K-n}{K}} + \Big (\sum _{i=1}^n M^i\Big )u_0^{1,2} + 2\sum _{i=0}^{n-1}M^i a_{\frac{K-i -1}{K}, \frac{K-i}{K}}\\&\le M^n\Big ( M \big (a_{0,\frac{K-n-1}{K}}+ u_0^{1,2}\big )+ 2a_{\frac{K-n-1}{K},\frac{K-n}{K}} \Big ) \\&\qquad +\Big (\sum _{i=1}^n M^i\Big )u_0^{1,2} + 2\sum _{i=0}^{n-1}M^i a_{\frac{K-i -1}{K}, \frac{K-i}{K}}\\&= M^{n+1}a_{0,\frac{K-n-1}{K}} + M^{n+1}u_{0}^{1,2} + \Big (\sum _{i=1}^n M^i\Big )u_0^{1,2} + 2M^n a_{\frac{K-n-1}{K}, \frac{K-n}{K}} \\&\qquad + 2\sum _{i=0}^{n-1}M^i a_{\frac{K-i -1}{K}, \frac{K-i}{K}}\\&= M^{n+1}a_{0,\frac{K-n-1}{K}} + \Big (\sum _{i=1}^{n+1} M^i\Big )u_0^{1,2} + 2\sum _{i=0}^{n}M^i a_{\frac{K-i -1}{K}, \frac{K-i}{K}} \end{aligned}$$

as required. Therefore (8.6) is proven. Now choosing n:=K-1 in (8.6) and assuming without loss of generality that N≥1 and therefore M≥1, we get

$$\begin{aligned} a_{0,1}&\le \Big (\sum _{i=1}^{K-1} M^i\Big )u_0^{1,2} + M^{K-1}a_{0,\frac{1}{K}} + 2 \sum _{i=0}^{K-2}M^i a_{\frac{K-i -1}{K}, \frac{K-i}{K}}\\&\le (K-1)M^{K-1}u_{0}^{1,2} + 2\sum _{i=0}^{K-1}M^i a_{\frac{K-i -1}{K}, \frac{K-i}{K}} \end{aligned}$$

as required. □

### Lemma 8.5

Let Assumption 8.1 hold. For all $p \in [2,\infty )$ there exists a positive constant $K_0 = K_0(\max _{i \in \{1,2\}}[D^{u^i}]_{\mathscr {V}_{2p}^\beta }, p, \Vert \sigma \Vert _{C^4}, \mu , \alpha , \beta )$ such that if $K \in \mathbb {Z}$ satisfies $K> K_0$, then there exists a constant $M = {M}(p, \Vert \sigma \Vert _{C^1}, \alpha , \beta )$ such that for all $n \in \{0,\dots , K\}$ we have that

$${[}u^1,u^2]_{\mathscr {S}_p^{1/2}[\frac{n}{K},\frac{n+1}{K}]} \le M^K\left( \Vert u^1(0,\cdot ) - u^2(0,\cdot )\Vert _{\mathbb {B}(\mathbb {T})} + \Vert b^1 - b^2\Vert _{C^{\alpha -1}}\right) .$$

### Proof

By Lemma 8.2 there exists some ${N} = {N}(p,\mu , \Vert \sigma \Vert _{C^4}, \alpha , \beta )>0$ such that for all $K \in \mathbb {N}$ and $n \in \{0,\dots ,K-1\}$ we have

$$\begin{aligned}&[u^1,u^2]_{\mathscr {S}_p^{1/2}[\frac{n}{K},\frac{n+1}{K}]}\\&\quad \le {N} (1+\max _{i \in \{1,2\}}[D^{u^i}]_{\mathscr {V}_{2p}^\beta })(1+\Vert b^2\Vert _{C^\alpha }) \\&\qquad \times \Big ([u^1,u^2]_{\mathscr {S}_p^{1/2}[\frac{n}{K},\frac{n+1}{K}]} + \big \Vert u^1\big (\frac{n}{K},\cdot \big )- u^2\big (\frac{n}{K},\cdot \big )\big \Vert _{\mathbb {B}(\mathbb {T}, L_p)}\\  &\qquad +\big \Vert b^1 - b^2 \big \Vert _{C^{\alpha -1}} \Big )K^{- (1+\alpha )/4}. \end{aligned}$$

Let $\lceil \cdot \rceil$ denote the ceiling function, and define the constants

8.7 $$\begin{aligned} \tilde{N}&:= {N}(1+\max _{i \in \{1,2\}}[D^{u^i}]_{\mathscr {V}_{2p}^\beta })(1+\Vert b^2\Vert _{C^\alpha }), \nonumber \\ K_0&:= \Big \lceil (2\tilde{N})^{\frac{4}{1+\alpha }} \Big \rceil . \end{aligned}$$

Then for $K>K_0$ we have that

8.8 $$\begin{aligned} [u^1,u^2]_{\mathscr {S}_p^{1/2}[\frac{n}{K},\frac{n+1}{K}]}&\le \big \Vert u^1\big (\frac{n}{K},\cdot \big )- u^2\big (\frac{n}{K},\cdot \big )\big \Vert _{\mathbb {B}(\mathbb {T}, L_p)} +\Vert b^1 - b^2 \Vert _{C^{\alpha -1}}. \end{aligned}$$

In particular, by choosing n=0, we have

8.9 $$\begin{aligned} \,[u^1,u^2]_{\mathscr {S}_p^{1/2}[0,\frac{1}{K}]} \le \Vert u^1_0 - u^2_0\Vert _{\mathbb {B}(\mathbb {T})} +\Vert b^1 - b^2\Vert _{C^{\alpha -1}}. \end{aligned}$$

Let

$$a_n := [u^1,u^2]_{\mathscr {S}_p^{1/2}[\frac{n}{K},\frac{n+1}{K}]} + \big \Vert u^1\big (\frac{n}{K},\cdot \big ) - u^2\big (\frac{n}{K},\cdot \big )\big \Vert _{\mathbb {B}(\mathbb {T}, L_p)} + \Vert b^1 - b^2\Vert _{C^{\alpha -1}}.$$

In the n=0 case we can use (8.9) to bound the first term to get

8.10 $$\begin{aligned} a_0 \le 2\left( \Vert u^1_0 - u^2_0\Vert _{\mathbb {B}(\mathbb {T})}+\Vert b^1 - b^2\Vert _{C^{\alpha -1}}\right) . \end{aligned}$$

For the general case $n \in \{1,\dots ,K-1\}$ we first use (8.8) to get rid of the first term in the definition of $a_n$, and then we apply Lemma 6.3 as follows:

$$\begin{aligned} a_n&\le 2\Big (\big \Vert u^1\big (\frac{n}{K},\cdot \big ) - u^2\big (\frac{n}{K},\cdot \big )\big \Vert _{\mathbb {B}(\mathbb {T}, L_p)} + \Vert b^1 - b^2\Vert _{C^{\alpha -1}}\Big )\\&\le {M}\Big ([u^1,u^2]_{\mathscr {S}_p^{1/2}[\frac{n-1}{K},\frac{n}{K}]} + \big \Vert u^1\big (\frac{n-1}{K},\cdot \big ) -u^2\big (\frac{n-1}{K},\cdot \big ) \big \Vert _{\mathbb {B}(\mathbb {T}, L_p)} + \Vert b^1 - b^2\Vert _{C^{\alpha -1}}\Big )\\&= {M}a_{n-1}\end{aligned}$$

for some constant ${M} ={M}(p, \Vert \sigma \Vert _{C^1}, \alpha , \beta )> 2$. Iterating this result *n* times and then applying (8.10), we get

$$\begin{aligned}a_n \le {M}^n a_0&\le M^n 2 \big (\Vert u^1_0 - u^2_0\Vert _{\mathbb {B}(\mathbb {T})}+\Vert b^1 - b^2\Vert _{C^{\alpha -1}}\big )\\&\le M^{K-1}M\big (\Vert u^1_0 - u^2_0\Vert _{\mathbb {B}(\mathbb {T})}+\Vert b^1 - b^2\Vert _{C^{\alpha -1}}\big ), \end{aligned}$$

which finishes the proof. □

### Lemma 8.6

Let Assumption 8.1 hold and let $p \in [2,\infty )$. There exists a continuous map (with dependencies as indicated below)

$$f = f_{p,\Vert \sigma \Vert _{C^4}, \mu , \alpha , \beta } :[0,\infty )^2 \rightarrow [0,\infty )$$

such that *f*(*x*, *y*) is increasing in both the *x* and *y* variables, and that the following inequality holds:

$${[}u^1,u^2]_{\mathscr {S}_p^{1/2}} \le f\big (\max _{i\ \in \{1,2\}}\Vert b^i\Vert _{C^\alpha }, \max _{i \in \{1,2\}}[D^{u^i}]_{\mathscr {V}_{2p}^\beta }\big )\left( \Vert u^1_0 - u^2_0\Vert _{\mathbb {B}(\mathbb {T})}+\Vert b^1 - b^2\Vert _{C^{\alpha -1}}\right) .$$

### Proof

Let $K \in \mathbb {Z}$ be sufficiently large so that it satisfies the assumption of Lemma 8.5. By (8.7) we know that we can choose $K = N_0(1+\max _{i \in \{1,2\}}[D^{u^i}]_{\mathscr {V}_{2p}^\beta })^{\frac{4}{1+\alpha }}(1+\Vert b^2\Vert _{C^\alpha })^{\frac{4}{1+\alpha }}$ with $N_0 = N_0(p,\mu , \Vert \sigma \Vert _{C^4}, \alpha , \beta )$. Then there exists a constant $M_1 = M_1(p,\Vert \sigma \Vert _{C^1}, \alpha , \beta ) \ge 1$ such that

8.11 $$\begin{aligned} \,[u^1,u^2]_{\mathscr {S}_p^{1/2}[\frac{n}{K},\frac{n+1}{K}]} \le M_1^K\big (\Vert u^1_0 - u^2_0\Vert _{\mathbb {B}(\mathbb {T})}+ \Vert b^1 - b^2\Vert _{C^{\alpha -1}}\big ). \end{aligned}$$

Recall moreover that by Lemma 8.4 there exists a constant $N_2 = N_2(p,\Vert \sigma \Vert _{C^{4}}, K, \mu , \alpha , \beta )$ such that for $M_2 : = N_2(1+ \max _{i \in \{1,2\}}[D^{u^i}]_{\mathscr {V}_{2p}^\beta }) \ge 1$ we have

8.12 $$\begin{aligned} \,[u^1,u^2]_{\mathscr {S}_p^{1/2}} \le (K-1)M_2^{K-1}\Vert u^1_0 - u^2_0\Vert _{\mathbb {B}(\mathbb {T})}+ 2\sum _{i=0}^{K-1} M_2^{i}[u^1,u^2]_{\mathscr {S}_p^{1/2}[\frac{K-i-1}{K}, \frac{K-i}{K}]}. \end{aligned}$$

By (8.11), we get that the second term on the right hand side of (8.12) is bounded by

$$\begin{aligned}&2\sum _{i=0}^{K-1}M_2^i M_1^{K}\big (\Vert u^1_0 - u^2_0\Vert _{\mathbb {B}(\mathbb {T})}+ \Vert b^1 - b^2\Vert _{C^{\alpha -1}}\big )\\&\qquad \le 2K(M_1M_2)^K\big (\Vert u^1_0- u^2_0\Vert _{\mathbb {B}(\mathbb {T})}+ \Vert b^1 - b^2\Vert _{C^{\alpha -1}}\big ). \end{aligned}$$

Therefore

$$\begin{aligned} \,[u^1,u^2]_{\mathscr {S}_p^{1/2}}\le K\big (M_2^{K-1} + 2(M_1M_2)^K\big )\big (\Vert u^1_0- u^2_0\Vert _{\mathbb {B}(\mathbb {T})}+ \Vert b^1 - b^2\Vert _{C^{\alpha -1}}\big ), \end{aligned}$$

and the desired result follows by the definitions of $K,M_1,M_2$. □

## The $\mathscr {V}_p$-bracket of the drift and an a priori estimate

The aim of this section is to provide a priori bounds on a regularised solution of (1.1) under Assumption 3.1.

### Lemma 9.1

Let Assumption 3.1 hold, let $\beta \in (\frac{1}{4} - \frac{\alpha }{4}, 1 + \frac{\alpha }{4}]$ and assume that *u* is a regularised solution of (1.1) in the class $\mathscr {U}_2^\beta$. Then *u* is also of class $\mathscr {U}^\beta$. Moreover for all $p \in [2,\infty )$ there exists a constant $N = N(p,\Vert \sigma \Vert _{C^4}, \mu , \alpha , \beta )>0$ such that

$${[}D^u]_{\mathscr {V}_p^\beta } \le N\exp \Big (N \Vert b\Vert _{C^\alpha }^{\frac{4}{\alpha +3}}\Big ).$$

### Proof

Let $(b^n)_{n \in \mathbb {N}} \subset C^\infty$ be a sequence of smooth functions such that $b^n \rightarrow b$ in $C^\alpha$. Then by the definition of $D^u$ (see (3.1)), by the conditional Fatou’s lemma and the usual Fatou’s lemma, for p≥2 and for $(s,t) \in [0,1]_{\le }^2$, $x \in \mathbb {T}$ we have that

$$\begin{aligned} \Vert D_t^u(x) - P_{t-s}D_s^u(x)\Vert _{L_{p,\infty }^{\mathscr {F}_s}} \lesssim \liminf _{n \rightarrow \infty } \sup _{x \in \mathbb {T}}\Big \Vert \int _s^t \int _{\mathbb {T}}p_{t-r}(x,y) b^n(u(r,y))dydr\Big \Vert _{L_{p,\infty }^{\mathscr {F}_s}}. \end{aligned}$$

Therefore by applying Corollary 7.2 we know that

$$\begin{aligned} \Vert D_t^u(x) - P_{t-s}D_s^u(x)\Vert _{L_{p,\infty }^{ \mathscr {F}_s}}&\lesssim \Vert b\Vert _{C^\alpha }\Big ((t-s)^{1 + \alpha /4} + [D^u]_{\mathscr {V}_2^\beta [s,t]}(t-s)^{\beta + \frac{\alpha +3}{4}}\Big )\\&\lesssim \Vert b\Vert _{C^\alpha }\Big ((t-s)^{\beta } + [D^u]_{\mathscr {V}_2^\beta [s,t]}(t-s)^{\beta + \frac{\alpha +3}{4}}\Big ), \end{aligned}$$

where we used the assumption that $\beta \le 1+\alpha /4$. Hence there exists $\tilde{N} = \tilde{N}(p,\Vert \sigma \Vert _{C^4}, \mu , \alpha , \beta )$ such that for all $(s,t) \in [0,1]_{\le }^2$ we have

9.1 $$\begin{aligned} [D^u]_{\mathscr {V}_p^\beta [s,t]} \le \tilde{N}\Vert b\Vert _{C^\alpha } + \tilde{N}\Vert b\Vert _{C^\alpha }[D^u]_{\mathscr {V}_2^\beta [s,t]}(t-s)^{(\alpha +3)/4}. \end{aligned}$$

Since we assumed that $u \in \mathscr {U}_2^\beta$, we have $[D^u]_{\mathscr {V}_2^\beta } <\infty$, and thus by the inequality (9.1) we have $[D^u]_{\mathscr {V}_p^\beta }<\infty$, and thus $u \in \mathscr {U}_p^\beta$. Since p≥2 was arbitrary, it follows that $u \in \mathscr {U}^\beta .$

Note that on the right hand side of (9.1), the $[D^u]_{\mathscr {V}_2^\beta [s,t]}$ may be replaced with $[D^u]_{\mathscr {V}_p^\beta [s,t]}$. Hence choosing sufficiently large $K \in \mathbb {N}$, it follows that

9.2 $$\begin{aligned} \max _{i \in \{0,\dots ,K-1\}}[D^u]_{\mathscr {V}_p^\beta [\frac{i}{K},\frac{i+1}{K}]} \lesssim \Vert b\Vert _{C^\alpha }. \end{aligned}$$

To this end we may pick $K := \Big \lceil (2\tilde{N}\Vert b\Vert _{C^\alpha })^{\frac{4}{\alpha +3}} \Big \rceil$. Moreover using Lemma 11.9 and the inequality (9.2), we obtain that

$$\begin{aligned} \,[D^u]_{\mathscr {V}_p^\beta [0,1]}&\le 2^K\sum _{i=0}^{K-1} [D^u]_{\mathscr {V}_p^\beta [\frac{i}{K},\frac{i+1}{K}]}\\&\lesssim 2^K\sum _{i=0}^{K-1} \Vert b\Vert _{C^\alpha } \lesssim K2^K\Vert b\Vert _{C^\alpha }, \end{aligned}$$

which finishes the proof . □

### Lemma 9.2

(The regularity of $D^u$) Let Assumption 3.1 hold, and let $p \in [2,\infty )$, $\beta \in (\frac{1}{2} - \frac{\alpha }{4}, 1+\frac{\alpha }{4}]$. There exists a constant $N = N(p,\Vert \sigma \Vert _{C^4}, \mu , \alpha , \beta )>0$ such that if *u* is a regularised solution of class $\mathscr {U}^\beta$, then

$$\Vert D^u\Vert _{C^{\frac{1}{4},\frac{1}{2}}([0,1]\times \mathbb {T}, L_p)} \le N(1+\Vert b\Vert _{C^\alpha })(1+[D^u]_{\mathscr {V}_p^\beta }).$$

### Proof

Noting that $\Vert \cdot \Vert _{L_p} = \Vert \Vert \cdot \Vert _{L_p|\mathscr {F}_s}\Vert _{L_p} \le \Vert \Vert \cdot \Vert _{L_p|\mathscr {F}_s}\Vert _{L_\infty }$ and that from the definition of $D^u$ (see (3.1)) we have $D_0^u =0$, we conclude for all $(t,x) \in [0,1]\times \mathbb {T}$ that

9.3 $$\begin{aligned} \Vert D_t^u(x)\Vert _{L_p}&\le \Vert D_t^u(x) - P_{t-0}D_0^u(x)\Vert _{L_{p,\infty }^{\mathscr {F}_s}}\le [D^u]_{\mathscr {V}_p^\beta }. \end{aligned}$$

Let $(b^n)_{n \in \mathbb {N}} \subset C^\infty$ be a sequence of smooth functions such that $b^n \rightarrow b$ in $C^\alpha$. By (3.1), Fatou’s lemma and Corollary 7.3, we can see that for all $x, \bar{x} \in \mathbb {T}$ and t∈[0,1] we have

9.4 $$\begin{aligned} \Vert D_t^u(x) - D_t^u(\bar{x})\Vert _{L_p}&\le \liminf _{n \rightarrow \infty }\Big \Vert \int _0^t \int _{\mathbb {T}} (p_{t-r}(x,y) - p_{t-r}(\bar{x}, y))b^n(u(r,y))dydr\Big \Vert _{L_p}\nonumber \\&\lesssim \Vert b\Vert _{C^\alpha }(1+[D^u]_{\mathscr {V}_p^\beta })|x-\bar{x}|^{1/2}. \end{aligned}$$

By (9.3) and (9.4) we can see that

9.5 $$\begin{aligned} \sup _{t \in [0,1]}\Vert D^u_t\Vert _{C^{1/2}(\mathbb {T}) }\lesssim (1+\Vert b\Vert _{C^\alpha })(1+[D^u]_{\mathscr {V}_p^\beta }). \end{aligned}$$

Finally, note that since by assumption we have $\beta>\frac{1}{2}-\frac{\alpha }{4} > \frac{1}{4}$, and thus

9.6 $$\begin{aligned} \,[D^u]_{\mathscr {V}_p^{1/4}} \le [D^u]_{\mathscr {V}_p^{\beta }}. \end{aligned}$$

By (9.5) and (9.6), the desired bound holds for the $C^{0,\frac{1}{2}}([0,1]\times \mathbb {T}, L_p)$-norm and for the $\mathscr {V}_p^{1/4}$-bracket. Hence by Lemma 11.10 the proof is finished. □

### Lemma 9.3

(An a priori estimate) Let Assumption 3.1 hold, and let $p\in [2,\infty )$, $\varepsilon \in (0, \frac{1}{2})$, $\beta \in (\frac{1}{2} - \frac{\alpha }{4}, 1+\frac{\alpha }{4}]$. There exists a constant $N = N(p,\Vert \sigma \Vert _{C^4}, \mu , \alpha , \beta , \varepsilon )>0$ such that if *u* is a regularised solution of class $\mathscr {U}^\beta$, then

$$\begin{aligned}\,\Vert u -P_\cdot u_0(\cdot )\Vert _{C^{1/4 - \varepsilon /2, 1/2-\varepsilon }([0,1]\times \mathbb {T}, L_p)} \le N(1+\Vert b\Vert _{C^\alpha })(1+[D^u]_{\mathscr {V}_p^\beta }). \end{aligned}$$

### Proof

For $$(t,x) \in [0,1]\times \mathbb {T}$$ denote

$$V_t(x) :=\int _0^t \int _{\mathbb {T}} p_{t-r}(x,y) \sigma (u(r,y)) \xi (dy,dr).$$

By the triangle inequality

$$\begin{aligned} \Vert u - Pu_0\Vert _{C^{1/4 - \varepsilon /2, 1/2-\varepsilon }([0,1]\times \mathbb {T},L_p)} \le \,&\Vert D^u\Vert _{C^{1/4 - \varepsilon /2, 1/2-\varepsilon }([0,1]\times \mathbb {T},L_p)}\\&+ \Vert V\Vert _{C^{1/4 - \varepsilon /2, 1/2-\varepsilon }([0,1]\times \mathbb {T},L_p)}. \end{aligned}$$

But by Lemma 9.2, we know that $\Vert D^u\Vert _{C^{1/4, 1/2}([0,1]\times \mathbb {T},L_p)} \lesssim (1+\Vert b\Vert _{C^\alpha })(1+[D^u]_{\mathscr {V}_p^\beta })$ and it can be seen from the BDG inequality and by the heat kernel estimates (11.4) and (11.5) that $\Vert V\Vert _{C^{1/4 - \varepsilon /2, 1/2-\varepsilon }([0,1]\times \mathbb {T}, L_p)} \lesssim 1$, and thus the proof is finished. □

## Proof of the main result

### Theorem 10.1

(Uniqueness) Let Assumption 3.1 hold, let $\beta \in (\frac{1}{2} - \frac{\alpha }{4}, 1+\frac{\alpha }{4}]$ and suppose that $u^1,u^2$ are regularised solutions of (1.1) in the class $\mathscr {U}_2^{\beta }$. Then $u^1(t,x) = u^2(t,x)$ almost surely for all $(t,x) \in [0,1]\times \mathbb {T}$.

### Proof

Since $u^1,u^2 \in \mathscr {U}_2^\beta$, it also follows by Lemma 9.1 that $u^1,u^2 \in \mathscr {U}^\beta$. Thus Assumption 8.1 satisfied. Therefore by Lemma 8.6 we have for $p \in [2,\infty )$ that

$${[}u^1,u^2]_{\mathscr {S}_{p}^{1/2}} \le 0.$$

It follows by the above inequality that

$$\sup _{(t,x) \in [0,1]\times \mathbb {T}}\Vert u^1(t,x) - u^2(t,x)\Vert _{L_{p}} =0,$$

and the desired result follows. □

Let Assumption 3.1 hold. The rest of the section is concerned with proving the existence of regularised solutions in the class $\mathscr {U}^{1+\frac{\alpha }{4}}$. Let $(b^n)_{n \in \mathbb {N}} \subset C^\infty$ such that $b^n \rightarrow b$ in $C^\alpha$. Suppose that for all $n \in \mathbb {N}$, $u^n$ is the classical mild solution of the SPDE

10.1 $$\begin{aligned} (\partial _t - \Delta )u^n&= b^n(u^n) + \sigma (u^n)\xi , \qquad u^n(0,\cdot )&= u(0,\cdot ). \end{aligned}$$

We call $(u^n)_{n \in \mathbb {N}}$ the sequence of *approximate solutions*, and for $(t,x) \in [0,1]\times \mathbb {T}$ we define the corresponding *approximate drift term* and *approximate noise term* respectively by

$$\begin{aligned}D_t^{u^n}(x)&:= \int _0^t \int _{\mathbb {T}}p_{t-r}(x,y)b^n(u^n(r,y))dydr,\\ V^{u^n}_t(x)&:= \int _0^t \int _{\mathbb {T}}p_{t-r}(x,y)\sigma (u^n(r,y))\xi (dy,dr). \end{aligned}$$

By Lemma 9.1 we have for all p≥1, that

10.2 $$\begin{aligned} \sup _{n \in \mathbb {N}}[D^{u^n}]_{\mathscr {V}_p^{1+\alpha /4}} <\infty . \end{aligned}$$

### Lemma 10.2

(Convergence of the approximate drift and noise terms) Let Assumption 3.1 hold, and let $p \in [1,\infty )$ and $\varepsilon \in (0,\frac{1}{2})$. Then the sequences $(D^{u^n})_{n \in \mathbb {N}}$, $(V^{u^n})_{n \in \mathbb {N}}$ are convergent in $C^{\frac{1}{4}-\frac{\varepsilon }{2},\frac{1}{2} - \varepsilon }([0,1]\times \mathbb {T}, L_p)$.

### Proof

Assume without loss of generality that p>2. By Corollary 7.5 (with $\beta = 1+\frac{\alpha }{4}$), Lemma 8.6, and keeping in mind (10.2) , we have

10.3 $$\begin{aligned}&\sup _{(t,x) \in [0,1]\times \mathbb {T}}\Vert D_t^{u^n}(x) - D_t^{u^m}(x)\Vert _{L_p}\nonumber \\&\quad =\sup _{(t,x) \in [0,1]\times \mathbb {T}}\Big \Vert \int _0^t \int _{\mathbb {T}}p_{t-r}(x,y)\big (b^n(u^n(r,y)) - b^m(u^m(r,y))\big )dydr\Big \Vert _{L_p}\nonumber \\&\quad \lesssim \Vert b^n - b^m\Vert _{C^{\alpha -1}} + [u^n,u^m]_{\mathscr {S}^{1/2}_p[0,1]}\lesssim \Vert b^n- b^m\Vert _{C^\alpha } \longrightarrow 0 \end{aligned}$$

as $n \rightarrow \infty$. Moreover by Lemma 9.2 (with $\beta = 1+\frac{\alpha }{4}$) and by (10.2), we have that

10.4 $$\begin{aligned} \sup _{n \in \mathbb {N}}\Vert D^{u^n}\Vert _{C^{1/4,1/2}([0,1]\times \mathbb {T}, L_p)} <\infty . \end{aligned}$$

By (10.3), (10.4), and by a standard interpolation argument, we can see that $(D^{u^n})_{n \in \mathbb {N}}$ is Cauchy in $C^{\frac{1}{4}-\frac{\varepsilon }{2},\frac{1}{2} - \varepsilon }([0,1]\times \mathbb {T}, L_p)$.

We proceed with showing that the same is true for the sequence $(V^n)_{n \in \mathbb {N}}$. To this end note that by the BDG inequality, by the definition of the $\mathscr {S}_p^{1/2}$-bracket, and by Lemma 8.6 we have

10.5 $$\begin{aligned}&\sup _{(t,x) \in [0,1]\times \mathbb {T}}\Vert V_t^{u^n}(x) - V_t^{u^m}(x)\Vert _{L_p} \nonumber \\&\quad = \sup _{(t,x) \in [0,1]\times \mathbb {T}}\Big \Vert \int _0^t \int _{\mathbb {T}}p_{t-r}(x,y)\big (\sigma (u^n(r,y)) - \sigma (u^m(r,y))\big )\xi (dy,dr)\Big \Vert _{L_p}\nonumber \\&\quad \lesssim t^{1/4}\Vert u^n - u^m\Vert _{\mathbb {B}([0,1]\times \mathbb {T}, L_p)} \le [u^n,u^m]_{\mathscr {S}_p^{1/2}} \lesssim \Vert b^n - b^m\Vert _{C^{\alpha -1}} \longrightarrow 0 \end{aligned}$$

as $n,m \rightarrow \infty$. Let $\gamma \in (0,\varepsilon )$. Using the BDG inequality and the heat kernel estimates (11.4), (11.5), we can see that for all $n \in \mathbb {N}$, s,t∈[0,1], $x,\bar{x} \in \mathbb {T}$ the following estimates hold:

$$\begin{aligned} \Vert V_t^{u^n}(x) - V_t^{u^n}(\bar{x})\Vert _{L_p}^2&\lesssim \int _0^t \int _{\mathbb {T}}(p_{t-r}(x,y) - p_{t-r}(\bar{x},y))^2 dydr \lesssim |x-\bar{x}|^{1-2\gamma },\\ \Vert V_t^{u^n}(x) - V_s^{u^n}(x)\Vert _{L_p}^2&\lesssim \int _0^s \int _{\mathbb {T}}(p_{t-r}(x,y) - p_{s-r}(x,y))^2 dydr\\&\qquad + \int _s^t \int _{\mathbb {T}} p_{t-r}^2(x,y)dydr\\&\lesssim |t-s|^{1/2-\gamma }. \end{aligned}$$

Therefore we conclude that

10.6 $$\begin{aligned} \sup _{n \in \mathbb {N}} \Vert V^{u^n}\Vert _{C^{\frac{1}{4}-\frac{\gamma }{2}, \frac{1}{2}-\gamma }([0,1]\times \mathbb {T}, L_p)} <\infty . \end{aligned}$$

By (10.5), (10.6), and by a standard interpolation argument, we can see that $(V^{n})_{n \in \mathbb {N}}$ is also Cauchy in $C^{\frac{1}{4}-\frac{\varepsilon }{2},\frac{1}{2} - \varepsilon }([0,1]\times \mathbb {T}, L_p)$, and thus the proof is finished. □

Consistently with the above lemmas, we will thus denote

$$\begin{aligned} {D}^{\tilde{u}} :=\lim _{n \rightarrow \infty }D^{u^n} \quad \text {and}\quad V^{\tilde{u}} := \lim _{n \rightarrow \infty }V^{u^n}, \end{aligned}$$

where the limits are taken pointwise in $(t,x)\in [0,1]\times \mathbb {T}$, in probability. Moreover, it follows that for all $\varepsilon \in (0, 1/2)$ and $p \in [1, \infty )$ we have ${D}^{\tilde{u}},V^{\tilde{u}} \in C^{\frac{1}{4}-\frac{\varepsilon }{2} , \frac{1}{2}-\varepsilon }([0,1]\times \mathbb {T}, L_p)$ and

10.7 $$\begin{aligned} \lim _{n \rightarrow \infty } \Big ( || {D}^{\tilde{u}} -D^{u^n}\Vert _{C^{\frac{1}{4}-\frac{\varepsilon }{2}, \frac{1}{2}-\varepsilon }([0,1]\times \mathbb {T}, L_p)}+|| V^{\tilde{u}}-V^{u^n}\Vert _{C^{\frac{1}{4}-\frac{\varepsilon }{2}, \frac{1}{2}-\varepsilon }([0,1]\times \mathbb {T}, L_p)} \Big )=0 . \end{aligned}$$

Moreover for $(t,x) \in [0,1]\times \mathbb {T}$, we define

10.8 $$\begin{aligned} \tilde{u}(t,x) := P_t u_0(x) + D^{\tilde{u}}_t(x) + V^{\tilde{u}}_t(x). \end{aligned}$$

### Lemma 10.3

(Identification of $V^{\tilde{u}}$) Let Assumption 3.1 hold. The map $\tilde{u}: \Omega \times [0,1] \times \mathbb {T} \rightarrow \mathbb {R}$ defined in (10.8) is $\mathscr {P}\otimes \mathscr {B}(\mathbb {T})$-measurable and for all $(t,x) \in [0,1]\times \mathbb {T}$, we have with probability one

$$V^{\tilde{u}}_t(x) = \int _0^t \int _{\mathbb {T}}p_{t-r}(x,y)\sigma (\tilde{u}(r,y))\xi (dy,dr).$$

### Proof

Since for all $n \in \mathbb {N}$, the random field $u^n$ (which is a classically defined mild solution) is $\mathscr {P}\otimes \mathscr {B}(\mathbb {T})$-measurable, so is the limit $\tilde{u}$. By the definitions of $D^{\tilde{u}}$ and $V^{\tilde{u}}$ (see (10.7)), by Fatou’s lemma, and by the definition of $\tilde{u}$ (see (10.8)) we have for p≥2 that

$$\begin{aligned}&\Vert V_t^{\tilde{u}}(x) - \int _0^t \int _{\mathbb {T}}p_{t-r}(x,y)\sigma (\tilde{u}(r,y))\xi (dy,dr)\Vert _{L_p} \\&\quad \le \liminf _{n \rightarrow \infty }\Big \Vert \int _0^t \int _{\mathbb {T}}p_{t-r}(x,y)\sigma (u^n(r,y)) - \sigma (\tilde{u}(r,y))\xi (dy,dr)\Big \Vert _{L_p}\\&\quad \lesssim t^{1/4}\liminf _{n \rightarrow \infty }\Vert u^n - \tilde{u}\Vert _{\mathbb {B}([0,1]\times \mathbb {T}, L_p)}\\&\quad \lesssim \lim _{n \rightarrow \infty }\Vert D^{u^n} - D^{\tilde{u}}\Vert _{\mathbb {B}([0,1]\times \mathbb {T}, L_p)} + \lim _{n \rightarrow \infty }\Vert V^{u^n} - V^{\tilde{u}}\Vert _{\mathbb {B}([0,1]\times \mathbb {T}, L_p)}= 0, \end{aligned}$$

and thus the proof is finished. □

We proceed with verifying that the definition of $D^{\tilde{u}}$ is not an abuse of notation, i.e. that $D^{\tilde{u}}$ is indeed the drift of $\tilde{u}$ as prescribed in (3.1). To this end, we will first need to prove the following lemma.

### Lemma 10.4

Let Assumption 3.1 hold, and for $n \in \mathbb {N}$ define random fields $f^n: \Omega \times [0,1]\times \mathbb {T} \rightarrow \mathbb {R}$ by

10.9 $$\begin{aligned} f^n(t,x) :=D_t^{\tilde{u}}(x) - \int _0^t \int _{\mathbb {T}} p_{t-r}(x,y) b^n(\tilde{u}(r,y))dydr. \end{aligned}$$

Then for any $p \in [1,\infty )$ we have that $\Vert f^n\Vert _{C^{\frac{1}{4},\frac{1}{2}}([0,1]\times \mathbb {T}, L_p)} \longrightarrow 0$ as $n \rightarrow \infty$.

### Proof

To bound the sup norm, we note that by Fatou’s lemma, , Corollary 7.2 (with $g = b^m - b^n$) and Lemma 9.1, we have that

$$\begin{aligned}&\Vert f^n\Vert _{\mathbb {B}([0,1]\times \mathbb {T}, L_p)}\\&\quad = \sup _{(t,x) \in [0,T]\times \mathbb {T}}\Big \Vert {D}_t^{\tilde{u}}(x) - \int _0^t \int _{\mathbb {T}} p_{t-r}(x,y) b^n(\tilde{u}(r,y))dydr\Big \Vert _{L_p}\\&\quad \le \liminf _{m \rightarrow \infty }\sup _{(t,x) \in [0,T]\times \mathbb {T}}\Big \Vert \int _0^t \int _{\mathbb {T}}p_{t-r}\big (b^m(u^m(r,y)) - b^n(u^m(r,y))\big )dydr \Big \Vert _{L_p}\\&\quad \lesssim \liminf _{m \rightarrow \infty }\Vert b^m - b^n\Vert _{C^\alpha }(1+[D^{u^m}]_{\mathscr {V}_2^{1+\alpha /4}})(t-s)^{1 + \alpha /4}\lesssim \Vert b - b^n\Vert _{C^\alpha }, \end{aligned}$$

and thus

10.10 $$\begin{aligned} \lim _{n \rightarrow \infty }\Vert f^n\Vert _{\mathbb {B}([0,1]\times \mathbb {T}, L_p)} = 0. \end{aligned}$$

Next, we bound the spatial seminorm. Let $x, \bar{x} \in \mathbb {T}$. In the calculation below we will use the definitions of $D^{\tilde{u}}$ $\tilde{u}$, $f^n$ (see (10.7), (10.8), and (10.9)) and the continuity of the approximate drifts, Fatou’s lemma, Corollary 7.3 (with $g(x) = b^m(x) - b^n(x)$) and (10.2),

$$\begin{aligned}&\sup _{t \in [0,1]}\Vert f^n(t,x) - f^n(t,\bar{x})\Vert _{L_p}\\&\quad =\sup _{t \in [0,1]}\Big \Vert D_t^{\tilde{u}}(x) - D_t^{\tilde{u}}(\bar{x}) - \int _0^t \int _{\mathbb {T}}(p_{t-r}(x,y) - p_{t-r}(\bar{x},y))b^n(\tilde{u}(r,y))dydr\Big \Vert _{L_p}\\&\quad \le \sup _{t \in [0,1]}\liminf _{m \rightarrow \infty }\Big \Vert \int _0^t \int _{\mathbb {T}}\big (p_{t-r}(x,y) - p_{t-r}(\bar{x},y))\big (b^m(u^m(r,y)) -b^n(u^m(r,y))\big )dydr\Big \Vert _{L_p}\\&\quad \lesssim \liminf _{m \rightarrow \infty }\Vert b^m - b^n\Vert _{C^\alpha }(1+[D^{u^m}]_{\mathscr {V}_{2}^{1+\alpha /4}})|x-\bar{x}|^{1/2} \lesssim \Vert b - b^n\Vert _{C^\alpha }|x-\bar{x}|^{1/2}. \end{aligned}$$

Therefore

10.11 $$\begin{aligned} \lim _{n \rightarrow \infty }\sup _{t \in [0,1]}[f^n(t,\cdot )]_{C^{1/2}(\mathbb {T}, L_p)} = 0. \end{aligned}$$

Finally, note that for s,t∈[0,1] we have by Fatou’s lemma, , Corollary 7.2 (with $g = b^m - b^n$), and Lemma 9.1, that

$$\begin{aligned}&\sup _{x \in \mathbb {T}}\Vert f^n(t,\cdot ) - P_{t-s}f^n(s,x)\Vert _{L_{p,\infty }^{\mathscr {F}_s}}\\&\quad = \sup _{x \in \mathbb {T}}\Big \Vert D_t^{\tilde{u}}(x) - \int _0^t \int _{\mathbb {T}}p_{t-r}(x,y)b^n(\tilde{u}(r,y))dydr\\&\qquad - P_{t-s}\Big (D_s^{\tilde{u}}(\cdot ) -\int _0^s \int _{\mathbb {T}}p_{t-r}(\cdot ,y)b^n(\tilde{u}(r, y))dydr \Big )(x)\Big \Vert _{L_{p,\infty }^{\mathscr {F}_s}}\\&\quad \le \sup _{x \in \mathbb {T}}\liminf _{m \rightarrow \infty }\Big \Vert \int _s^t \int _{\mathbb {T}}p_{t-r}(x,y)(b^m(u^m(r,y)) - b^n(u^m(r,y)))dydr \Big \Vert _{L_{p,\infty }^{\mathscr {F}_s}}\\&\quad \lesssim \liminf _{m \rightarrow \infty }\Vert b^m - b^n\Vert _{C^\alpha }(1+[D^{u^m}]_{\mathscr {V}_{2}^{1+\alpha /4}})(t-s)^{1 + \alpha /4}\\&\quad \lesssim \Vert b- b^n\Vert _{C^\alpha }(t-s)^{1 + \alpha /4}. \end{aligned}$$

It follows that

10.12 $$\begin{aligned} \lim _{n \rightarrow \infty }[f^n]_{\mathscr {V}_p^{1+\alpha /4}} = 0. \end{aligned}$$

By (10.10), (10.11), (10.12), and by Lemma 11.10 the proof is finished. □

### Corollary 10.5

($D^{\tilde{u}}$is the drift of $\tilde{u}$) Let Assumption 3.1 hold. Then the pair $(\tilde{u}, D^{\tilde{u}})$ satisfies the condition (3.1) from Definition 3.2, that is for any sequence $(b^n)_{n \in \mathbb {N}}\subset C^\infty$ such that $b^n \rightarrow b$ in $C^\alpha$, we have

$$\sup _{(t,x) \in [0,T]\times \mathbb {T}}\Big |D_t^{\tilde{u}}(x) - \int _0^t \int _{\mathbb {T}} p_{t-r}(x,y) b^n(\tilde{u}(r,y))dydr\Big | \longrightarrow 0$$

in probability as $n \rightarrow \infty$.

### Theorem 10.6

(Existence) Let Assumption 3.1 hold. Then the process $\tilde{u}$ is a regularised solution of (1.1) in the class $\mathscr {U}^{1 + \alpha /4}$.

### Proof

The measurability of $\tilde{u}$ was shown in Lemma 10.3. Next, by the definition of $\tilde{u}$ and by Lemma 10.2 we have that

$$\tilde{u} - P_\cdot u_0 \in C^{1/4 - \varepsilon , 1/4 - \varepsilon /2}([0,1]\times \mathbb {T}, L_p)$$

for p≥1 and for any ε>0. Therefore by Kolmogorov’s continuity theorem, the random field $\tilde{u}(t,x) -P_tu(0,\cdot )(x)$ is continuous in (*t*, *x*). So noting that $P_tu(0,x)$ is also continuous in (*t*, *x*), it follows that $\tilde{u}(t,x)$ is continuous in (*t*, *x*). Note moreover that by Corollary 10.5, the pair $(\tilde{u}, {D}^{\tilde{u}})$ satisfies (3.1). Finally, we observe that by the definition of $\tilde{u}$ and by Lemma 10.3 the integral equation (3.2) is satisfied. Therefore it is clear that $\tilde{u}$ is a regularised solution of (1.1). Moreover for all p≥1 we have

$${[}D^{\tilde{u}}]_{\mathscr {V}_p^{1+\alpha /4}} \le \liminf _{n \rightarrow \infty }[D^{u^n}]_{\mathscr {V}_p^{1+\alpha /4}} \le \sup _{n \in \mathbb {N}}[D^{u^n}]_{\mathscr {V}_p^{1+\alpha /4}} <\infty ,$$

where the last inequality holds by (10.2). Therefore $\tilde{u} \in \mathscr {U}^{1 + \alpha /4}$, and the proof is finished. □

## Data Availability

No datasets were generated or analysed during the current study.

## Appendix

### Proposition 11.1

For any $\gamma \in [0,1]$ there exists a constant N=N(γ)>0 such that for all t∈[0,1] and $x,\bar{x},y \in \mathbb {T}$ we have

11.1 $$\begin{aligned} |p_t(x,y) -p_t(\bar{x},y)| \le N|x-\bar{x}|^\gamma t^{-\gamma /2}\big (p_{2t}(x,y) + p_{2t}(\bar{x},y)\big ). \end{aligned}$$

Moreover for any $\gamma ,\beta \in [0,1]$ with $\gamma \le \beta$ there exists a constant $N = N(\gamma ,\beta )>0$ such that for all $(s,t) \in [0,1]_{\le }^2$ and $x,\bar{x} \in \mathbb {T}$ and for all $f \in C^\gamma (\mathbb {T})$ we have

11.2 $$\begin{aligned} |P_t f(x) - P_sf(\bar{x})| \le N\Vert f\Vert _{C^\gamma }(|x-\bar{x}|^\beta + |t-s|^{\beta /2})s^{(\gamma -\beta )/2}. \end{aligned}$$

The first inequality of the lemma above is taken from the proof of [2, Lemma C2], while the second inequality can be found in [6].

### Proposition 11.2

For any $\varepsilon \in (0,1]$ there exists a constant N=N(ε)>0 such that for all $(s,t) \in [0,1]_{\le }^2$, the following inequalities hold:

11.3 $$\begin{aligned} \int _{\mathbb {T}}|p_{t}(x,y) - p_t(\bar{x},y)|dy&\le N|x-\bar{x}|^\varepsilon t^{-\varepsilon /2} ,\end{aligned}$$

11.4 $$\begin{aligned} \int _0^t \int _{\mathbb {T}}|p_{t-r}(x,y)- p_{t-r}(\bar{x},y)|^2 dy dr&\le N |x-\bar{x}|^{1-\varepsilon }t^{\varepsilon /2} ,\end{aligned}$$

11.5 $$\begin{aligned} \int _0^s \int _{\mathbb {T}} |p_{t-r}(x,y) - p_{s-r}(x,y)|^2 dydr&\le {N} |t-s|^{1/2 - \varepsilon /2}. \end{aligned}$$

The inequality (11.3) can be found in Lemma C2 of ([2]). The inequality (11.4) can be proven by using (11.1) and (11.5) follows from [13, B.1.7, p. 368] (therein it is stated for the heat kernel on $\mathbb {R}$ but it can be directly applied to the heat kernel on the torus too due to (2.4)).

### Lemma 11.3

For every $\gamma \in (1,2]$ there exists a constant N(γ)>0 such that for all t∈[0,1],

$$\int _0^t \int _{\mathbb {T}} p_{t-r}^\gamma (x,y) e^{-\lambda (t-r)} dydr \le \frac{N}{\sqrt{\lambda }}.$$

### Proof

The left-hand-side is controlled by

$$\begin{aligned} \int _0^t (t-r)^{-1/2(\gamma -1)} e^{-\lambda (t-r)}dr&\le \int _0^t \frac{1}{\sqrt{t-r}}e^{-\lambda (t-r)}dr = \frac{2}{\sqrt{\lambda }}\int _0^{\sqrt{\lambda t}} e^{-\theta ^2} d\theta \\&=\frac{ \sqrt{\pi }}{\sqrt{\lambda }} \text {erf}(\sqrt{\lambda t})\le \frac{\sqrt{\pi }}{\sqrt{\lambda }} \end{aligned}$$

where we used the change of variables $\theta := \sqrt{\lambda }(t-r)^{1/2}$ and the fact that $|\text {erf}(\cdot )| \le 1$. □

### Lemma 11.4

(A Grönwall-type inequality) Fix s≥0. Let $C \in \mathbb {B}([s,1],\mathbb {R})$ be a non-decreasing function and let $f : [s,1] \times \mathbb {T} \rightarrow [0, \infty )$ be a bounded function. Suppose that there exists $\gamma \in (1,2]$ and $N_0 \ge 0$ such that for all t∈[s,1] and $x \in \mathbb {T}$ we have

$$ f(t,x) \le C(t)+N_0\int _s^t \int _{\mathbb {T}} p_{t-r}^\gamma (x,y)f(r,y) dy dr. $$

There exists a constant $N=N(\gamma , N_0)$ such that for all t∈[s,1] we have

$$\sup _{x \in \mathbb {T}}f(t,x) \le NC(t).$$

### Proof

Let λ>0 and consider the non-decreasing function of time $m: [s,1] \rightarrow \mathbb {R}$, that is given by

$$m_t := \sup _{s \le r \le t} \sup _{x \in \mathbb {T}}\big (f(r,x) e^{-\lambda r}\big ).$$

Then, for all t∈[s,1] we have

$$f(t,x) \lesssim C(t) + \int _s^t \int _{\mathbb {T}} p_{t-r}^\gamma (x,y)m_r e^{\lambda r} dy dr,$$

where used the definition of *m*. Multiplying both sides by $e^{-\lambda t}$ and noting that $m_r \le m_t$ for r≤t gives

$$f(t,x) e^{-\lambda t} \lesssim C(t) e^{-\lambda t} + m_t \int _s^t \int _{\mathbb {T}} p_{t-r}^\gamma (x,y) e^{-\lambda (t-r)} dy dr.$$

Let T∈[s,1]. Using Lemma 11.3 to estimate the second term, and taking supremum over $(t,x) \in [s,T]\times \mathbb {T}$ we get

$$m_T \lesssim C(T) + \frac{m_T}{\sqrt{\lambda }}.$$

Choosing λ to be sufficiently large, we get that $m_T \lesssim C(T)$. Since, T∈[s,1] was arbitrary, the result follows by the definition of *m*. □

### Lemma 11.5

(A corollary of Hölder’s inequality) Let $\gamma \in \{1,2\}$ and $p \in (2, \infty )$. There exists a constant N=N(γ,p)>0, such that for all $(s,t) \in [0,1]_{\le }^2$, and all measurable $f:[s,t] \times \mathbb {R} \rightarrow [0, \infty )$, we have

11.6 $$\begin{aligned}&\Big (\int _s^t\int _{\mathbb {T}} |p_{t-r}(x,y)|^2 f(r,y) dy dr\Big )^{p} \nonumber \\&\qquad \le N (t-s)^{ \frac{p+\gamma -3}{2}}\int _s^t \int _{\mathbb {T}} |p_{t-r}(x,y)|^\delta f^p(r,y) dy dr. \end{aligned}$$

### Proof

By Hölder’s inequality, the left-hand-side of (11.6) is bounded by

$$\begin{aligned} \Big (\int _s^t \int _{\mathbb {T}} |p_{t-r}(x,y)|^{(2 - \frac{\gamma }{p})\frac{p}{p-1}} dy dr\Big )^{p-1} \int _s^t \int _{\mathbb {T}}| p_{t-r}(x,y)|^\gamma f^p(r,y) dy dr. \end{aligned}$$

Moreover using the results $\Vert p_t\Vert _{\mathbb {B}(\mathbb {T})} \lesssim t^{-1/2}$ and $\Vert p_t\Vert _{L_1(\mathbb {T})} =1$ to interpolate (we have $1 \le {(2 - \frac{\gamma }{p})\frac{p}{p-1}} < \infty$), we can see that the first factor is bounded by

$$\begin{aligned} \Big (\int _s^t (t-r)^{-\frac{1}{2}\big ((2 - \frac{\gamma }{p})\frac{p}{p-1} -1\big )} dr\Big )^{p-1}&\lesssim (t-s)^{\Big (-\frac{1}{2}\big ((2 - \frac{\gamma }{p})\frac{p}{p-1} -1\big ) + 1\Big )(p-1)}\\&= (t-s)^{\frac{p+\gamma -3}{2}},\end{aligned}$$

where we have used that ${-\frac{1}{2}\Big ((2 - \frac{\gamma }{p})\frac{p}{p-1} -1\Big )} > -1$ for $\gamma \in \{1,2\}$ and p>2. Hence, the proof is finished. □

### Lemma 11.6

(Conditional BDG inequality) Let p≥2, $(s,t) \in [0,1]^2_{\le }$. Let *V* be a separable Hilbert space and let $X:\Omega \ \times [0,1]\times \mathbb {T} \rightarrow V$ be a $\mathscr {P}\otimes \mathscr {B}(\mathbb {T})$-measurable *V*-valued random field such that

$$\begin{aligned} \mathbb {E}\Big ( \int _s^t \int _{\mathbb {T}}\Vert X(r, y)\Vert _V^2 \, dy dr \Big )^{p/2}< \infty . \end{aligned}$$

Then, there exists a constant $C_p$, depending only on *p*, such that

11.7 $$\begin{aligned} \mathbb {E}^s \Big \Vert \int _s^t \int _{\mathbb {T}} X(r,y)\xi (dy,dr)\Big \Vert _{V}^p\le C_p \mathbb {E}^s \Big (\int _s^t \int _{\mathbb {T}} \Vert X(r,y) \Vert _V^2 dydr \Big )^{p/2}. \end{aligned}$$

Assume further that

11.8 $$\begin{aligned} \int _s^t \int _{\mathbb {T}}\Vert X(r, y)\Vert _{L_p(\Omega ; V)}^2 \, dy dr < \infty . \end{aligned}$$

Then, the conditional expectation $\mathbb {E}^s\Vert X(r,y)\Vert _V^p$ exists for almost every $(r,y) \in [0,1] \times \mathbb {T})$, it can be chosen so that the map $(\omega , r, y) \mapsto \mathbb {E}^s\Vert X(r,y)\Vert _V^p(\omega )$ is $\mathcal {F}_s\otimes \mathcal {B}([0,1]) \otimes \mathcal {B}(\mathbb {T})$-measurable, and the following holds with probability one

11.9 $$\begin{aligned}&\Big \Vert \Big \Vert \int _s^t \int _{\mathbb {T}} X(r,y) \xi (dy,dr)\Big \Vert _{V}\Big \Vert _{L_p|\mathscr {F}_s}^2\le C_p \int _s^t \int _{\mathbb {T}} \Vert \Vert X(r,y)\Vert _{V}\Vert _{L_p|\mathscr {F}_s}^2dydr. \end{aligned}$$

### Proof

By applying (2.2) to $F= \textbf{1}_{B}\textbf{1}_{[s,t]}X$, where $B \in \mathcal {F}_s$ is arbitrary, we get

$$\begin{aligned} \mathbb {E}\Big ( \textbf{1}_B \Big \Vert \int _s^t \int _{\mathbb {T}} X(r,y)\xi (dy,dr) \Big \Vert _{V}^p\Big ) \le C_p \mathbb {E}\Big ( \textbf{1}_B \Big (\int _s^t \int _{\mathbb {T}} \Vert X(r,y) \Vert _V^2 dydr \Big )^{p/2} \Big )< \infty . \end{aligned}$$

In particular, by choosing B=Ω, we have that the conditional expectation at the right hand side of (11.7) exists. Moreover, since $B \in \mathcal {F}_s$ was arbitrary, (11.7) follows by definition of the conditional expectation.

Next, the fact that $\mathbb {E}^s\Vert X(r,y)\Vert _V^p$ exists for almost every $(r,y) \in [0,1] \times \mathbb {T})$ follows from (11.8). The fact that it can be chosen to be $\mathcal {F}_s\otimes \mathcal {B}([0,1]) \otimes \mathcal {B}(\mathbb {T})$-measurable is shown in [18].

We now move to (11.9), which follows by conditional Minkowski’s inequality, but we include its proof for the convenience of the reader. We first show that for q≥1, a σ-algebra $\mathcal {G}\subset \mathcal {F}$ and a bounded $\mathcal {F}\otimes \mathcal {B}([0,1]) \otimes \mathcal {B}(\mathbb {T})$-measurable map $Z : \Omega \times [0,1] \times \mathbb {T} \rightarrow [0, \infty )$, we have with probability one

11.10 $$\begin{aligned} \Big \Vert \int _0^1 \int _\mathbb {T} Z(s, y)\,dy ds \Big \Vert _{L_q| \mathcal {G}} \le \int _0^1 \int _\mathbb {T} \Vert Z(s, y)\Vert _{L_q| \mathcal {G}} \,dy ds. \end{aligned}$$

By the way, the integrand on the right hand side is (can be chosen) jointly measurable in (ω,t,x) by [18] again, hence, everything is well defined in the above inequality. To show that the inequality holds, we can take non-negative $Z_n$, such that $\lim _nZ_n= Z$ and $Z_n \le Z$ almost everywhere, and have the form

$$\begin{aligned} Z_n(t,x)= \sum _{i=1}^{k(n)}G_i\textbf{1}_{A_i}(t)\textbf{1}_{B_i}(x), \end{aligned}$$

where $G_i$ are bounded non-negative random variables, and $A_i$ and $B_i$ are Borel subsets of [0, 1] and $\mathbb {T}$, respectively. The existence of such sequence follows from monotone class theorem. By the triangle inequality for conditional expectations, it follows that

$$\begin{aligned} \Big \Vert \int _0^1 \int _\mathbb {T} Z_n(s, y)\,dy ds \Big \Vert _{L_q| \mathcal {G}}&\le \int _0^1 \int _\mathbb {T} \Vert Z_n(s, y)\Vert _{L_q| \mathcal {G}} \,dy ds\\  &\le \int _0^1 \int _\mathbb {T} \Vert Z(s, y)\Vert _{L_q| \mathcal {G}} \,dy ds. \end{aligned}$$

From this, (11.10) follows by dominated convergence and conditional Fatou’s lemma. Furthermore, by standard truncation argument, (11.10) can be extended to all measurable maps *Z* satisfying

11.11 $$\begin{aligned} \int _0^1 \int _\mathbb {T} \Vert Z(s, y)\Vert _{L_q}\,dy ds < \infty . \end{aligned}$$

Next, we can apply (11.10) with $Z(r,y)= \textbf{1}_{[s,t]}(r)\Vert X(r,y)\Vert _V^2$, q=p/2, and $\mathcal {G}=\mathcal {F}_s$, to get

$$\begin{aligned} \Big \Vert \int _s^t \int _{\mathbb {T}} \Vert X(r,y) \Vert _V^2 dydr \Big \Vert _{L_{p/2}| \mathcal {F}_s} \le \int _s^t \int _{\mathbb {T}} \Vert \Vert X(r,y) \Vert _V \Vert ^2_{L_{p}| \mathcal {F}_s} dydr, \end{aligned}$$

which combined with (11.7) leads to (11.9). □

### Lemma 11.7

Suppose that $f: \mathbb {R} \rightarrow \mathbb {R}$ is twice differentiable. Then for $\phi _1,\dots , \phi _4 \in \mathbb {R}$ we have

11.12 $$\begin{aligned}&f(\phi _1) - f(\phi _2) - f(\phi _3) + f(\phi _4) \nonumber \\&\quad = \int _0^1 \int _0^1 (\phi _1- \phi _2)(\theta (\phi _1 - \phi _3) + (1-\theta )(\phi _2 - \phi _4))\nabla ^2 f(\Theta _1(\theta ,\eta ))d\eta d \theta \nonumber \\&\qquad + (\phi _1 - \phi _2 - \phi _3 + \phi _4)\int _0^1 \nabla f(\Theta _2(\theta ))d\theta \end{aligned}$$

where $\Theta _1(\theta ,\eta )$ and $\Theta _2(\theta )$ are the convex combinations of $\phi _1,\dots , \phi _4$ given by

$$\begin{aligned} \Theta _1(\theta ,\eta )&:= \eta (\theta \phi _1 + (1-\theta )\phi _2) + (1-\eta )(\theta \phi _3 + (1-\theta ) \phi _4),\\ \Theta _2(\theta )&:= \theta \phi _3 + (1-\theta ) \phi _4. \end{aligned}$$

Moreover

11.13 $$\begin{aligned}&|f(\phi _1) - f(\phi _2) - f(\phi _3) + f(\phi _4)| \nonumber \\&\qquad \le \Vert f\Vert _{C^2}|\phi _1 - \phi _2||\phi _1 - \phi _3| + \Vert f\Vert _{C^1}|\phi _1 - \phi _2 - \phi _3 + \phi _4|. \end{aligned}$$

### Proof

We begin by proving (11.12). Using the notation

$$\begin{aligned} \phi _{1,2}^\theta := \theta \phi _1 + (1-\theta )\phi _2, \qquad \phi _{3,4}^\theta := \theta \phi _3 + (1-\theta )\phi _4, \end{aligned}$$

the expression $f(\phi _1) - f(\phi _2) - f(\phi _3) + f(\phi _4)$ can be rewritten as

$$\begin{aligned}&(\phi _1 - \phi _2)\int _0^1 \nabla f(\phi _{1,2}^\theta ) d\theta - (\phi _3 - \phi _4)\int _0^1 \nabla f(\phi _{3,4}^\theta ) d\theta \\&\quad = (\phi _1 - \phi _2)\int _0^1\Big (\nabla f(\phi _{1,2}^\theta ) -\nabla f(\phi _{3,4}^\theta )\Big )d\theta \\&\qquad + (\phi _1 - \phi _2 - \phi _3 + \phi _4) \int _0^1 \nabla f(\phi _{3,4}^\theta ) d \theta . \end{aligned}$$

The second term is exactly as desired, and the first term can be written as

$$(\phi _1 - \phi _2)\int _0^1(\phi _{1,2}^\theta - \phi _{3,4}^\theta )\int _0^1 \nabla ^2 f(\eta \phi _{1,2}^\theta + (1-\eta )\phi _{3,4}^\theta ) d\eta d\theta $$

which is indeed the first term of the desired expression. Hence (11.12) is proven. To prove (11.13), we set

$$\delta _{i,j} := \phi _j - \phi _i$$

and we note that $f(\phi _1) - f(\phi _2) - f(\phi _3) + f(\phi _4)$ can be written as

$$\begin{aligned}&f(\phi _1)- f(\phi _1 + \delta _{1,2}) - f(\phi _3) + f(\phi _3 + \delta _{1,2}) - f(\phi _{3} + \delta _{1,2}) + f(\phi _3 + \delta _{3,4})\\&\quad = -\delta _{1,2}\int _0^1 \nabla f(\phi _1 + \theta \delta _{1,2}) d\theta + \delta _{1,2}\int _0^1 \nabla f(\phi _3 + \theta \delta _{1,2}) d\theta \\&\qquad +(\delta _{3,4} - \delta _{1,2}) \int _0^1 \nabla f(\phi _3 + \theta \delta _{3,4} + (1-\theta )\delta _{1,2}) d \theta \\&\quad = \delta _{1,2}\delta _{1,3}\int _0^1 \int _0^1 \nabla ^2 f(\eta \phi _3 + (1-\eta )\phi _1 +\theta \delta _{1,2}) d\theta d \eta \\&\qquad + (\delta _{3,4} - \delta _{1,2})\int _0^1 \nabla f(\phi _3 + \theta \delta _{3,4} + (1-\theta )\delta _{1,2})d\theta . \end{aligned}$$

Hence (11.13) follows as well. □

The following result is taken from [15].

### Proposition 11.8

Let $\gamma \in \mathbb {R} \setminus \mathbb {Z}$. There exists a constant N=N(γ) such that for all $f \in C^{\gamma }(\mathbb {R}^d)$ we have

$$\Vert (1-\Delta )^{-1} f \Vert _{C^{\gamma + 2}(\mathbb {R}^d)} \le N \Vert f\Vert _{C^\gamma (\mathbb {R}^d)}.$$

### Lemma 11.9

Let $p\in [1,\infty )$, γ>0, and let $f \in \mathscr {V}_p^\gamma$. Then for all $0\le S \le Q \le T \le 1$ we have

11.14 $$\begin{aligned} [f]_{\mathscr {V}_p^\gamma [S,T]} \le 2[f]_{\mathscr {V}_p^\gamma [S,Q]} + 2[f]_{\mathscr {V}_p^\gamma [Q,T]}. \end{aligned}$$

Consequently, for any integer K≥2 we have

11.15 $$\begin{aligned} [f]_{\mathscr {V}_p^\gamma } \lesssim 2^K\sum _{i=0}^{K-1} [f]_{\mathscr {V}_p^{\gamma }[\frac{i}{K},\frac{i+1}{K}]}. \end{aligned}$$

### Proof

For $$(s,t) \in [0,1]_{\le }^2$$ define

$$A(s,t) := \sup _{x \in \mathbb {T}}\Vert f_t(x) - P_{t-s}f_s(x)\Vert _{L_{p, \infty }^{\mathscr {F}_s}}.$$

For $(s,t) \in [S,Q]_{\le }^2 \cup [Q,T]_{\le }^2$, we clearly have

11.16 $$\begin{aligned} A(s,t) \le [f]_{\mathscr {V}_p^\gamma [s,t]}(t-s)^\gamma \le \Big ( [f]_{\mathscr {V}_p^\gamma [S,Q]} + [f]_{\mathscr {V}_p^\gamma [Q,T]}\Big )(t-s)^\gamma . \end{aligned}$$

We also need to check what happens in the case when Q∈(s,t). Then we write

$$\begin{aligned} A(s,t)&\le \sup _{x \in \mathbb {T}}\Vert f_t(x) - P_{t-Q}f_Q(x)\Vert _{L_{p,\infty }^{\mathscr {F}_s}} + \sup _{x \in \mathbb {T}}\Vert P_{t-Q}f_Q(x) - P_{t-s}f_s(x)\Vert _{L_{p,\infty }^{\mathscr {F}_s}}\\&= B(s,t) + C(s,t). \end{aligned}$$

Note that as s≤Q, we have $\Vert \cdot \Vert _{L_{p}|\mathscr {F}_s} \le \Vert \Vert \cdot \Vert _{L_p |\mathscr {F}_Q}\Vert _{L_p|\mathscr {F}_s} \le \Vert \cdot \Vert _{L_{p,\infty }^Q}$, and thus

$$\begin{aligned} B(s,t) \le \sup _{x \in \mathbb {T}}\Vert f_t(x) - P_{t-Q}f_Q(x)\Vert _{L_{p,\infty }^Q} = A(Q,t). \end{aligned}$$

Moreover using that s≤Q, we have

$$\begin{aligned} C(s,t)&= \sup _{x \in \mathbb {T}}\Vert P_{t-Q}\big (f_Q - P_{Q-s}f_s \big )(x)\Vert _{L_{p,\infty }^{\mathscr {F}_s}} \\&\le \sup _{x \in \mathbb {T}}\Vert f_Q(x) - P_{Q-s}f_s (x)\Vert _{L_{p,\infty }^{\mathscr {F}_s}} = A(s,Q) . \end{aligned}$$

By the above bounds on *B* and *C*, we conclude that

11.17 $$\begin{aligned} A(s,t) \le A(s,Q) + A(Q,t) \le \Big ( [f]_{\mathscr {V}_p^\gamma [S,Q]} + [f]_{\mathscr {V}_p^\gamma [Q,T]}\Big )(t-s)^\gamma . \end{aligned}$$

By adding up the bounds (11.16) and (11.17), we can see that for all $(s,t) \in [S,T]_{\le }^2$ and Q∈[S,T], we have

$$\begin{aligned} A(s,t) \le 2\Big ( [f]_{\mathscr {V}_p^\gamma [S,Q]} + [f]_{\mathscr {V}_p^\gamma [Q,T]}\Big )(t-s)^\gamma , \end{aligned}$$

from which the desired result follows. □

### Lemma 11.10

Let $\alpha \in (-1,0)$ and $p \in [1,\infty )$. There exists a constant N=N(p,α)>0 such that for $f\in \mathscr {V}_p^{1/4}\cap C^{0,1/2}([0,1]\times \mathbb {T}, L_p)$ we have

$$\begin{aligned} \Vert f\Vert _{C^{1/4, 1/2}([0,1]\times \mathbb {T}, L_p)} \le N[f]_{\mathscr {V}_p^{1/4}} + N\Vert f\Vert _{C^{0,1/2}([0,1]\times \mathbb {T}, L_p)}. \end{aligned}$$

### Proof

We decompose the space–time Hölder norm to the sup norm, and spatial and temporal seminorms as follows:

11.18 $$\begin{aligned}&\Vert f\Vert _{C^{1/4,1/2}([0,1]\times \mathbb {T}, L_p)} \nonumber \\&\le \Vert f\Vert _{\mathbb {B}([0,1]\times \mathbb {T}, L_p)} + \sup _{x \in \mathbb {T}}[f(\cdot , x)]_{C^{1/4}([0,1])} + \sup _{t \in [0,1]}[f(t,\cdot )]_{C^{1/2}(\mathbb {T}, L_p)}. \end{aligned}$$

To bound the temporal seminorm, note that for $(s,t) \in [0,1]_{\le }^2$ we have

11.19 $$\begin{aligned} \Vert f(t,\cdot ) -f(s,\cdot )\Vert _{\mathbb {B}(\mathbb {T}, L_p)}&\le \Vert f(t,\cdot ) - P_{t-s}f(s,\cdot )\Vert _{\mathbb {B}(\mathbb {T}, L_p)} \nonumber \\&\qquad + \Vert P_{t-s}f(s,\cdot ) - f(s,\cdot )\Vert _{\mathbb {B}(\mathbb {T}, L_p)} \nonumber \\&=: A(s,t) + B(s,t). \end{aligned}$$

Since $\Vert \cdot \Vert _{L_p} \le \Vert \cdot \Vert _{L_{p,\infty }^{\mathscr {F}_s}}$, it follows that

$$\begin{aligned} A(s,t) \le [f]_{\mathscr {V}_p^{1/4}[s,t]}(t-s)^{1/4}. \end{aligned}$$

Moreover by a standard heat kernel estimate

$$\begin{aligned} B(s,t)&\lesssim \Vert f(s,\cdot )\Vert _{C^{1/2}(\mathbb {T}, L_p)}(t-s)^{1/4}. \end{aligned}$$

By putting the above bounds on *A* and *B* into (11.19), we can see that

$$\begin{aligned} \sup _{x \in \mathbb {T}}[f(\cdot ,x)]_{C^{1/4}([0,1], L_p)} \lesssim [f]_{\mathscr {V}_p^{1/4}[s,t]} + \Vert f\Vert _{C^{0,1/2}([0,1]\times \mathbb {T}, L_p)}. \end{aligned}$$

Using this bound on the second term of (11.18) finishes the proof. □

In the next lemma, we show a Markov-type property which will be used often. It is very standard but the proof is included for the convenience of the reader. Recall that $C(\mathbb {T})$ denotes the collection of continuous functions $f:\mathbb {T} \rightarrow \mathbb {R}$, and it is equipped with the sup-norm $\Vert \cdot \Vert _{\mathbb {B}}$. The topology induced by this norm generates the Borel σ-algebra $\mathscr {B}(C(\mathbb {T}))$ which coincides with the cylindrical σ-algebra. Moreover, recall that since $C(\mathbb {T})$ is separable, the notions of measurable, weakly measurable, and strongly measurable $C(\mathbb {T})$-valued maps on Ω coincide. In addition, a continuous random field $u : \Omega \times \mathbb {T} \rightarrow {\mathbb {R}}$ is actually a $C(\mathbb {T})$-valued random variable.

### Lemma 11.11

Let s∈[0,1]; $b,\sigma \in C^{1}(\mathbb {R})$; $M \in \mathbb {N}$; $(Z_i)_{i=1}^M \subset L_2(\Omega , \mathscr {F}_s, \mathbb {P}; C(\mathbb {T}))\cap \mathbb {B}(\mathbb {T}, L_2(\Omega ))$; and let $\phi ^{Z,s},\phi ^Z$ be defined as in (5.29)-(5.31). Let *f* be a continuous function on $\mathbb {R}^M$ which has polynomial growth. For t∈[s,1], and $x \in \mathbb {T}$, define $g:(C(\mathbb {T}))^M \rightarrow \mathbb {R}$ by

$$ g(z_1,\dots ,z_M) := \mathbb {E}f\big (\phi ^{z_1}(t-s,x),\dots , \phi ^{z_M}(t-s,x) \big ). $$

Then, for i=1,⋯,M and $Z_i \in L_2(\Omega , \mathcal {F}_s, \mathbb {P}; C(\mathbb {T}))\cap \mathbb {B}(\mathbb {T}, L_2(\Omega ))$, we have

11.20 $$\begin{aligned} \mathbb {E}^s f\big (\phi ^{Z_1,s}(t,x),\dots , \phi ^{Z_M,s}(t,x)\big ) = g(Z_1,\dots , Z_M). \end{aligned}$$

### Proof

Suppose first that the $Z_i$ are simple random variables of the form

11.21 $$\begin{aligned} Z_i = \sum _{k=1}^K h_{k,i} \textbf{1}_{E_k} \end{aligned}$$

where $K \in \mathbb {N}$, $(h_{k,i})_{k=1}^K \subset C(\mathbb {T})$ and $(E_k)_{k=1}^K \subset \mathcal {F}_s$ is a partition of Ω. In this case, we have for $(t,x) \in [s,1]\times \mathbb {T}$ that

$$\begin{aligned} \mathbb {E}^s&f(\phi ^{Z_1,s}(t,x), \dots , \phi _{s,t}^{Z_M,s}(t,x))\\&= \mathbb {E}^sf\Big (\sum _{k=1}^K \textbf{1}_{E_k}\phi ^{h_{k,1},s}(t,x), \dots , \sum _{k=1}^K \textbf{1}_{E_k}\phi ^{h_{k,M},s}(t,x)\Big )\\&= \mathbb {E}^s \sum _{k=1}^K \textbf{1}_{E_k}f(\phi ^{h_{k,1},s}(t,x),\dots , \phi ^{h_{k,M},s}(t,x))\\&= \sum _{k=1}^K \textbf{1}_{E_k}\mathbb {E}^s f(\phi ^{h_{k,1},s}(t,x),\dots , \phi ^{h_{k,M},s}(t,x))\\&= \sum _{k=1}^K \textbf{1}_{E_k}\mathbb {E}f(\phi ^{h_{k,1}}(t-s,x),\dots , \phi ^{h_{k,M}}(t-s,x))\\&= \sum _{k=1}^K \textbf{1}_{E_k}g(h_{k,1},\dots ,h_{k,M})= g(Z_1,\dots ,Z_M), \end{aligned}$$

which shows (11.20). For the general case, since $Z_i \in L_2(\Omega , \mathcal {F}_s, \mathbb {P}; C(\mathbb {T}))$, for i=1,⋯,M there exist sequences $(Z^n_i)_{n \in \mathbb {N}}$ of the form (11.21) such that $\Vert Z^n_i-Z_i\Vert _{\mathbb {B}(\mathbb {T})} \rightarrow 0$ almost surely and in $L_2(\Omega ; C(\mathbb {T}))$ as $n \rightarrow \infty$. In view of Lemma 5.6, by passing through subsequences, we may assume that $(\phi ^{Z^n_i,s}(t,x))_{n\in \mathbb {N}}$ converges almost surely to $\phi ^{Z_i,s}(t,x)$ for i=1,...,M.

For each *n*, we have

11.22 $$\begin{aligned} \mathbb {E}^s f(\phi ^{Z^n_1,s}(t,x),\dots \phi ^{Z^n_M,s}(t,x)) = g(Z^n_1,\dots , Z^n_M). \end{aligned}$$

We can apply the conditional dominated convergence theorem to see that

$$\mathbb {E}^s f(\phi ^{Z^n_1,s}(t,x),\dots , \phi ^{Z^n_M,s}(t,x)) \longrightarrow \mathbb {E}^s f(\phi ^{Z_1,s}(t,x),\dots \phi ^{Z_M,s}(t,x)) \text { a.s.}$$

By Lemma Lemma 5.6 and dominated convergence theorem, it also follows that the function $g:C(\mathbb {T})^M \rightarrow {\mathbb {R}}$ is continuous. Hence, upon taking the limit $n\rightarrow \infty$ in (11.22), we obtain (11.20). □

## Footnotes

1 i.e. ξ is a centered Gaussian family of random variables with $\mathbb {E}(\xi (h)\xi (\bar{h})) = \langle h, \bar{h} \rangle _H$ for all $h,\bar{h} \in H$.

2 In particular:

$$\begin{aligned} \Theta _1(\theta ,\eta )&:= \eta (\theta \phi _1 + (1-\theta )\phi _2) + (1-\eta )(\theta \phi _3 + (1-\theta ) \phi _4),\qquad \Theta _2(\theta )&:= \theta \phi _3 + (1-\theta ) \phi _4, \end{aligned}$$

but this is not important for the proof.
